# Fish and Phytoplankton Exhibit Contrasting Temporal Species Abundance Patterns in a Dynamic North Temperate Lake

**DOI:** 10.1371/journal.pone.0115414

**Published:** 2015-02-04

**Authors:** Gretchen J. A. Hansen, Cayelan C. Carey

**Affiliations:** Center for Limnology, University of Wisconsin-Madison, Madison, Wisconsin, United States of America; CSIR- National institute of oceanography, INDIA

## Abstract

Temporal patterns of species abundance, although less well-studied than spatial patterns, provide valuable insight to the processes governing community assembly. We compared temporal abundance distributions of two communities, phytoplankton and fish, in a north temperate lake. We used both 17 years of observed relative abundance data as well as resampled data from Monte Carlo simulations to account for the possible effects of non-detection of rare species. Similar to what has been found in other communities, phytoplankton and fish species that appeared more frequently were generally more abundant than rare species. However, neither community exhibited two distinct groups of “core” (common occurrence and high abundance) and “occasional” (rare occurrence and low abundance) species. Both observed and resampled data show that the phytoplankton community was dominated by occasional species appearing in only one year that exhibited large variation in their abundances, while the fish community was dominated by core species occurring in all 17 years at high abundances. We hypothesize that the life-history traits that enable phytoplankton to persist in highly dynamic environments may result in communities dominated by occasional species capable of reaching high abundances when conditions allow. Conversely, longer turnover times and broad environmental tolerances of fish may result in communities dominated by core species structured primarily by competitive interactions.

## Introduction

Understanding patterns in species abundance has been a central goal of ecology for several decades [1–4]. Macroecology, the statistical examination of species across large scales [5], has mostly focused on spatial patterns. Temporal components of species abundance distributions were included in early work [6,7], but remain less well studied than spatial patterns, potentially because long-term data from the same ecosystem on entire communities are rare [8]. There is now a resurging interest in temporal patterns of species abundance and community dynamics [9,10], in part because observed analogues between spatial and temporal patterns for many macroecological patterns can provide insight into the ultimate drivers of commonness and rarity [11–13].

Many communities exhibit an occupancy-abundance relationship: generally, more common species (i.e., those observed more frequently) tend to be more numerically abundant than species observed infrequently [14]. This pattern holds true spatially; when sampled across multiple locations, species observed at many sites are generally more abundant than those observed at a small number of sites [15–17]. Temporally, this pattern also holds true: when the same location is sampled multiple times, species observed on many different occasions are typically more abundant than those observed only a few times [9,18–21]. However, relatively few studies have examined this relationship over time, especially at decadal time scales [12,22].

The dramatic differences in the abundance of temporally common versus rare species has led to the hypothesis that communities are composed of two distinct groups: “core” and “occasional” species [9,20,23]. Here, following Magurran and Henderson [9], we refer to temporal definitions of core species as those that appear in all sampling events generally at high abundances, and occasional species as those that appear in one sampling event generally at low abundances (the spatial analogue is "satellite species"; [15]).

Core and occasional species may vary in commonness and abundance because they have fundamentally different life history strategies and mechanisms controlling their abundance [12,24]. Specifically, it has been proposed that the community assembly of core species may be controlled by biotic interactions, with their abundances indicating their relative competitive ability [25,26]. Conversely, occasional species are migrants governed by primarily abiotic processes and dispersal, and their abundances reflect their ability to exploit rare favorable conditions [9,20,27]. Comparing the relative abundance of core and occasional species within a community may provide insight to the factors governing community structure and assembly [12,28]. Communities composed primarily of species with high dispersal rates and fast turnover times relative to sampling frequency may be dominated by occasional species, and structured primarily by environmental variation and dispersal. By comparison, communities that are dominated by species with low dispersal rates and slow turnover times may be dominated by core species and be structured primarily by competitive interactions.

All communities studied to date have exhibited both core and occasional species, with the relative abundance of each group reflecting the importance of biotic and abiotic mechanisms in structuring the abundance distribution of the community on the temporal and spatial scale of sampling [12,19,29]. Within a community sampled over time, these two groups have been distinguished using a variety of statistical methods [9,20,23], the most straightforward of which is a bimodal frequency distribution when occurrence is plotted for a group of co-occurring species, where the x-axis represents frequency of occurrence in the dataset [18,19,29]. Approximately equal numbers of both core and occasional species have been observed in time series of winter annual desert plants [19], prairie plants [29], birds, and grasshoppers [18], while core species appearing in all sampling events were predominant in both estuarine fish [9] and marine ciliate [23] communities. Although they did not explicitly examine core and occasional species, a study of Tabanid flies is the only study on temporal abundance patterns of which we are aware that found predominately occasional species [21].

The relative balance of core and occasional species in a survey is also influenced by sampling effectiveness: i.e., even when a sampling protocol is standardized and extensive, it is likely that some species present in the community will not be detected (i.e., false negatives) [30,31]. Because species at low abundances are less likely to be detected than those at high abundances, occasional species can arise if a species is present but not detected by sampling in each year [1]. The frequency of sampling and sample size can also influence the frequency distribution of species abundances [32]. Short-term studies relative to organism lifespan are expected to produce bimodal frequency distributions with discrete groups of occasional and core species, while longer-term studies are expected to result in unimodal distributions dominated by occasional species and lacking a core group [19,29]. Thus, conclusions based on the proportion of occasional species in a community must consider the potential influences of sampling error and sampling frequency.

In this study, we examined temporal patterns of the relative abundances of two communities comprised of species with strongly contrasting life history strategies (phytoplankton and fish) that co-occurred in a highly variable environment (Table 1). For this analysis, we used a macroecological perspective to focus on the inter-annual temporal variability of abundance. While many studies have examined succession within a year, much less is known about community dynamics among years at decadal scales, primarily due to the absence of data. Inter-annual variability may be an intrinsic property of communities in north temperate latitudes [33], but until recently, relatively few datasets have existed to examine these dynamics.

**Table 1:** An overview of varying physical, chemical, and biological conditions in the water column of Lake Mendota during 1995–2010.

Variable		Minimum value	Median value (± 1 S.D.)	Maximum value
Physical	Schmidt stability (J/m^2^)	0	123 ± 246	790
	Temperature (°C)	0	11.9 ± 6.3	27.6
Chemical	Dissolved organic carbon (mg/L)	0.3	5.7 ± 2.7	15.5
	Dissolved oxygen (mg/L)	0	8.4 ± 4.8	19.6
	Dissolved reactive silica (mg/L)	0.006	2.0 ± 1.7	9.4
	pH	6.3	8.3 ± 0.4	9.5
	Total nitrogen (mg/L)	0.32	0.91 ± 0.57	4.00
	Total phosphorus (mg/L)	0.013	0.12 ± 0.15	1.50
Biological	*Daphnia* abundance (animals/mL)	3.5 × 10^3^	6.4 × 10^5^ ±	9.0 × 10^6^
			1.5 × 10^6^
	Secchi depth (m)	0.6	3.0 ± 2.4	13.4
	Total chlorophyll *a* (μg/L);	0.2	5.1 ± 8.4	60.0
	Total zooplankton abundance (animals/mL)	3.7 × 10^5^	5.3 × 10^6^ ±	4.4 × 10^7^
			6.4 × 10^6^	

Minimum, median (± 1 standard deviation), and maximum observed values from all individual sampling days are given; all data were derived from the LTER monitoring dataset (see: http://lter.limnology.wisc.edu). The method detection limit for dissolved organic carbon is 0.30 mg/L, dissolved reactive silica is 0.006 mg/L, total nitrogen is 0.021 mg/L, and total phosphorus is 0.003 mg/L.

We used a 17-year dataset of phytoplankton and fish communities in a north temperate lake to ask two questions: 1) Do phytoplankton and fish exhibit a temporal occupancy-abundance relationship? and 2) What is the relative balance of core and occasional species within these two assemblages? Phytoplankton have short generation times (hours to weeks); high levels of dispersal via air, wind, water, animal vectors, and from dormant life stages in sediments; and can respond very quickly to perturbations [34]. In addition, phytoplankton represent a particularly interesting case for the study of community dynamics as there are many species with seemingly similar ecological niches, i.e., ‘the paradox of the plankton’ [35]. In contrast, fish in north temperate lakes have much longer generation times (on average, 2.7 ± 1.2 years (1 S.D.); [36]), do not have dormant stages, and are limited in their dispersal to systems connected by surface water or human-assisted means [37,38]. There is large variability among species within both of the phytoplankton and fish communities; however, phytoplankton overall exhibit much faster species turnover (on weekly or shorter time scales; [39]) than fish, which generally exhibit turnover on monthly to yearly scales [31,40]. Based on these differences in life history and dispersal capability, we hypothesized that the phytoplankton would be dominated by occasional species while fish would be dominated by core species, but overall, within both communities, common species would be more abundant, following the occupancy-abundance relationship [12]. We also examined the robustness of our conclusions to the inclusion of false negative species observations to account for potential sampling error.

## Materials and Methods

### Ethics statement

All data in this study were collected as a part of the North Temperate Lakes Long Term Ecological Research Program and are publicly available online (NTL-LTER http://lter.limnology.wisc.edu/). Fish were captured following protocols approved by the University of Wisconsin-Madison Institutional Animal Care and Use Committee (IACUC, permit L00205), and scientific collectors permits (1995–1999: SCP-SD-001-9599; 2000–2004: SCP-SCR-001-0004; 2005–2009: SCP-SCR-001-0509; 2010: SCR 2010-2; 2011: SCR110410) issued by the Wisconsin Department of Natural Resources.

### Site description

Lake Mendota is a eutrophic, north temperate lake located in Madison, Wisconsin, USA (43°6′, 24″N; 89°25′29″W) that has been extensively studied for over a century (for an in-depth description, see [41,42]), and has been part of the North Temperate Lakes Long-Term Ecological Research (LTER) site since 1994 (for maps and other information, see: http://lter.limnology.wisc.edu). The dimictic lake has a mean residence time of 4.5 years, a surface area of 39 km^2^, and a mean and maximum depth of 12 and 25 m, respectively. Within a year, this ecosystem experiences substantial variability in its physics, chemistry, and biology (summarized in Table 1). Lake Mendota is the uppermost lake in the Yahara River chain of lakes, with three lakes downstream [43]. There are no lakes upstream of Mendota, and the incoming streams drain primarily agricultural and urban watersheds on glaciated terrain [43].

### Sampling methods

As part of its routine LTER monitoring, phytoplankton and fish communities in Lake Mendota were sampled during 1995–2011 (for detailed methodological information and data, see: http://lter.limnology.wisc.edu). In brief, phytoplankton from the deep hole were collected biweekly (i.e., every 2 weeks) to monthly during the open-water period with an integrated 8 m tube, pooled into a composite 0–8 m sample, and immediately preserved with gluteraldehyde. More than 400 natural units (cells, filaments, or colonies) were identified to species per sample using an inverted microscope and reported as cell densities mL^-1^ [44]. The biovolume of each natural unit was also calculated in μm^3^. To be comparable with the fish, we conducted all phytoplankton analyses using cell densities because we were primarily interested in phytoplankton relative abundances, not biovolume. Importantly, the same taxonomist was responsible for all phytoplankton identification throughout the sampling period. Cells that were not taxonomically resolved to species were excluded from our analysis.

Fish were collected yearly in late summer with several gear types designed to sample littoral and pelagic habitats following long-term established protocols. Gear consisted of beach seines, minnow traps, and fyke nets at six littoral sites; a boat-mounted electrofishing system for three littoral transects; vertically hung gill nets for two pelagic samples at the deep hole; and a trammel net across the thermocline at two sites [31,45,46]. To avoid bias resulting from any single gear type, catch per unit effort (CPUE) of all gear types were summed for a single metric of relative abundance for each fish species in each year [47]. To ensure consistency in the definition of “species,” three hybrids in the Centrarchidae family were removed from the dataset. Finally, although the relative abundance of two piscivorous fishes (walleye, *Sander vitreus*; and northern pike, *Esox lucius*) were influenced by stocking throughout the study period as part of fisheries management and a biomanipulation project to improve water clarity [41], the exclusion of these two species did not influence overall results, and we retained them in the analysis.

To ease comparison between the two communities and ensure that sufficient sampling effort was expended on both phytoplankton and fish, we constructed species accumulation curves in the R package vegan [48] using the observed data. From the curves, we extrapolated the total number of species in the community using a bootstrap function with 1000 permutations, assuming a random ordering of samples.

To further make the sampling interval of the phytoplankton data comparable with the yearly fish measurements, we pooled all open-water samples collected within the same year. Lake Mendota’s phytoplankton community exhibits substantial seasonal succession [49], so comparing the phytoplankton relative abundance among different individual sampling dates within a year would be biased by succession dynamics. For example, it would be inappropriate to compare phytoplankton relative abundance during the warm summer months and cold early spring or late autumn months because of the large differences in temperature, nutrients, and other factors that alter community assembly patterns. Instead, we determined the maximum observed relative abundance for each species within every year from the pooled open-water samples. During the 17 years of monitoring, phytoplankton were sampled at approximately the same frequency and at the same intervals every year (i.e., phytoplankton collection began immediately after ice-off in the spring every year in March or April and continued until just before ice-on in December or January); hence, the phytoplankton community should be comparable among years.

### The influence of sampling error

For the analyses presented here, we focused on the observed composition and relative abundance of species in the phytoplankton and fish communities, as has been done in the vast majority of previous studies examining species abundance distributions. Observed species data are influenced by both the actual species abundances as well as sampling error in monitoring, such as the failure to detect or identify a species in a given year when it is actually present due to its patchiness or rarity, i.e., a false negative [30]. Given the small volume of water sampled for phytoplankton identification relative to the large size of the lake and the patchiness of phytoplankton abundance, false negatives may be particularly problematic for the phytoplankton community observations. To examine the influence of sampling error, we conducted all analyses twice, first using the observed data, and second using resampled data derived from Monte Carlo simulations.

Following [30], we conducted Monte Carlo simulations for both phytoplankton and fish communities based on the probability of detecting individual species. Because we were interested in the relationship between relative species abundance and commonness, we assumed a uniform distribution of species across time. We assumed that the total species pool within both communities was present in Lake Mendota every year, although we were unable to detect every species due to sampling error. We assumed that the sampling error of detection varied among species independently of their density, according to a unique probability *p* for each species, in which *p* = number of years a species was observed divided by the total number of years sampled (i.e., 17). Consequently, observed abundance did not directly determine a species’ *p*, but may have influenced it by altering the number of years a species was observed.

To generate resampled data for each community, we created a matrix of the relative abundance of each species in each sampling year using the probability *p* to sample observed relative abundances with replacement. This process was simulated 10,000 times to generate a distribution of resampled relative abundances and numbers of years each species was observed over the 17-year monitoring period that included the effect of sampling error. The maximum relative abundance of each species in each of the 10,000 simulations was used to calculate the standard deviation of relative abundance attributable to sampling error [30].

To further assess the effects of false negatives on our results, we examined how increased detection of each species in additional years would influence our conclusions. We repeated the Monte Carlo simulations 16 additional times, each time sequentially adding 0.0588 (1/17 years) to each species’ *p* to generate new distributions of resampled relative abundance as the number of total years a species was observed increased by one (only for species with *p* < 1). Thus, for the 16^th^ iteration (when 0.9412, or 16/17 years was added to each species’ *p*), all species were observed every year and every species’ *p* = 1.

### Statistical analyses

We conducted all analyses on both the observed and resampled data. Within each community and year, we assigned each species a rank based on their maximum observed or resampled relative abundance on any sampling day in that year (highest relative abundance species = rank 1). To assess whether a relationship existed between observed temporal occurrence and relative abundance, we regressed the maximum observed abundance (log_10_-transformed) in any year against the number of years a species was observed [9,18,19,21]. For the resampled data, we calculated these regression statistics for each of the 10,000 resampled datasets.

To assess the relative frequency of core and occasional species, the distributions of both the observed and resampled species occurrences over time were plotted for each community. For this analysis, the proportion of species occurring in each number of years (1–17) was examined; this is the temporal analogue to a spatial metapopulation analysis [50] used to assess the relative frequency of core and satellite species [18,29]. Although numerous methods of distinguishing between core and occasional species have been used [9,20,23], we sought to maximize the contrast between the two groups by defining core species as those appearing in each year of sampling, and occasional species as those appearing in only one year. We repeated this analysis for both communities using the 16 additional species relative abundance matrices generated by sequentially increasing the probability that a species was detected (see above).

Finally, to assess the influence of temporal frequency on static depictions of community structure, we generated single rank abundance distributions (RAD) for each community using both the observed and resampled relative abundances. In this case, we ranked species based on their highest observed or resampled relative abundances observed in the entire time period, and examined the location of core vs. occasional species within the RAD [12,32]. All analyses were conducted in R v. 3.0 (R Development Core Team 2012).

## Results

### Observed relative abundance

**Fig 1:**
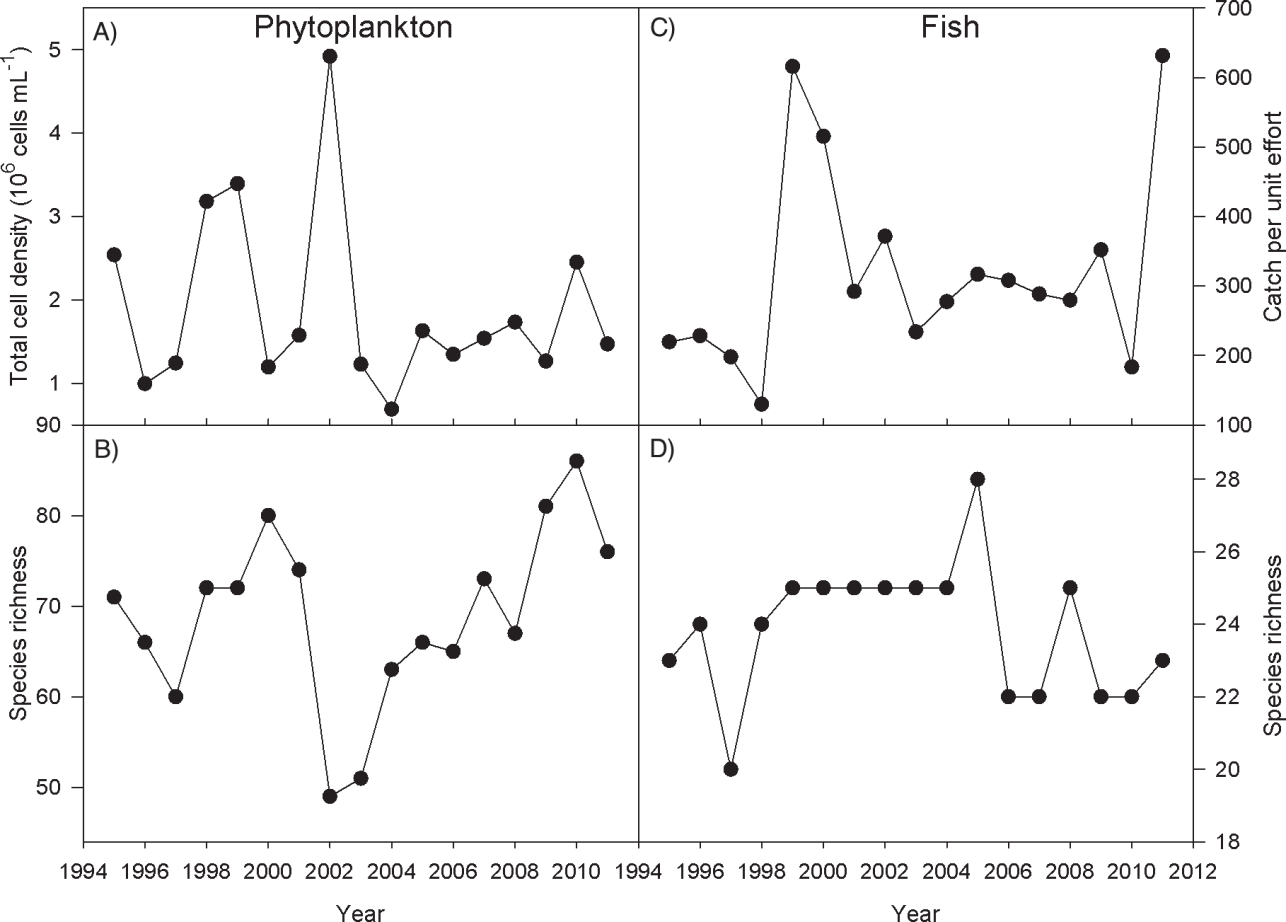
Total observed relative abundance (A) and species richness (B) in each year for phytoplankton (units = cells/mL); and total observed relative abundance (C) and species richness (D) in each year for fish (units = fish/gear for all gears combined).

During the open water period in 1995–2011, we observed in total 254 species of phytoplankton and 36 species of fish. The total (summed) observed relative abundance of both communities varied substantially over time: phytoplankton and fish exhibited greater than five-fold differences in their relative abundance (in cells/mL and CPUE, respectively) among years (Fig. 1). Phytoplankton observed richness was variable, varying from 49 to 86 observed species among years, while fish observed richness varied from 20 to 28 species among years. Based on species accumulation curves, we estimated that over the 17 years we sampled 88% of the total number of phytoplankton species (254 ± 6 out of 290 total, 1 S.D.), and 97% of the total number of fish species (36 ± 1 out of 37 total) estimated to be present in the lake over the 17-year period (S1 Fig.).

**Fig 2:**
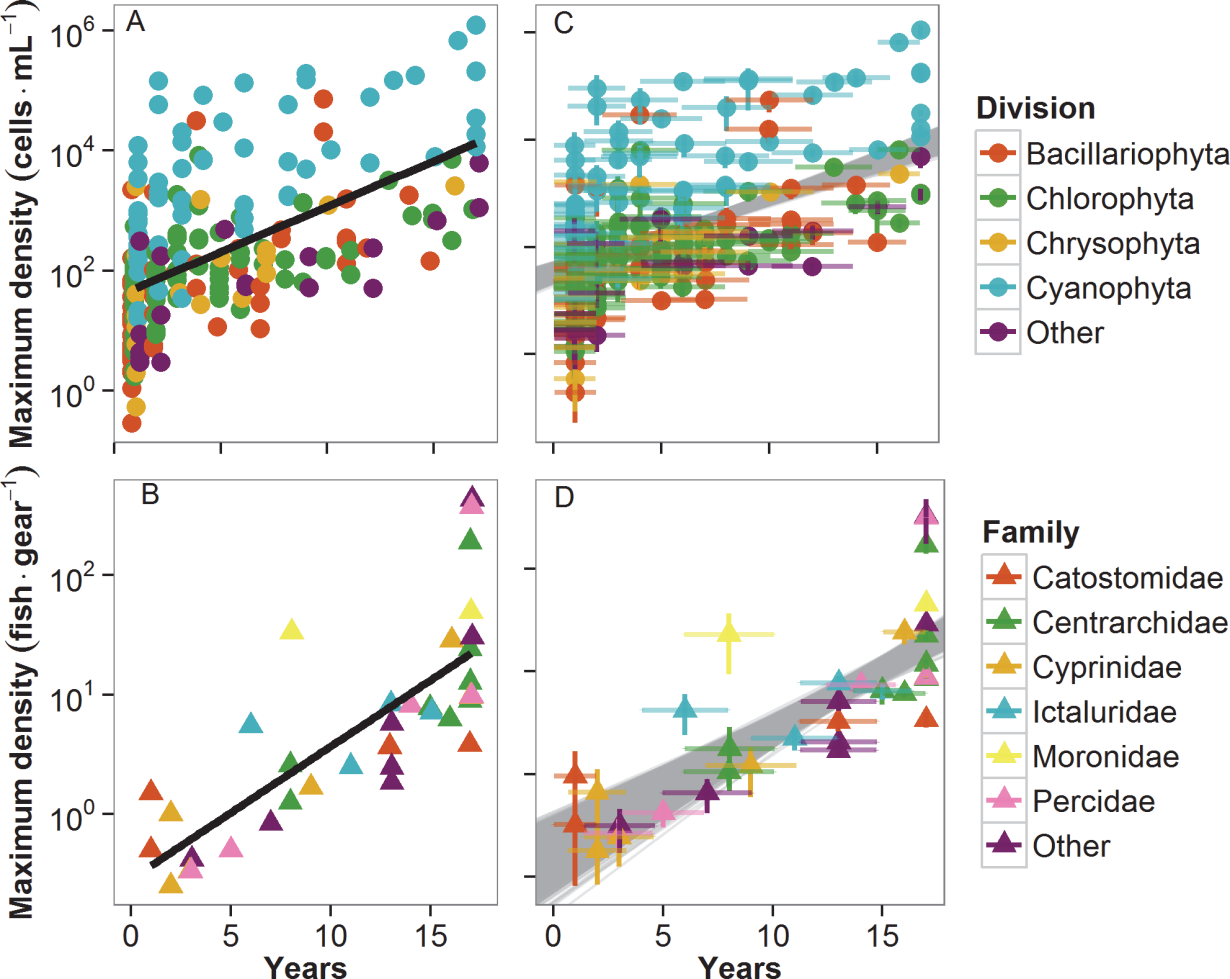
Maximum relative abundance (in any year) of each species in Lake Mendota from 1995–2011 as a function of the number of years that species appeared in the dataset for (A) observed phytoplankton (Log_10_(phytoplankton maximum density) = 1.56 + 0.15 × years in dataset, adjusted R^2^ = 0.32, p<0.00001); (B) Observed fish (Log_10_(fish maximum density) = -0.54 + 0.11 × years in dataset, adjusted R^2^ = 0.60, p<0.00001); (C) Resampled phytoplankton (median (minimum—maximum) regression statistics from resampled data: Log_10_(phytoplankton maximum density) = 1.52(1.28–1.73) + 0.13(0.11–0.16) × years in dataset, adjusted R^2^ = 0.29(0.20–0.42), all p<0.00001); and (D) Resampled fish (Log_10_(fish maximum density) = -0.73(-1.38–0.36) + 0.12(0.09–0.15) × years in dataset, adjusted R^2^ = 0.59(0.36–0.79); all p<0.00001). The resampled maximum abundances for (C) and (D) are presented as means and standard deviations of the maximum abundances observed in 10,000 Monte Carlo simulations, and all 10,000 resampled regression lines are shown. “Other” includes phytoplankton divisions Cryptophyta, Euglenophyta, Pyrrhophyta, and Xanthophyta, and fish families Amiidae, Atherinopsidae, Cottidae, Esocidae, Lepisosteidae, Salmonidae, and Scianidae.

Both the observed phytoplankton and fish communities exhibited positive temporal abundance-occupancy relationships, demonstrated by significant positive slopes relating maximum density of a species to the number of years in which it was observed (Fig. 2A,B; Log_10_(phytoplankton maximum density) = 1.56 + 0.15 × years in dataset, adjusted R^2^ = 0.32, p<0.00001; Log_10_(fish maximum density) = -0.54 + 0.11 × years in dataset, adjusted R^2^ = 0.60, p<0.00001). This relationship was robust both within phytoplankton divisions and fish families, as well as across the aggregated community for both phytoplankton and fish.

Most species with the highest observed relative abundances were core species (i.e., species present in all 17 years of sampling). This pattern was especially apparent for fish (Fig. 2B), while relative abundance was generally more variable for phytoplankton regardless of their permanence in the dataset (Fig. 2A). For example, three occasional phytoplankton species (i.e., species observed in only one year during the time series) were very abundant (ranked in the top 13^th^ percentile of relative abundance for those years), and some core species were not abundant (rankings in the 70^th^ percentile of relative abundance for some years; S1 Table). Conversely, no occasional fish species ever ranked higher than the top 40^th^ percentile, although similar to phytoplankton, some core species did have low abundance (ranks in the 80^th^ percentile for some years; S1 Table).

**Fig 3:**
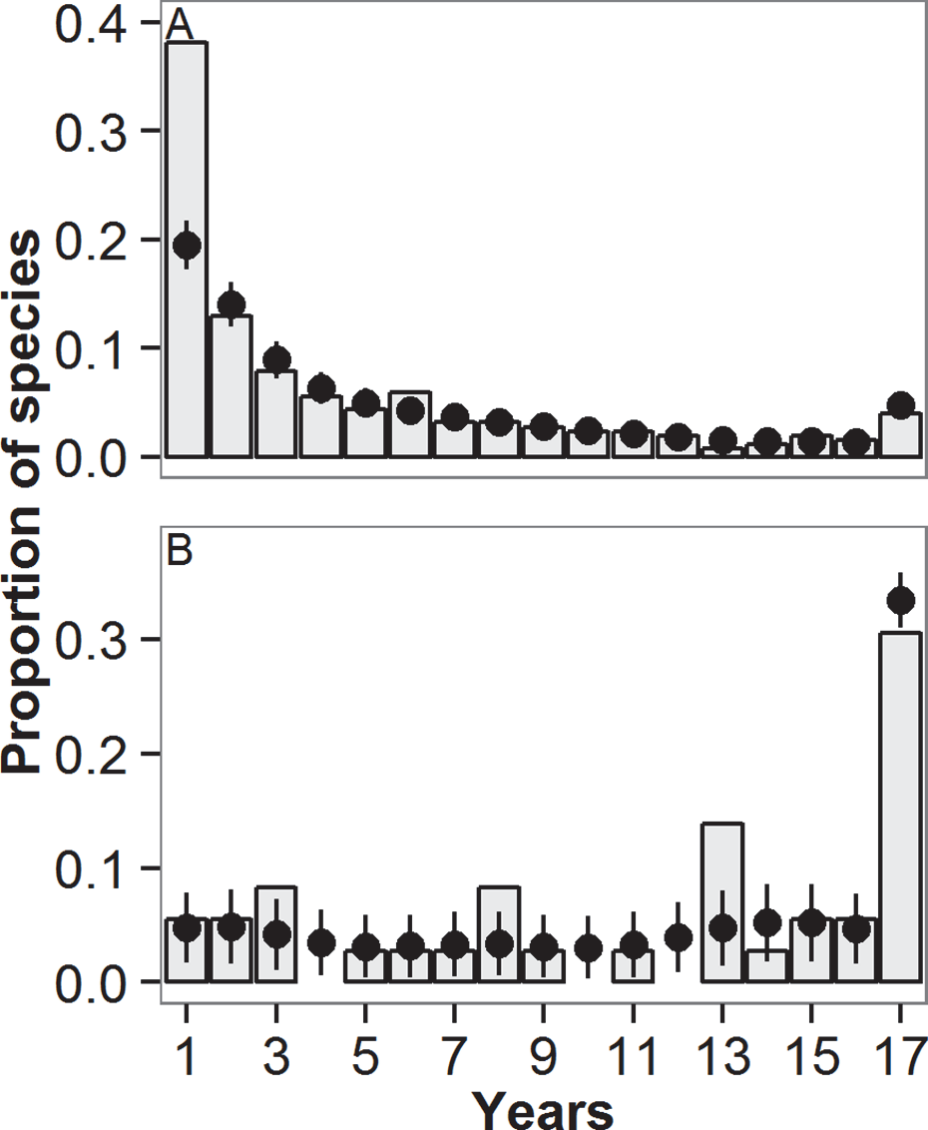
Histogram of the proportion of species appearing in each number of years (1–17) in Lake Mendota from 1995–2011, for (A) phytoplankton and (B) fish. The columns represent the observed species proportions, and the circles represent the mean resampled species proportions with standard deviations from 10,000 Monte Carlo simulations.

Overall, the observed phytoplankton community was dominated by occasional species with few core species, while the observed fish community was dominated by core species with few occasional species (Fig. 3). The frequency distribution of the observed number of years in which a species occurred was unimodal in both communities. For phytoplankton, 38% of species (N = 97) appeared in only 1 year (occasional species), and only 4% (N = 10) occurred in all 17 years (core species). For fish, 31% of species (N = 11) appeared in all 17 years, and 6% (N = 2) were observed in only 1 year.

**Fig 4:**
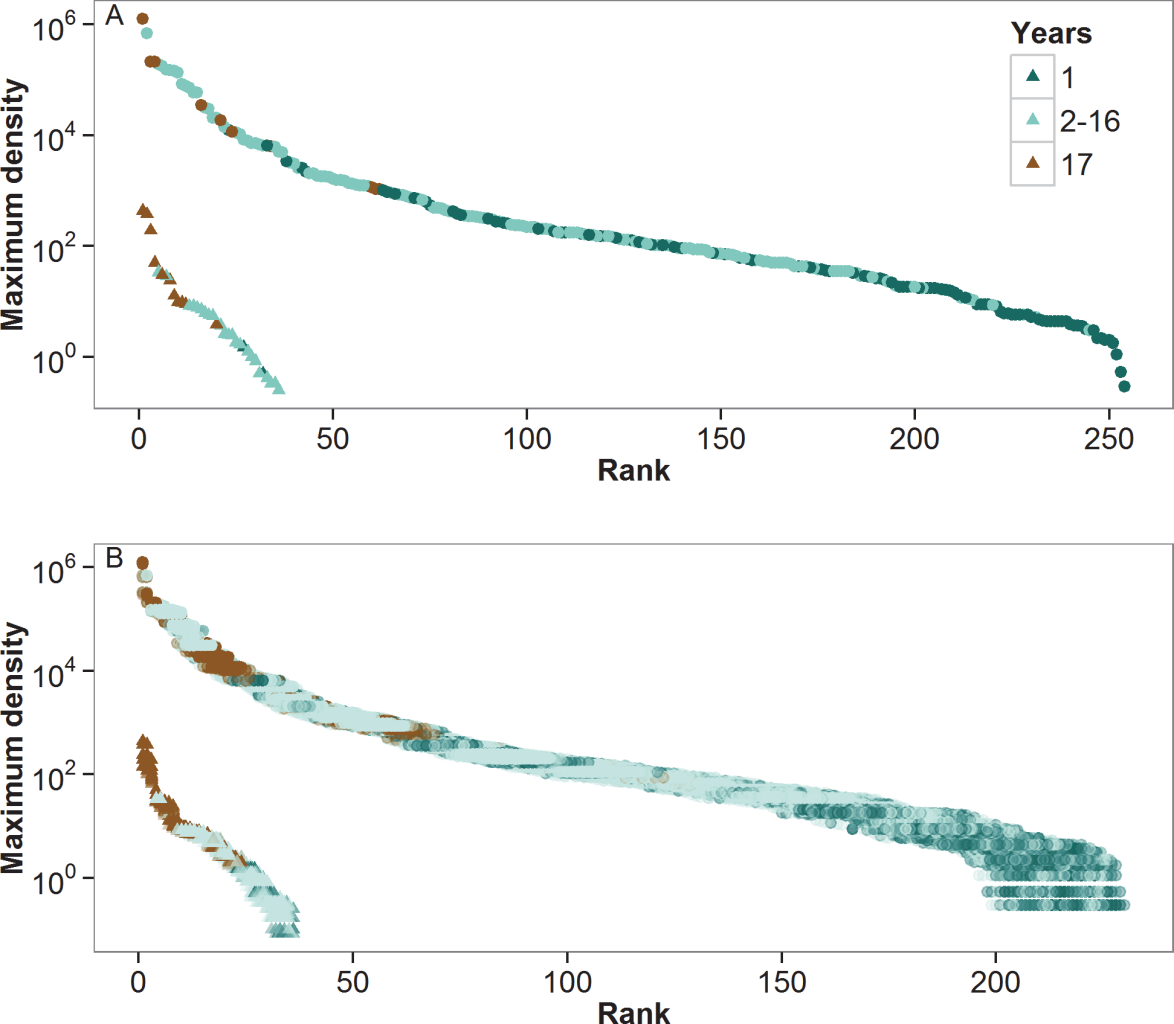
Rank abundance distribution of (A) observed and (B) resampled phytoplankton (circles) and fish (triangles) densities, where the colors correspond to the number of years in which a species appeared in the Lake Mendota dataset from 1995–2011. Ranks are based on maximum observed abundance over the entire 17-year period. The resampled distributions for the phytoplankton and fish were derived from 1,000 Monte Carlo simulations for clarity in presentation.

The difference in the relative balance of core and occasional species in the two observed communities can also be observed in their static rank abundance distributions (RAD). When aggregated across all years, the RAD of observed phytoplankton data does not show distinct groupings of both core and occasional species (Fig. 4A). Occasional phytoplankton species were dispersed widely throughout the RAD, and core species were generally lacking. For fish, core species grouped together fairly closely at the high end of the RAD, with the small number of occasional species scattered within the RAD.

### The influence of sampling error

**Fig 5:**
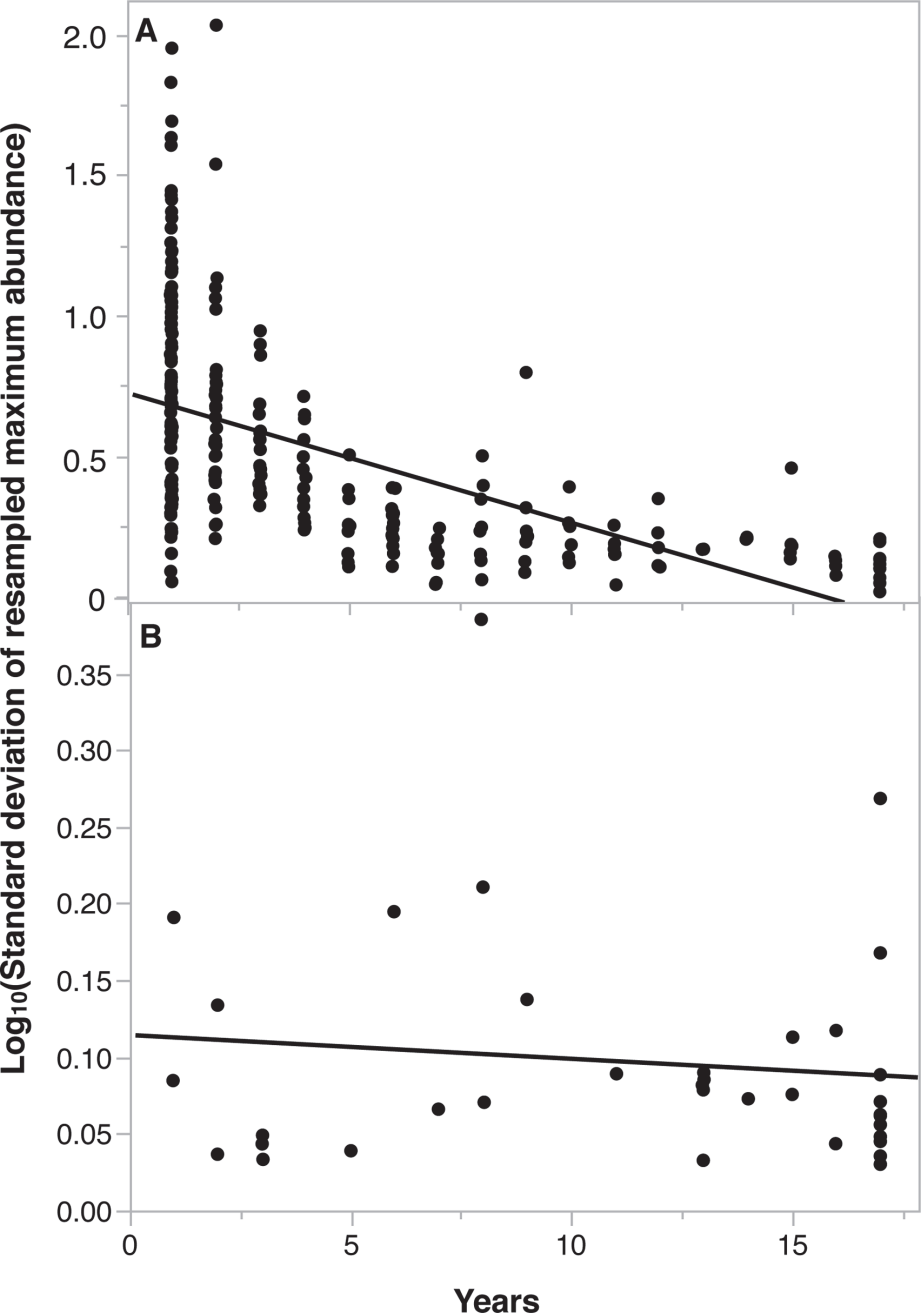
The Log_10_-transformed standard deviation in maximum abundance for (A) phytoplankton (Log_10_-standard deviation of resampled maximum phytoplankton relative abundance = 0.72–0.05 × years in dataset, adjusted R^2^ = 0.31, p<0.00001); and (B) fish (Log_10_-standard deviation of maximum resampled fish relative abundance = 0.11–0.002 × years in dataset, adjusted R^2^ = 0.01, p = 0.49), ordered by the number of years each species was present in the observed dataset. The standard deviations were derived from 10,000 Monte Carlo simulations for both communities.

We observed significantly higher sampling error in the relative abundance of occasional phytoplankton than in core phytoplankton species in the resampled dataset (Fig. 5A; Log_10_(standard deviation of resampled maximum phytoplankton relative abundance) = 0.72–0.05 × years in dataset, adjusted R^2^ = 0.31, p<0.00001). By comparison, the number of years observed in the dataset did not affect the variation in resampled maximum relative abundance for the fish species (Fig. 5B; Log_10_(standard deviation of maximum resampled fish relative abundance) = 0.11–0.002 × years in dataset, adjusted R^2^ = 0.01, p = 0.49). The sampling error in the occasional phytoplankton species was evident in the histogram of species proportions (Fig. 3A), as there were fewer occasional phytoplankton species in the resampled relative abundance dataset than the observed relative abundance dataset.

Regardless of the sampling error, however, we observed the same general patterns in the resampled relative abundance data as the observed relative abundance data for both communities. Overall, the resampled phytoplankton community was dominated by occasional species, and the fish community was dominated by core species (Figs. 3,4). Both phytoplankton and fish communities exhibited positive temporal abundance-occupancy relationships in resampled data (Fig. 2C,D; Median (minimum—maximum) regression statistics from resampled data: Log_10_(resampled phytoplankton maximum abundance) = 1.52(1.28–1.73) + 0.13(0.11–0.16) × years in dataset, adjusted R^2^ = 0.29(0.20–0.42), all p<0.00001; Log_10_(fish maximum density) = -0.73(-1.38–0.36) + 0.12(0.09–0.15) × years in dataset, adjusted R^2^ = 0.59(0.36–0.79); all p<0.00001). The temporal abundance-occupancy relationships based on resampled data were robust both within phytoplankton divisions and fish families. The core species of both communities tended to exhibit the highest resampled relative abundances, with much more variability in the phytoplankton relative abundances than in those of the fish (Fig. 4B). As with the observed data, the resampled phytoplankton RAD did not exhibit distinct groupings of core and occasional species (Fig. 4B).

**Fig 6:**
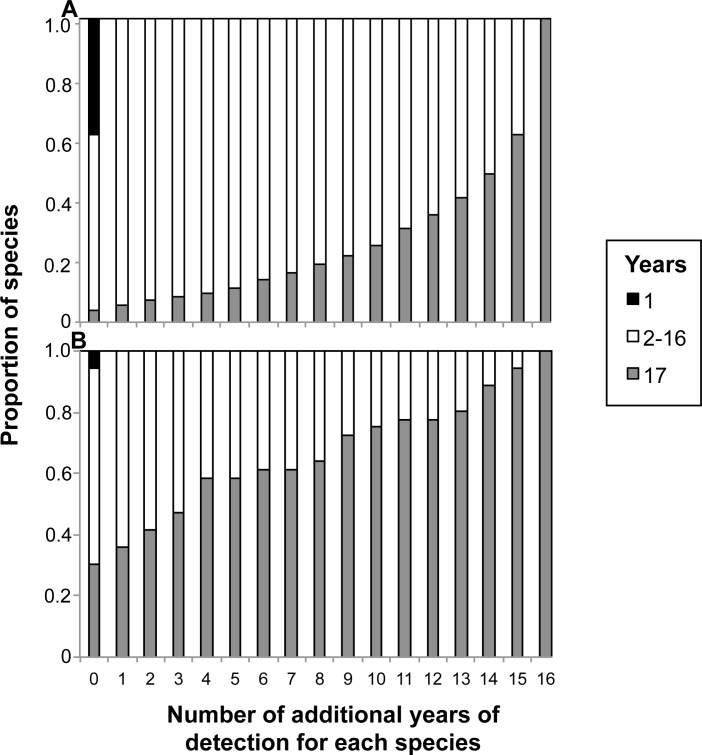
The proportion of resampled species that appeared in 1 year (occasionals), 2–16 years, or 17 years (cores) for (A) phytoplankton and (B) fish when the simulated number of years of a species’ detection was sequentially increased by one year. The zero column refers to the observed number of species from the observational data.

The general patterns in phytoplankton and fish relative abundance were robust even to simulated increases in the number of years a species was detected to account for the possible influence of false negative observations. The phytoplankton community continued to exhibit few core species as we simulated additional years of species detections, while the fish community exhibited an even greater dominance of cores (Fig. 6). For example, if all phytoplankton species were detected an additional 5 out of 17 years, representing substantial sampling error, the proportion of core species in the phytoplankton community only increased from 4% to 11%. By comparison, increasing the fish species detections by five years increased the proportion of core species from 31% to 58%. Fifteen additional years of detection for all species were required to increase the proportion of core species in the phytoplankton community to >50% of all species, whereas only four additional years of species detections were required to increase the proportion of core species in the fish community to >50%.

## Discussion

In a plea for more studies examining the temporal components of species abundance distributions, Magurran [12] suggested that comparative studies across different taxa and systems would provide insight into the processes shaping abundance distributions. Our analysis provides a new perspective on decades of aquatic research by leveraging a rare long-term dataset documenting the relative abundance patterns of two assemblages from the same system over multiple decades to highlight dramatically different temporal patterns in species occurrence. Surprisingly, neither community exhibited a bimodal distribution of temporal frequency representing two distinct groups of core and occasional species, as has previously been observed for many other communities [18,19,29,32]. Rather, occasional species observed in a single year dominated the phytoplankton community, while core species observed in all 17 years dominated the fish community.

Our results support the ubiquity of a positive temporal occupancy-abundance relationship [9,18,19,21]. In both communities, the relative abundance of species increased by a similar magnitude as the number of years of species observation increased (Fig. 2). For each additional year a species was observed, its maximum relative abundance increased by 15% for phytoplankton (resampled range: 11–16%) and 11% for fish (resampled range: 9–15%). However, the temporal occupancy-abundance relationship within the phytoplankton community was much more variable than that of the fish community (Fig. 2). Occasional phytoplankton species were capable of reaching high relative abundances, while some core phytoplankton species had very low relative abundances in some of the years in which they were observed (S1 Table).

The few core phytoplankton species in Lake Mendota accounted for a relatively small percentage of total community abundance. The 10 observed core phytoplankton species accounted for (on average) 55% ± 22% (1 S.D.) of the total observed relative abundance in any given year (range over 17 years was 25%–90%). By comparison, the 11 observed core fish species composed (on average) 92% ± 0.04% of total fish relative abundance (range = 81%–98%), which is similar to the dominance of core species observed in other studies. For example, three estuarine fish species accounted for over 70% of the total abundance in any given year in a 21-year study [9], 11 core species composed 85–90% of individuals in a tintinnid ciliate community of the Mediterranean Sea sampled over four weeks [23], and three core species were responsible for 77% of the horse and deer flies observed in a 37-day study [21]. At the same time, 38% of observed phytoplankton and 19% of the resampled phytoplankton were occasional species, which is similar to the percentage of occasional species observed in communities in other studies (5–32%). Lake Mendota fish fall at the low end of this range, with only 6% of observed fish and 5% of resampled fish species observed in just one year.

As in any study of species presence and absence, our observed results assume that sampling was sufficient to detect rare species when they were present. However, it is possible that absences in the observed dataset could be a result of sampling failure to detect a species. The issue of non-detection was particularly important for occasional phytoplankton species (Fig. 3A), because many of the occasional phytoplankton species were observed at high relative abundances in the one year they were sampled. As a result, the failure to detect such an occasional species in the resampled data resulted in high estimated sampling error (Fig. 5A). In contrast, the occasional fish species were only present at low abundances when observed, so missing these species in the resampled data resulted in relatively low sampling error.

Importantly, our conclusions are robust to false negative observations. The resampled data show a similarly strong temporal abundance-occupancy relationship as the observed data for both communities (Fig. 2), and demonstrate that the phytoplankton community has few core species and many occasional species (Figs. 3,4). While sampling error is likely to affect the number of true occasional species, especially for the phytoplankton, our conclusions regarding the absence of core species for phytoplankton are robust. Rare phytoplankton species would have to be present but undetected in 15 of the 17 sampling years to shift the phytoplankton community to dominance by core species (Fig. 6).

The life-history traits of phytoplankton and fish combined with the dynamic environment of Lake Mendota may help explain the high proportion of occasional phytoplankton species and core fish species. Occasional species are specialists that take advantage of rare environmental conditions [27] or migrants that are temporarily passing through an ecosystem [9]. In habitats with high productivity and high disturbance, such as Lake Mendota, phytoplankton species are adapted to quickly respond to transient conditions to convert resources into biomass [34,51]. The productive and dynamic conditions in Lake Mendota provide a large number of niche opportunities that species with high turnover rates and dispersal capacities can exploit [16,52]. Each set of conditions represents a niche opportunity that these species can take advantage of almost immediately until the conditions change again. Furthermore, phytoplankton have a large species pool and high rates of dispersal, which both promote high species turnover. These traits are predicted to result in large numbers of occasional species [12], which we observed.

The importance of both dynamic environmental conditions and high dispersal and turnover rates for producing elevated numbers of occasional species is illustrated by the relative lack of occasional species in a previous study of the temporal abundance patterns of ciliates in the Mediterranean Sea [23]. Although species in this community have similarly high turnover and capacity to respond to changing conditions, core species were prominent, with only 5% of species occurring in one sample. However, Dolan et al. [23]’s study consisted of 18 samples across 4 weeks, and although this represents ~30 generation times for these species, the physical and chemical environment they sampled was far less variable than Lake Mendota (see [53] for environmental variables).

In contrast to the pattern observed in the phytoplankton, the fish community in Lake Mendota consisted of a large number of core species and very few occasional species, in spite of the highly dynamic conditions. Many of the observed fish species (see S1 Table for species list) are diet generalists, and all can withstand large ranges of temperature and light conditions. Furthermore, these fish have generation times of 1–6 years ([36]; see also http://www.fishbase.org/), and are thus not capable of much population turnover as conditions change between yearly sampling dates. Fish are also much more limited than phytoplankton in their ability to disperse among lakes, restricted by surface water connections and human-mediated transport [37,38]. The two occasional fish species observed in only one year – shorthead redhorse (*Moxostoma macrolepidotum*) and silver redhorse (*Moxostoma anisurum*) (See S1 Table) – are both primarily riverine species that occasionally enter lake habitats to feed [36], and thus fit the expectation of occasional species as migrants taking advantage of rare conditions [9,20,27]. The life history traits of this fish community are predicted to produce high numbers of core species structured by competitive interactions [12], unlike phytoplankton communities, where high levels of disturbance and environmental forcing results in phytoplankton communities in various stages of disequilibrium [34,39], thereby preventing core species from dominating.

The temporal duration of sampling plays an important role in abundance distributions [12,19,29,32]. Specifically, studies spanning long durations relative to the longevity of study organisms are more likely to generate unimodal frequency distributions with high numbers of occasional species, while studies of short duration relative to organism turnover time are more likely to generate bimodal distributions with distinct groupings of core and occasional species [19,26,54]. Although our sampling duration for fish was shorter relative to their generation time of 1–6 years, we did not observe a bimodal distribution of fish, which is likely due to their limited dispersal capacity [12], and their low species richness (as also observed by [29]); suggesting that species abundance within the Lake Mendota fish community is structured predominantly by biotic interactions. However, our analysis of 17 years of phytoplankton relative abundance with generation times of hours to days [34] did produce a unimodal distribution dominated by occasional species, suggesting that the sampling frequency may have contributed to this finding.

**Fig 7:**
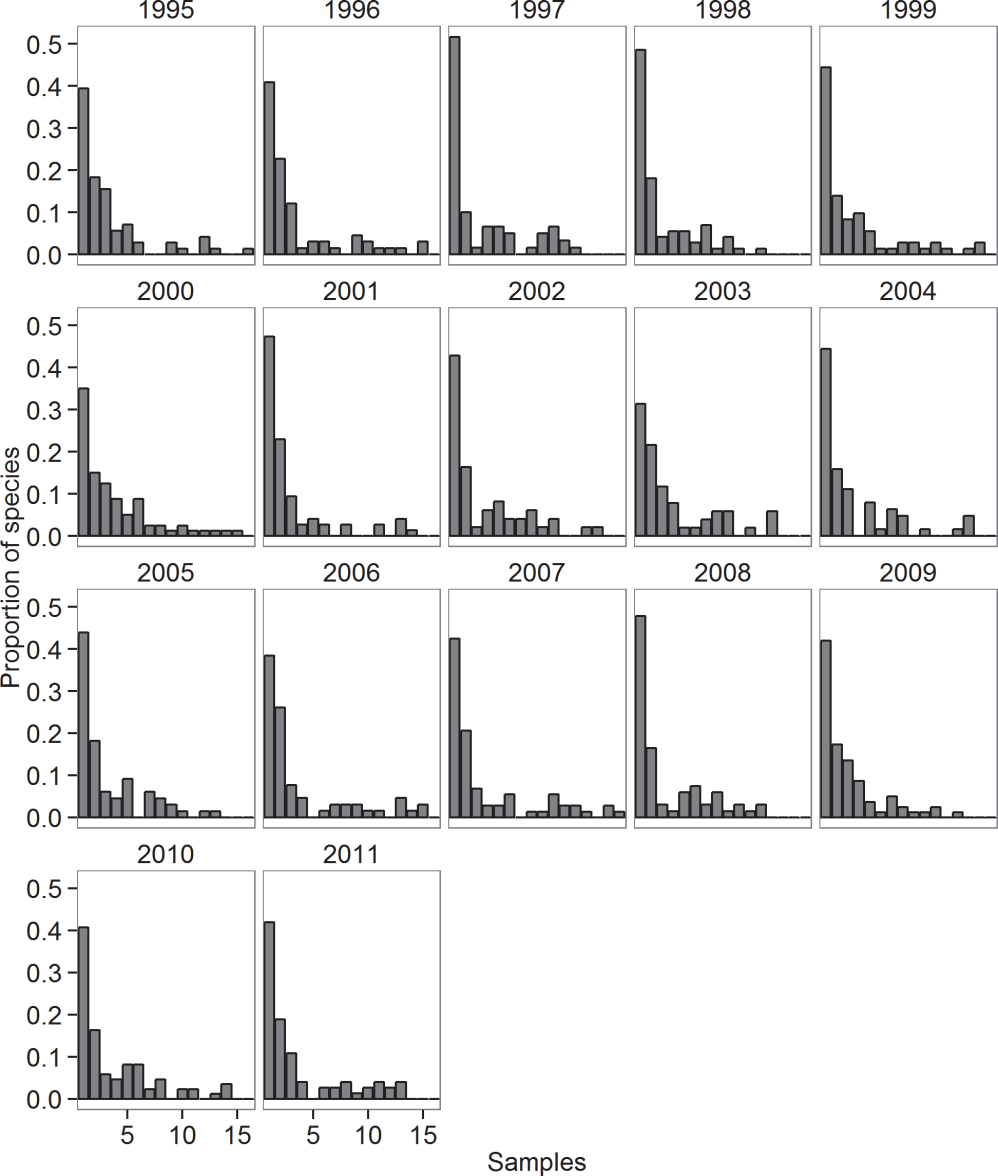
Proportion of observed phytoplankton species occurring in varying numbers of samples observed within each year from 1995–2011.

To investigate if sampling scale altered the likelihood of observing occasional phytoplankton species, we conducted two additional analyses. First, we examined the frequency distributions of observed phytoplankton relative abundance within each year, using individual sampling dates as replicates (Fig. 7) and found similarly unimodal frequency distributions to the inter-annual analysis (Fig. 3), with 30–50% of all observed species occurring in only one sample in a given year. These samples were generally collected at two-week intervals, which still represents several generations for most phytoplankton; however, the existence of unimodality across multiple temporal scales supports our original finding that the Lake Mendota phytoplankton are dominated by occasional species. Second, in lieu of having generation times for each of the 254 phytoplankton species, we compared the biovolume of each species with the number of years it was observed in the 17-year dataset. Larger phytoplankton have longer generation times [34,55]: for example, *Gloeotrichia echinulata*, a cyanobacterium that appears in Lake Mendota, produces colonies that are 1–3 mm in diameter and can survive in the water column up to 21 days [56,57]. Thus, if sampling frequency relative to generation time was the primary factor responsible for the classification of phytoplankton species as occasionals, we would expect a positive relationship between phytoplankton generation time (as size) and commonness. Contrary to those expectations, we observed no significant relationship between mean phytoplankton biovolume and the number of years that it appeared in the dataset (p = 0.28). The largest phytoplankton species with the longest generation times were not core species: e.g., *G*. *echinulata* was only observed in 4 out of 17 years. These data suggest that our results are robust to sampling frequency.

## Conclusions

Examining multiple communities with contrasting life history strategies and dispersal abilities in the same environment can provide insight into mechanisms structuring communities at different taxonomic levels while controlling for environmental differences [38]. In this study of the temporal abundance patterns of phytoplankton and fish, we demonstrate that both groups exhibit positive temporal abundance-occupancy relationships. However, phytoplankton and fish exhibit opposite patterns of temporal frequency, despite occurring in the same dynamic environment. These results are robust to false negative observations, and indicate that the phytoplankton community is dominated by species that appear for only one year while the fish community is dominated by species that are present every year. We hypothesize that their divergent life histories and capacities to respond to variation can explain these differences. Our study is purely empirical, and does not allow us to draw conclusions about the relative merits of the multitudinous theories of community abundance [38]. However, empirical studies examining the temporal abundance patterns of communities with contrasting life histories over long time scales are rare, and the differences and similarities among communities identified here will aid in the quest to determine whether the same processes structuring spatial abundance patterns can also apply to temporal abundance patterns [10]. As phytoplankton and fish populations change as a result of climate, eutrophication, and other anthropogenic factors [58,59], assessing the role of these stressors against a background of baseline change will require an understanding of the dynamic nature of these communities [8].

## Supporting Information

S1 Fig Species accumulation curves of phytoplankton and fish. (TIFF)

S1 Table Fish and phytoplankton species included in this analysis, with density (catch per gear type for fish and cells per mL for phytoplankton), rank for that year, number of years in dataset, and relative abundance percentile for that year. (DOCX)
