# Supplementary figures and images for: Fish and Phytoplankton Exhibit Contrasting Temporal Species Abundance Patterns in a Dynamic North Temperate Lake
Source: PLoS One. 2015 Feb 4;10(2):e0115414. doi: 10.1371/journal.pone.0115414 (PMC4317179; doi:10.1371/journal.pone.0115414)

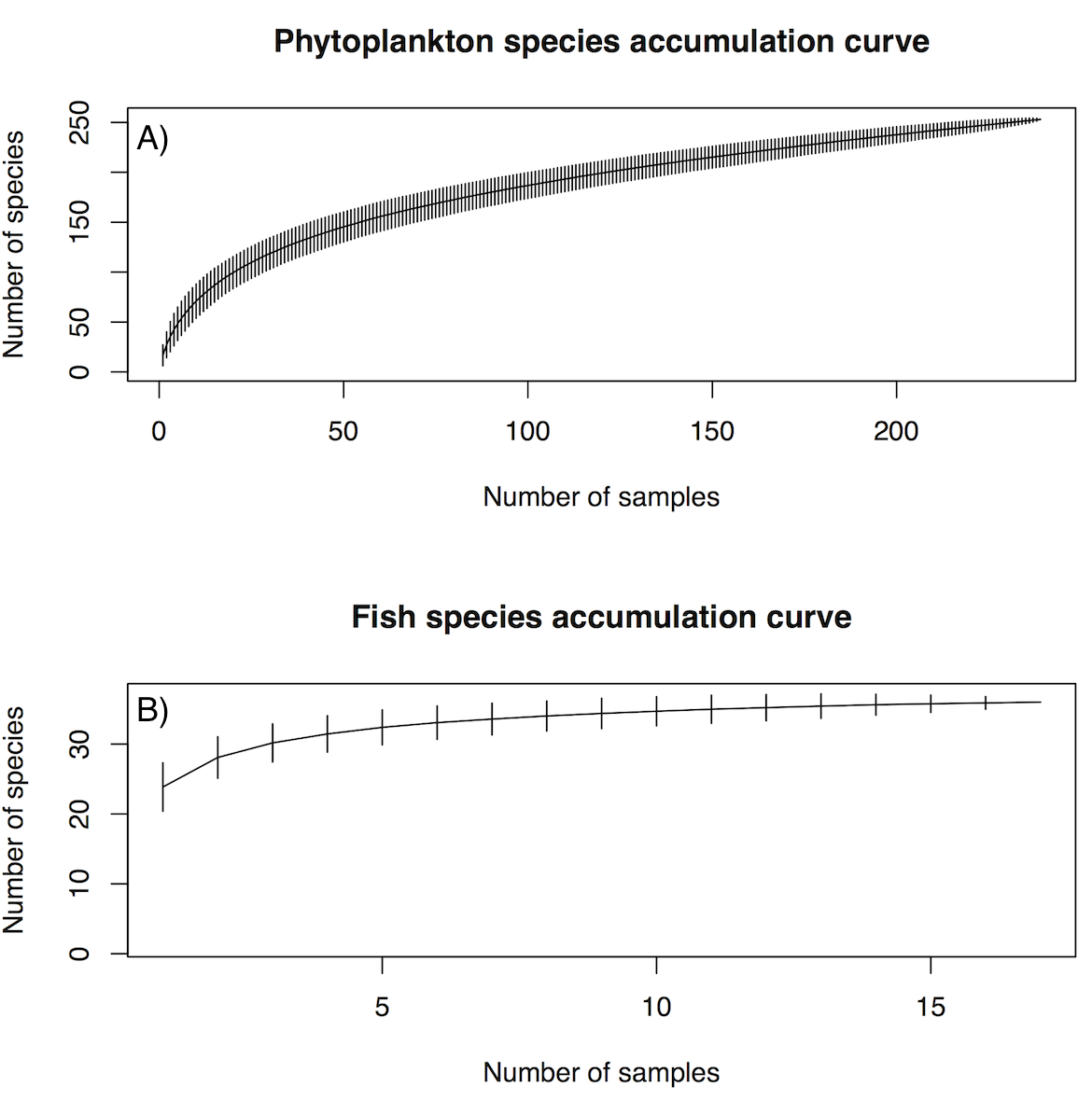

Supplement: S1 Fig — (TIFF) [file pone.0115414.s001.tiff]
