# Supplementary material for: Fish and Phytoplankton Exhibit Contrasting Temporal Species Abundance Patterns in a Dynamic North Temperate Lake
Source: PLoS One. 2015 Feb 4;10(2):e0115414. doi: 10.1371/journal.pone.0115414 (PMC4317179; doi:10.1371/journal.pone.0115414)
Supplement: S1 Table — (DOCX) [file pone.0115414.s002.docx]

Supporting Table 1. Fish and phytoplankton species included in this analysis, with density (catch per gear type for fish and cells per mL for phytoplankton), rank for that year, number of years in dataset, and abundance percentile for that year.

| Community | Species | Family or Division | Year | Density | Rank | | Years in dataset | Percentile |
| --- | --- | --- | --- | --- | --- | --- | --- | --- |
| Fish | Bigmouth Buffalo (*Ictiobus cyprinellus*) | Catostomidae | 1997 | 0.33 | 15 | 13 | | 75 |
| Fish | Bigmouth Buffalo (*Ictiobus cyprinellus*) | Catostomidae | 1998 | 0.33 | 20 | 13 | | 83 |
| Fish | Bigmouth Buffalo (*Ictiobus cyprinellus*) | Catostomidae | 1999 | 1.83 | 16 | 13 | | 64 |
| Fish | Bigmouth Buffalo (*Ictiobus cyprinellus*) | Catostomidae | 2001 | 0.50 | 22 | 13 | | 88 |
| Fish | Bigmouth Buffalo (*Ictiobus cyprinellus*) | Catostomidae | 2002 | 3.00 | 15 | 13 | | 60 |
| Fish | Bigmouth Buffalo (*Ictiobus cyprinellus*) | Catostomidae | 2003 | 1.50 | 17 | 13 | | 68 |
| Fish | Bigmouth Buffalo (*Ictiobus cyprinellus*) | Catostomidae | 2004 | 0.67 | 20 | 13 | | 80 |
| Fish | Bigmouth Buffalo (*Ictiobus cyprinellus*) | Catostomidae | 2005 | 0.67 | 21 | 13 | | 75 |
| Fish | Bigmouth Buffalo (*Ictiobus cyprinellus*) | Catostomidae | 2006 | 0.67 | 19 | 13 | | 86 |
| Fish | Bigmouth Buffalo (*Ictiobus cyprinellus*) | Catostomidae | 2007 | 0.33 | 21 | 13 | | 95 |
| Fish | Bigmouth Buffalo (*Ictiobus cyprinellus*) | Catostomidae | 2008 | 3.67 | 13 | 13 | | 52 |
| Fish | Bigmouth Buffalo (*Ictiobus cyprinellus*) | Catostomidae | 2009 | 0.08 | 21 | 13 | | 95 |
| Fish | Bigmouth Buffalo (*Ictiobus cyprinellus*) | Catostomidae | 2010 | 1.33 | 16 | 13 | | 73 |
| Fish | Black Bullhead (*Ameiurus melas*) | Ictaluridae | 1996 | 1.00 | 12 | 6 | | 50 |
| Fish | Black Bullhead (*Ameiurus melas*) | Ictaluridae | 1999 | 5.50 | 10 | 6 | | 40 |
| Fish | Black Bullhead (*Ameiurus melas*) | Ictaluridae | 2000 | 2.33 | 16 | 6 | | 64 |
| Fish | Black Bullhead (*Ameiurus melas*) | Ictaluridae | 2001 | 0.50 | 23 | 6 | | 92 |
| Fish | Black Bullhead (*Ameiurus melas*) | Ictaluridae | 2002 | 0.67 | 22 | 6 | | 88 |
| Fish | Black Bullhead (*Ameiurus melas*) | Ictaluridae | 2007 | 0.33 | 22 | 6 | | 100 |
| Fish | Black Crappie (*Pomoxis nigromaculatus*) | Centrarchidae | 1995 | 11.39 | 5 | 17 | | 22 |
| Fish | Black Crappie (*Pomoxis nigromaculatus*) | Centrarchidae | 1996 | 1.81 | 9 | 17 | | 38 |
| Fish | Black Crappie (*Pomoxis nigromaculatus*) | Centrarchidae | 1997 | 6.33 | 7 | 17 | | 35 |
| Fish | Black Crappie (*Pomoxis nigromaculatus*) | Centrarchidae | 1998 | 6.33 | 6 | 17 | | 25 |
| Fish | Black Crappie (*Pomoxis nigromaculatus*) | Centrarchidae | 1999 | 1.87 | 15 | 17 | | 60 |
| Fish | Black Crappie (*Pomoxis nigromaculatus*) | Centrarchidae | 2000 | 0.75 | 20 | 17 | | 80 |
| Fish | Black Crappie (*Pomoxis nigromaculatus*) | Centrarchidae | 2001 | 3.25 | 13 | 17 | | 52 |
| Fish | Black Crappie (*Pomoxis nigromaculatus*) | Centrarchidae | 2002 | 10.22 | 7 | 17 | | 28 |
| Fish | Black Crappie (*Pomoxis nigromaculatus*) | Centrarchidae | 2003 | 4.50 | 10 | 17 | | 40 |
| Fish | Black Crappie (*Pomoxis nigromaculatus*) | Centrarchidae | 2004 | 24.33 | 6 | 17 | | 24 |
| Fish | Black Crappie (*Pomoxis nigromaculatus*) | Centrarchidae | 2005 | 15.58 | 6 | 17 | | 21 |
| Fish | Black Crappie (*Pomoxis nigromaculatus*) | Centrarchidae | 2006 | 1.30 | 14 | 17 | | 64 |
| Fish | Black Crappie (*Pomoxis nigromaculatus*) | Centrarchidae | 2007 | 1.00 | 17 | 17 | | 77 |
| Fish | Black Crappie (*Pomoxis nigromaculatus*) | Centrarchidae | 2008 | 2.50 | 14 | 17 | | 56 |
| Fish | Black Crappie (*Pomoxis nigromaculatus*) | Centrarchidae | 2009 | 19.83 | 4 | 17 | | 18 |
| Fish | Black Crappie (*Pomoxis nigromaculatus*) | Centrarchidae | 2010 | 18.33 | 4 | 17 | | 18 |
| Fish | Black Crappie (*Pomoxis nigromaculatus*) | Centrarchidae | 2011 | 12.75 | 5 | 17 | | 22 |
| Fish | Blacknose Shiner (*Notropis heterolepis*) | Cyprinidae | 2003 | 0.08 | 25 | 2 | | 100 |
| Fish | Blacknose Shiner (*Notropis heterolepis*) | Cyprinidae | 2008 | 1.00 | 17 | 2 | | 68 |
| Fish | Bluegill (*Lepomis macrochirus*) | Centrarchidae | 1995 | 42.53 | 3 | 17 | | 13 |
| Fish | Bluegill (*Lepomis macrochirus*) | Centrarchidae | 1996 | 22.78 | 2 | 17 | | 8 |
| Fish | Bluegill (*Lepomis macrochirus*) | Centrarchidae | 1997 | 56.83 | 2 | 17 | | 10 |
| Fish | Bluegill (*Lepomis macrochirus*) | Centrarchidae | 1998 | 24.67 | 2 | 17 | | 8 |
| Fish | Bluegill (*Lepomis macrochirus*) | Centrarchidae | 1999 | 17.26 | 6 | 17 | | 24 |
| Fish | Bluegill (*Lepomis macrochirus*) | Centrarchidae | 2000 | 82.33 | 2 | 17 | | 8 |
| Fish | Bluegill (*Lepomis macrochirus*) | Centrarchidae | 2001 | 54.84 | 3 | 17 | | 12 |
| Fish | Bluegill (*Lepomis macrochirus*) | Centrarchidae | 2002 | 94.97 | 2 | 17 | | 8 |
| Fish | Bluegill (*Lepomis macrochirus*) | Centrarchidae | 2003 | 25.64 | 2 | 17 | | 8 |
| Fish | Bluegill (*Lepomis macrochirus*) | Centrarchidae | 2004 | 36.42 | 2 | 17 | | 8 |
| Fish | Bluegill (*Lepomis macrochirus*) | Centrarchidae | 2005 | 117.75 | 1 | 17 | | 4 |
| Fish | Bluegill (*Lepomis macrochirus*) | Centrarchidae | 2006 | 91.19 | 1 | 17 | | 5 |
| Fish | Bluegill (*Lepomis macrochirus*) | Centrarchidae | 2007 | 139.67 | 1 | 17 | | 5 |
| Fish | Bluegill (*Lepomis macrochirus*) | Centrarchidae | 2008 | 65.35 | 2 | 17 | | 8 |
| Fish | Bluegill (*Lepomis macrochirus*) | Centrarchidae | 2009 | 190.67 | 1 | 17 | | 5 |
| Fish | Bluegill (*Lepomis macrochirus*) | Centrarchidae | 2010 | 40.25 | 1 | 17 | | 5 |
| Fish | Bluegill (*Lepomis macrochirus*) | Centrarchidae | 2011 | 34.83 | 3 | 17 | | 13 |
| Fish | Bluntnose Minnow (*Pimephales notatus*) | Cyprinidae | 1995 | 0.22 | 21 | 16 | | 91 |
| Fish | Bluntnose Minnow (*Pimephales notatus*) | Cyprinidae | 1996 | 0.56 | 13 | 16 | | 54 |
| Fish | Bluntnose Minnow (*Pimephales notatus*) | Cyprinidae | 1998 | 0.17 | 24 | 16 | | 100 |
| Fish | Bluntnose Minnow (*Pimephales notatus*) | Cyprinidae | 1999 | 0.42 | 24 | 16 | | 96 |
| Fish | Bluntnose Minnow (*Pimephales notatus*) | Cyprinidae | 2000 | 9.42 | 6 | 16 | | 24 |
| Fish | Bluntnose Minnow (*Pimephales notatus*) | Cyprinidae | 2001 | 1.75 | 15 | 16 | | 60 |
| Fish | Bluntnose Minnow (*Pimephales notatus*) | Cyprinidae | 2002 | 12.22 | 6 | 16 | | 24 |
| Fish | Bluntnose Minnow (*Pimephales notatus*) | Cyprinidae | 2003 | 17.00 | 4 | 16 | | 16 |
| Fish | Bluntnose Minnow (*Pimephales notatus*) | Cyprinidae | 2004 | 28.50 | 4 | 16 | | 16 |
| Fish | Bluntnose Minnow (*Pimephales notatus*) | Cyprinidae | 2005 | 4.75 | 12 | 16 | | 43 |
| Fish | Bluntnose Minnow (*Pimephales notatus*) | Cyprinidae | 2006 | 1.17 | 15 | 16 | | 68 |
| Fish | Bluntnose Minnow (*Pimephales notatus*) | Cyprinidae | 2007 | 7.17 | 7 | 16 | | 32 |
| Fish | Bluntnose Minnow (*Pimephales notatus*) | Cyprinidae | 2008 | 0.67 | 22 | 16 | | 88 |
| Fish | Bluntnose Minnow (*Pimephales notatus*) | Cyprinidae | 2009 | 16.83 | 5 | 16 | | 23 |
| Fish | Bluntnose Minnow (*Pimephales notatus*) | Cyprinidae | 2010 | 0.67 | 18 | 16 | | 82 |
| Fish | Bluntnose Minnow (*Pimephales notatus*) | Cyprinidae | 2011 | 7.58 | 7 | 16 | | 30 |
| Fish | Bowfin (*Amia calva*) | Amiidae | 1998 | 0.33 | 21 | 7 | | 88 |
| Fish | Bowfin (*Amia calva*) | Amiidae | 2000 | 0.83 | 19 | 7 | | 76 |
| Fish | Bowfin (*Amia calva*) | Amiidae | 2001 | 0.33 | 24 | 7 | | 96 |
| Fish | Bowfin (*Amia calva*) | Amiidae | 2003 | 0.33 | 23 | 7 | | 92 |
| Fish | Bowfin (*Amia calva*) | Amiidae | 2004 | 0.33 | 22 | 7 | | 88 |
| Fish | Bowfin (*Amia calva*) | Amiidae | 2005 | 0.33 | 24 | 7 | | 86 |
| Fish | Bowfin (*Amia calva*) | Amiidae | 2011 | 0.17 | 22 | 7 | | 96 |
| Fish | Brook Silverside (*Labidesthes sicculus*) | Atherinopsidae | 1995 | 69.11 | 1 | 17 | | 4 |
| Fish | Brook Silverside (*Labidesthes sicculus*) | Atherinopsidae | 1996 | 20.08 | 3 | 17 | | 13 |
| Fish | Brook Silverside (*Labidesthes sicculus*) | Atherinopsidae | 1997 | 10.70 | 4 | 17 | | 20 |
| Fish | Brook Silverside (*Labidesthes sicculus*) | Atherinopsidae | 1998 | 7.42 | 5 | 17 | | 21 |
| Fish | Brook Silverside (*Labidesthes sicculus*) | Atherinopsidae | 1999 | 78.17 | 2 | 17 | | 8 |
| Fish | Brook Silverside (*Labidesthes sicculus*) | Atherinopsidae | 2000 | 73.83 | 3 | 17 | | 12 |
| Fish | Brook Silverside (*Labidesthes sicculus*) | Atherinopsidae | 2001 | 69.75 | 1 | 17 | | 4 |
| Fish | Brook Silverside (*Labidesthes sicculus*) | Atherinopsidae | 2002 | 139.23 | 1 | 17 | | 4 |
| Fish | Brook Silverside (*Labidesthes sicculus*) | Atherinopsidae | 2003 | 101.50 | 1 | 17 | | 4 |
| Fish | Brook Silverside (*Labidesthes sicculus*) | Atherinopsidae | 2004 | 33.58 | 3 | 17 | | 12 |
| Fish | Brook Silverside (*Labidesthes sicculus*) | Atherinopsidae | 2005 | 15.58 | 5 | 17 | | 18 |
| Fish | Brook Silverside (*Labidesthes sicculus*) | Atherinopsidae | 2006 | 78.07 | 3 | 17 | | 14 |
| Fish | Brook Silverside (*Labidesthes sicculus*) | Atherinopsidae | 2007 | 30.00 | 3 | 17 | | 14 |
| Fish | Brook Silverside (*Labidesthes sicculus*) | Atherinopsidae | 2008 | 78.50 | 1 | 17 | | 4 |
| Fish | Brook Silverside (*Labidesthes sicculus*) | Atherinopsidae | 2009 | 49.92 | 2 | 17 | | 9 |
| Fish | Brook Silverside (*Labidesthes sicculus*) | Atherinopsidae | 2010 | 38.25 | 2 | 17 | | 9 |
| Fish | Brook Silverside (*Labidesthes sicculus*) | Atherinopsidae | 2011 | 435.08 | 1 | 17 | | 4 |
| Fish | Brown Bullhead (*Ameiurus nebulosus*) | Ictaluridae | 1995 | 0.25 | 20 | 11 | | 87 |
| Fish | Brown Bullhead (*Ameiurus nebulosus*) | Ictaluridae | 1996 | 0.25 | 18 | 11 | | 75 |
| Fish | Brown Bullhead (*Ameiurus nebulosus*) | Ictaluridae | 1997 | 2.50 | 9 | 11 | | 45 |
| Fish | Brown Bullhead (*Ameiurus nebulosus*) | Ictaluridae | 1998 | 0.33 | 19 | 11 | | 79 |
| Fish | Brown Bullhead (*Ameiurus nebulosus*) | Ictaluridae | 1999 | 0.83 | 22 | 11 | | 88 |
| Fish | Brown Bullhead (*Ameiurus nebulosus*) | Ictaluridae | 2000 | 2.17 | 17 | 11 | | 68 |
| Fish | Brown Bullhead (*Ameiurus nebulosus*) | Ictaluridae | 2002 | 0.67 | 21 | 11 | | 84 |
| Fish | Brown Bullhead (*Ameiurus nebulosus*) | Ictaluridae | 2005 | 0.58 | 22 | 11 | | 79 |
| Fish | Brown Bullhead (*Ameiurus nebulosus*) | Ictaluridae | 2006 | 0.50 | 20 | 11 | | 91 |
| Fish | Brown Bullhead (*Ameiurus nebulosus*) | Ictaluridae | 2008 | 0.83 | 20 | 11 | | 80 |
| Fish | Brown Bullhead (*Ameiurus nebulosus*) | Ictaluridae | 2009 | 0.33 | 19 | 11 | | 86 |
| Fish | Channel Catfish (*Ictalurus punctatus*) | Ictaluridae | 1996 | 0.06 | 23 | 15 | | 96 |
| Fish | Channel Catfish (*Ictalurus punctatus*) | Ictaluridae | 1998 | 0.75 | 16 | 15 | | 67 |
| Fish | Channel Catfish (*Ictalurus punctatus*) | Ictaluridae | 1999 | 1.50 | 18 | 15 | | 72 |
| Fish | Channel Catfish (*Ictalurus punctatus*) | Ictaluridae | 2000 | 5.51 | 11 | 15 | | 44 |
| Fish | Channel Catfish (*Ictalurus punctatus*) | Ictaluridae | 2001 | 2.83 | 14 | 15 | | 56 |
| Fish | Channel Catfish (*Ictalurus punctatus*) | Ictaluridae | 2002 | 2.66 | 16 | 15 | | 64 |
| Fish | Channel Catfish (*Ictalurus punctatus*) | Ictaluridae | 2003 | 2.67 | 13 | 15 | | 52 |
| Fish | Channel Catfish (*Ictalurus punctatus*) | Ictaluridae | 2004 | 4.17 | 14 | 15 | | 56 |
| Fish | Channel Catfish (*Ictalurus punctatus*) | Ictaluridae | 2005 | 1.50 | 17 | 15 | | 61 |
| Fish | Channel Catfish (*Ictalurus punctatus*) | Ictaluridae | 2006 | 1.50 | 12 | 15 | | 55 |
| Fish | Channel Catfish (*Ictalurus punctatus*) | Ictaluridae | 2007 | 1.00 | 16 | 15 | | 73 |
| Fish | Channel Catfish (*Ictalurus punctatus*) | Ictaluridae | 2008 | 3.83 | 12 | 15 | | 48 |
| Fish | Channel Catfish (*Ictalurus punctatus*) | Ictaluridae | 2009 | 2.50 | 13 | 15 | | 59 |
| Fish | Channel Catfish (*Ictalurus punctatus*) | Ictaluridae | 2010 | 7.17 | 8 | 15 | | 36 |
| Fish | Channel Catfish (*Ictalurus punctatus*) | Ictaluridae | 2011 | 1.34 | 18 | 15 | | 78 |
| Fish | Cisco (*Coregonus artedi*) | Salmonidae | 1995 | 0.66 | 16 | 13 | | 70 |
| Fish | Cisco (*Coregonus artedi*) | Salmonidae | 1997 | 0.45 | 14 | 13 | | 70 |
| Fish | Cisco (*Coregonus artedi*) | Salmonidae | 1999 | 0.79 | 23 | 13 | | 92 |
| Fish | Cisco (*Coregonus artedi*) | Salmonidae | 2001 | 0.52 | 19 | 13 | | 76 |
| Fish | Cisco (*Coregonus artedi*) | Salmonidae | 2002 | 1.00 | 19 | 13 | | 76 |
| Fish | Cisco (*Coregonus artedi*) | Salmonidae | 2003 | 0.51 | 22 | 13 | | 88 |
| Fish | Cisco (*Coregonus artedi*) | Salmonidae | 2004 | 3.00 | 15 | 13 | | 60 |
| Fish | Cisco (*Coregonus artedi*) | Salmonidae | 2005 | 5.73 | 11 | 13 | | 39 |
| Fish | Cisco (*Coregonus artedi*) | Salmonidae | 2006 | 0.96 | 18 | 13 | | 82 |
| Fish | Cisco (*Coregonus artedi*) | Salmonidae | 2007 | 0.67 | 19 | 13 | | 86 |
| Fish | Cisco (*Coregonus artedi*) | Salmonidae | 2008 | 4.65 | 11 | 13 | | 44 |
| Fish | Cisco (*Coregonus artedi*) | Salmonidae | 2009 | 2.45 | 14 | 13 | | 64 |
| Fish | Cisco (*Coregonus artedi*) | Salmonidae | 2010 | 1.92 | 14 | 13 | | 64 |
| Fish | Common Carp (*Cyprinus carpio*) | Cyprinidae | 1995 | 2.36 | 8 | 17 | | 35 |
| Fish | Common Carp (*Cyprinus carpio*) | Cyprinidae | 1996 | 5.56 | 5 | 17 | | 21 |
| Fish | Common Carp (*Cyprinus carpio*) | Cyprinidae | 1997 | 6.83 | 6 | 17 | | 30 |
| Fish | Common Carp (*Cyprinus carpio*) | Cyprinidae | 1998 | 5.50 | 7 | 17 | | 29 |
| Fish | Common Carp (*Cyprinus carpio*) | Cyprinidae | 1999 | 5.83 | 8 | 17 | | 32 |
| Fish | Common Carp (*Cyprinus carpio*) | Cyprinidae | 2000 | 5.83 | 10 | 17 | | 40 |
| Fish | Common Carp (*Cyprinus carpio*) | Cyprinidae | 2001 | 9.58 | 7 | 17 | | 28 |
| Fish | Common Carp (*Cyprinus carpio*) | Cyprinidae | 2002 | 5.67 | 12 | 17 | | 48 |
| Fish | Common Carp (*Cyprinus carpio*) | Cyprinidae | 2003 | 2.67 | 14 | 17 | | 56 |
| Fish | Common Carp (*Cyprinus carpio*) | Cyprinidae | 2004 | 5.33 | 13 | 17 | | 52 |
| Fish | Common Carp (*Cyprinus carpio*) | Cyprinidae | 2005 | 7.00 | 10 | 17 | | 36 |
| Fish | Common Carp (*Cyprinus carpio*) | Cyprinidae | 2006 | 4.83 | 7 | 17 | | 32 |
| Fish | Common Carp (*Cyprinus carpio*) | Cyprinidae | 2007 | 3.67 | 10 | 17 | | 45 |
| Fish | Common Carp (*Cyprinus carpio*) | Cyprinidae | 2008 | 8.00 | 8 | 17 | | 32 |
| Fish | Common Carp (*Cyprinus carpio*) | Cyprinidae | 2009 | 3.00 | 11 | 17 | | 50 |
| Fish | Common Carp (*Cyprinus carpio*) | Cyprinidae | 2010 | 6.00 | 9 | 17 | | 41 |
| Fish | Common Carp (*Cyprinus carpio*) | Cyprinidae | 2011 | 7.00 | 8 | 17 | | 35 |
| Fish | Emerald Shiner (*Notropis atherinoides*) | Cyprinidae | 2000 | 0.25 | 24 | 2 | | 96 |
| Fish | Emerald Shiner (*Notropis atherinoides*) | Cyprinidae | 2011 | 0.08 | 23 | 2 | | 100 |
| Fish | Freshwater Drum (*Aplodinotus grunniens*) | Sciaenidae | 1995 | 9.49 | 6 | 17 | | 26 |
| Fish | Freshwater Drum (*Aplodinotus grunniens*) | Sciaenidae | 1996 | 3.40 | 7 | 17 | | 29 |
| Fish | Freshwater Drum (*Aplodinotus grunniens*) | Sciaenidae | 1997 | 10.07 | 5 | 17 | | 25 |
| Fish | Freshwater Drum (*Aplodinotus grunniens*) | Sciaenidae | 1998 | 13.96 | 4 | 17 | | 17 |
| Fish | Freshwater Drum (*Aplodinotus grunniens*) | Sciaenidae | 1999 | 22.06 | 5 | 17 | | 20 |
| Fish | Freshwater Drum (*Aplodinotus grunniens*) | Sciaenidae | 2000 | 26.85 | 5 | 17 | | 20 |
| Fish | Freshwater Drum (*Aplodinotus grunniens*) | Sciaenidae | 2001 | 10.50 | 6 | 17 | | 24 |
| Fish | Freshwater Drum (*Aplodinotus grunniens*) | Sciaenidae | 2002 | 15.11 | 5 | 17 | | 20 |
| Fish | Freshwater Drum (*Aplodinotus grunniens*) | Sciaenidae | 2003 | 20.87 | 3 | 17 | | 12 |
| Fish | Freshwater Drum (*Aplodinotus grunniens*) | Sciaenidae | 2004 | 26.38 | 5 | 17 | | 20 |
| Fish | Freshwater Drum (*Aplodinotus grunniens*) | Sciaenidae | 2005 | 30.28 | 3 | 17 | | 11 |
| Fish | Freshwater Drum (*Aplodinotus grunniens*) | Sciaenidae | 2006 | 7.74 | 5 | 17 | | 23 |
| Fish | Freshwater Drum (*Aplodinotus grunniens*) | Sciaenidae | 2007 | 6.67 | 8 | 17 | | 36 |
| Fish | Freshwater Drum (*Aplodinotus grunniens*) | Sciaenidae | 2008 | 12.76 | 5 | 17 | | 20 |
| Fish | Freshwater Drum (*Aplodinotus grunniens*) | Sciaenidae | 2009 | 6.77 | 7 | 17 | | 32 |
| Fish | Freshwater Drum (*Aplodinotus grunniens*) | Sciaenidae | 2010 | 7.31 | 7 | 17 | | 32 |
| Fish | Freshwater Drum (*Aplodinotus grunniens*) | Sciaenidae | 2011 | 8.68 | 6 | 17 | | 26 |
| Fish | Golden Shiner (*Notemigonus crysoleucas*) | Cyprinidae | 1995 | 0.31 | 19 | 9 | | 83 |
| Fish | Golden Shiner (*Notemigonus crysoleucas*) | Cyprinidae | 1996 | 0.06 | 24 | 9 | | 100 |
| Fish | Golden Shiner (*Notemigonus crysoleucas*) | Cyprinidae | 1998 | 0.42 | 18 | 9 | | 75 |
| Fish | Golden Shiner (*Notemigonus crysoleucas*) | Cyprinidae | 2003 | 0.08 | 24 | 9 | | 96 |
| Fish | Golden Shiner (*Notemigonus crysoleucas*) | Cyprinidae | 2005 | 0.08 | 28 | 9 | | 100 |
| Fish | Golden Shiner (*Notemigonus crysoleucas*) | Cyprinidae | 2007 | 0.33 | 20 | 9 | | 91 |
| Fish | Golden Shiner (*Notemigonus crysoleucas*) | Cyprinidae | 2009 | 0.08 | 22 | 9 | | 100 |
| Fish | Golden Shiner (*Notemigonus crysoleucas*) | Cyprinidae | 2010 | 0.17 | 21 | 9 | | 95 |
| Fish | Golden Shiner (*Notemigonus crysoleucas*) | Cyprinidae | 2011 | 1.67 | 17 | 9 | | 74 |
| Fish | Green Sunfish (*Lepomis cyanellus*) | Centrarchidae | 1995 | 0.06 | 23 | 8 | | 100 |
| Fish | Green Sunfish (*Lepomis cyanellus*) | Centrarchidae | 1998 | 0.17 | 23 | 8 | | 96 |
| Fish | Green Sunfish (*Lepomis cyanellus*) | Centrarchidae | 1999 | 1.25 | 20 | 8 | | 80 |
| Fish | Green Sunfish (*Lepomis cyanellus*) | Centrarchidae | 2000 | 0.25 | 25 | 8 | | 100 |
| Fish | Green Sunfish (*Lepomis cyanellus*) | Centrarchidae | 2001 | 0.50 | 20 | 8 | | 80 |
| Fish | Green Sunfish (*Lepomis cyanellus*) | Centrarchidae | 2002 | 0.83 | 20 | 8 | | 80 |
| Fish | Green Sunfish (*Lepomis cyanellus*) | Centrarchidae | 2005 | 0.17 | 25 | 8 | | 89 |
| Fish | Green Sunfish (*Lepomis cyanellus*) | Centrarchidae | 2008 | 0.17 | 24 | 8 | | 96 |
| Fish | Iowa Darter (*Etheostoma exile*) | Percidae | 1996 | 0.17 | 20 | 5 | | 83 |
| Fish | Iowa Darter (*Etheostoma exile*) | Percidae | 2004 | 0.50 | 21 | 5 | | 84 |
| Fish | Iowa Darter (*Etheostoma exile*) | Percidae | 2006 | 0.17 | 21 | 5 | | 95 |
| Fish | Iowa Darter (*Etheostoma exile*) | Percidae | 2008 | 0.08 | 25 | 5 | | 100 |
| Fish | Iowa Darter (*Etheostoma exile*) | Percidae | 2011 | 0.33 | 20 | 5 | | 87 |
| Fish | Johnny Darter (*Etheostoma nigrum*) | Percidae | 1997 | 0.33 | 17 | 3 | | 85 |
| Fish | Johnny Darter (*Etheostoma nigrum*) | Percidae | 2001 | 0.17 | 25 | 3 | | 100 |
| Fish | Johnny Darter (*Etheostoma nigrum*) | Percidae | 2005 | 0.17 | 27 | 3 | | 96 |
| Fish | Largemouth Bass (*Micropterus salmoides*) | Centrarchidae | 1995 | 0.36 | 18 | 16 | | 78 |
| Fish | Largemouth Bass (*Micropterus salmoides*) | Centrarchidae | 1996 | 0.06 | 22 | 16 | | 92 |
| Fish | Largemouth Bass (*Micropterus salmoides*) | Centrarchidae | 1997 | 1.33 | 12 | 16 | | 60 |
| Fish | Largemouth Bass (*Micropterus salmoides*) | Centrarchidae | 1998 | 1.17 | 11 | 16 | | 46 |
| Fish | Largemouth Bass (*Micropterus salmoides*) | Centrarchidae | 1999 | 1.33 | 19 | 16 | | 76 |
| Fish | Largemouth Bass (*Micropterus salmoides*) | Centrarchidae | 2000 | 6.17 | 9 | 16 | | 36 |
| Fish | Largemouth Bass (*Micropterus salmoides*) | Centrarchidae | 2001 | 3.67 | 12 | 16 | | 48 |
| Fish | Largemouth Bass (*Micropterus salmoides*) | Centrarchidae | 2002 | 3.25 | 14 | 16 | | 56 |
| Fish | Largemouth Bass (*Micropterus salmoides*) | Centrarchidae | 2003 | 1.75 | 16 | 16 | | 64 |
| Fish | Largemouth Bass (*Micropterus salmoides*) | Centrarchidae | 2004 | 5.92 | 10 | 16 | | 40 |
| Fish | Largemouth Bass (*Micropterus salmoides*) | Centrarchidae | 2005 | 1.25 | 19 | 16 | | 68 |
| Fish | Largemouth Bass (*Micropterus salmoides*) | Centrarchidae | 2007 | 1.33 | 15 | 16 | | 68 |
| Fish | Largemouth Bass (*Micropterus salmoides*) | Centrarchidae | 2008 | 0.83 | 19 | 16 | | 76 |
| Fish | Largemouth Bass (*Micropterus salmoides*) | Centrarchidae | 2009 | 1.17 | 17 | 16 | | 77 |
| Fish | Largemouth Bass (*Micropterus salmoides*) | Centrarchidae | 2010 | 0.92 | 17 | 16 | | 77 |
| Fish | Largemouth Bass (*Micropterus salmoides*) | Centrarchidae | 2011 | 6.25 | 10 | 16 | | 43 |
| Fish | Logperch (*Percina caprodes*) | Percidae | 1996 | 0.25 | 17 | 14 | | 71 |
| Fish | Logperch (*Percina caprodes*) | Percidae | 1998 | 1.00 | 13 | 14 | | 54 |
| Fish | Logperch (*Percina caprodes*) | Percidae | 1999 | 1.67 | 17 | 14 | | 68 |
| Fish | Logperch (*Percina caprodes*) | Percidae | 2001 | 1.33 | 17 | 14 | | 68 |
| Fish | Logperch (*Percina caprodes*) | Percidae | 2002 | 1.83 | 17 | 14 | | 68 |
| Fish | Logperch (*Percina caprodes*) | Percidae | 2003 | 3.50 | 11 | 14 | | 44 |
| Fish | Logperch (*Percina caprodes*) | Percidae | 2004 | 5.92 | 11 | 14 | | 44 |
| Fish | Logperch (*Percina caprodes*) | Percidae | 2005 | 3.08 | 13 | 14 | | 46 |
| Fish | Logperch (*Percina caprodes*) | Percidae | 2006 | 2.77 | 8 | 14 | | 36 |
| Fish | Logperch (*Percina caprodes*) | Percidae | 2007 | 3.33 | 11 | 14 | | 50 |
| Fish | Logperch (*Percina caprodes*) | Percidae | 2008 | 8.17 | 7 | 14 | | 28 |
| Fish | Logperch (*Percina caprodes*) | Percidae | 2009 | 6.17 | 8 | 14 | | 36 |
| Fish | Logperch (*Percina caprodes*) | Percidae | 2010 | 3.42 | 10 | 14 | | 45 |
| Fish | Logperch (*Percina caprodes*) | Percidae | 2011 | 3.50 | 13 | 14 | | 57 |
| Fish | Longnose Gar (*Lepisosteus osseus*) | Lepisosteidae | 1995 | 1.00 | 12 | 13 | | 52 |
| Fish | Longnose Gar (*Lepisosteus osseus*) | Lepisosteidae | 1996 | 1.07 | 11 | 13 | | 46 |
| Fish | Longnose Gar (*Lepisosteus osseus*) | Lepisosteidae | 1998 | 1.00 | 12 | 13 | | 50 |
| Fish | Longnose Gar (*Lepisosteus osseus*) | Lepisosteidae | 1999 | 1.00 | 21 | 13 | | 84 |
| Fish | Longnose Gar (*Lepisosteus osseus*) | Lepisosteidae | 2000 | 0.33 | 23 | 13 | | 92 |
| Fish | Longnose Gar (*Lepisosteus osseus*) | Lepisosteidae | 2001 | 0.92 | 18 | 13 | | 72 |
| Fish | Longnose Gar (*Lepisosteus osseus*) | Lepisosteidae | 2002 | 1.33 | 18 | 13 | | 72 |
| Fish | Longnose Gar (*Lepisosteus osseus*) | Lepisosteidae | 2003 | 1.17 | 18 | 13 | | 72 |
| Fish | Longnose Gar (*Lepisosteus osseus*) | Lepisosteidae | 2004 | 1.00 | 18 | 13 | | 72 |
| Fish | Longnose Gar (*Lepisosteus osseus*) | Lepisosteidae | 2005 | 0.83 | 20 | 13 | | 71 |
| Fish | Longnose Gar (*Lepisosteus osseus*) | Lepisosteidae | 2006 | 2.47 | 10 | 13 | | 45 |
| Fish | Longnose Gar (*Lepisosteus osseus*) | Lepisosteidae | 2007 | 1.00 | 18 | 13 | | 82 |
| Fish | Longnose Gar (*Lepisosteus osseus*) | Lepisosteidae | 2008 | 0.50 | 23 | 13 | | 92 |
| Fish | Mottled Sculpin (*Cottus bairdii*) | Cottidae | 2004 | 0.08 | 24 | 3 | | 96 |
| Fish | Mottled Sculpin (*Cottus bairdii*) | Cottidae | 2005 | 0.42 | 23 | 3 | | 82 |
| Fish | Mottled Sculpin (*Cottus bairdii*) | Cottidae | 2009 | 0.17 | 20 | 3 | | 91 |
| Fish | Northern Pike (*Esox lucius*) | Esocidae | 1995 | 0.75 | 14 | 13 | | 61 |
| Fish | Northern Pike (*Esox lucius*) | Esocidae | 1996 | 0.53 | 14 | 13 | | 58 |
| Fish | Northern Pike (*Esox lucius*) | Esocidae | 1998 | 0.67 | 17 | 13 | | 71 |
| Fish | Northern Pike (*Esox lucius*) | Esocidae | 2000 | 0.33 | 22 | 13 | | 88 |
| Fish | Northern Pike (*Esox lucius*) | Esocidae | 2002 | 0.20 | 25 | 13 | | 100 |
| Fish | Northern Pike (*Esox lucius*) | Esocidae | 2003 | 1.17 | 20 | 13 | | 80 |
| Fish | Northern Pike (*Esox lucius*) | Esocidae | 2004 | 1.17 | 17 | 13 | | 68 |
| Fish | Northern Pike (*Esox lucius*) | Esocidae | 2006 | 1.03 | 16 | 13 | | 73 |
| Fish | Northern Pike (*Esox lucius*) | Esocidae | 2007 | 1.50 | 12 | 13 | | 55 |
| Fish | Northern Pike (*Esox lucius*) | Esocidae | 2008 | 1.50 | 15 | 13 | | 60 |
| Fish | Northern Pike (*Esox lucius*) | Esocidae | 2009 | 0.33 | 18 | 13 | | 82 |
| Fish | Northern Pike (*Esox lucius*) | Esocidae | 2010 | 0.33 | 19 | 13 | | 86 |
| Fish | Northern Pike (*Esox lucius*) | Esocidae | 2011 | 1.83 | 15 | 13 | | 65 |
| Fish | Pumpkinseed (*Lepomis gibbosus*) | Centrarchidae | 1995 | 0.67 | 15 | 15 | | 65 |
| Fish | Pumpkinseed (*Lepomis gibbosus*) | Centrarchidae | 1996 | 0.17 | 19 | 15 | | 79 |
| Fish | Pumpkinseed (*Lepomis gibbosus*) | Centrarchidae | 1997 | 0.33 | 19 | 15 | | 95 |
| Fish | Pumpkinseed (*Lepomis gibbosus*) | Centrarchidae | 1999 | 3.25 | 12 | 15 | | 48 |
| Fish | Pumpkinseed (*Lepomis gibbosus*) | Centrarchidae | 2000 | 4.00 | 13 | 15 | | 52 |
| Fish | Pumpkinseed (*Lepomis gibbosus*) | Centrarchidae | 2001 | 4.42 | 11 | 15 | | 44 |
| Fish | Pumpkinseed (*Lepomis gibbosus*) | Centrarchidae | 2002 | 7.75 | 9 | 15 | | 36 |
| Fish | Pumpkinseed (*Lepomis gibbosus*) | Centrarchidae | 2003 | 1.08 | 21 | 15 | | 84 |
| Fish | Pumpkinseed (*Lepomis gibbosus*) | Centrarchidae | 2004 | 1.00 | 19 | 15 | | 76 |
| Fish | Pumpkinseed (*Lepomis gibbosus*) | Centrarchidae | 2005 | 2.58 | 15 | 15 | | 54 |
| Fish | Pumpkinseed (*Lepomis gibbosus*) | Centrarchidae | 2006 | 2.43 | 11 | 15 | | 50 |
| Fish | Pumpkinseed (*Lepomis gibbosus*) | Centrarchidae | 2007 | 1.33 | 14 | 15 | | 64 |
| Fish | Pumpkinseed (*Lepomis gibbosus*) | Centrarchidae | 2008 | 0.83 | 18 | 15 | | 72 |
| Fish | Pumpkinseed (*Lepomis gibbosus*) | Centrarchidae | 2010 | 0.17 | 20 | 15 | | 91 |
| Fish | Pumpkinseed (*Lepomis gibbosus*) | Centrarchidae | 2011 | 0.42 | 19 | 15 | | 83 |
| Fish | Rock Bass (*Ambloplites rupestris*) | Centrarchidae | 1995 | 2.00 | 9 | 17 | | 39 |
| Fish | Rock Bass (*Ambloplites rupestris*) | Centrarchidae | 1996 | 4.75 | 6 | 17 | | 25 |
| Fish | Rock Bass (*Ambloplites rupestris*) | Centrarchidae | 1997 | 1.33 | 11 | 17 | | 55 |
| Fish | Rock Bass (*Ambloplites rupestris*) | Centrarchidae | 1998 | 4.00 | 8 | 17 | | 33 |
| Fish | Rock Bass (*Ambloplites rupestris*) | Centrarchidae | 1999 | 6.75 | 7 | 17 | | 28 |
| Fish | Rock Bass (*Ambloplites rupestris*) | Centrarchidae | 2000 | 7.00 | 8 | 17 | | 32 |
| Fish | Rock Bass (*Ambloplites rupestris*) | Centrarchidae | 2001 | 5.75 | 9 | 17 | | 36 |
| Fish | Rock Bass (*Ambloplites rupestris*) | Centrarchidae | 2002 | 5.82 | 11 | 17 | | 44 |
| Fish | Rock Bass (*Ambloplites rupestris*) | Centrarchidae | 2003 | 3.42 | 12 | 17 | | 48 |
| Fish | Rock Bass (*Ambloplites rupestris*) | Centrarchidae | 2004 | 9.00 | 9 | 17 | | 36 |
| Fish | Rock Bass (*Ambloplites rupestris*) | Centrarchidae | 2005 | 7.42 | 8 | 17 | | 29 |
| Fish | Rock Bass (*Ambloplites rupestris*) | Centrarchidae | 2006 | 1.43 | 13 | 17 | | 59 |
| Fish | Rock Bass (*Ambloplites rupestris*) | Centrarchidae | 2007 | 8.00 | 6 | 17 | | 27 |
| Fish | Rock Bass (*Ambloplites rupestris*) | Centrarchidae | 2008 | 4.83 | 10 | 17 | | 40 |
| Fish | Rock Bass (*Ambloplites rupestris*) | Centrarchidae | 2009 | 2.33 | 15 | 17 | | 68 |
| Fish | Rock Bass (*Ambloplites rupestris*) | Centrarchidae | 2010 | 3.00 | 11 | 17 | | 50 |
| Fish | Rock Bass (*Ambloplites rupestris*) | Centrarchidae | 2011 | 6.92 | 9 | 17 | | 39 |
| Fish | Shorthead Redhorse (*Moxostoma macrolepidotum*) | Catostomidae | 1995 | 1.50 | 10 | 1 | | 43 |
| Fish | Silver Redhorse (*Moxostoma anisurum*) | Catostomidae | 1995 | 0.50 | 17 | 1 | | 74 |
| Fish | Smallmouth Bass (*Micropterus dolomieu*) | Centrarchidae | 1995 | 3.25 | 7 | 17 | | 30 |
| Fish | Smallmouth Bass (*Micropterus dolomieu*) | Centrarchidae | 1996 | 3.11 | 8 | 17 | | 33 |
| Fish | Smallmouth Bass (*Micropterus dolomieu*) | Centrarchidae | 1997 | 3.00 | 8 | 17 | | 40 |
| Fish | Smallmouth Bass (*Micropterus dolomieu*) | Centrarchidae | 1998 | 3.83 | 9 | 17 | | 38 |
| Fish | Smallmouth Bass (*Micropterus dolomieu*) | Centrarchidae | 1999 | 4.35 | 11 | 17 | | 44 |
| Fish | Smallmouth Bass (*Micropterus dolomieu*) | Centrarchidae | 2000 | 3.67 | 14 | 17 | | 56 |
| Fish | Smallmouth Bass (*Micropterus dolomieu*) | Centrarchidae | 2001 | 12.75 | 5 | 17 | | 20 |
| Fish | Smallmouth Bass (*Micropterus dolomieu*) | Centrarchidae | 2002 | 8.66 | 8 | 17 | | 32 |
| Fish | Smallmouth Bass (*Micropterus dolomieu*) | Centrarchidae | 2003 | 9.58 | 6 | 17 | | 24 |
| Fish | Smallmouth Bass (*Micropterus dolomieu*) | Centrarchidae | 2004 | 5.58 | 12 | 17 | | 48 |
| Fish | Smallmouth Bass (*Micropterus dolomieu*) | Centrarchidae | 2005 | 8.92 | 7 | 17 | | 25 |
| Fish | Smallmouth Bass (*Micropterus dolomieu*) | Centrarchidae | 2006 | 7.70 | 6 | 17 | | 27 |
| Fish | Smallmouth Bass (*Micropterus dolomieu*) | Centrarchidae | 2007 | 10.33 | 5 | 17 | | 23 |
| Fish | Smallmouth Bass (*Micropterus dolomieu*) | Centrarchidae | 2008 | 8.83 | 6 | 17 | | 24 |
| Fish | Smallmouth Bass (*Micropterus dolomieu*) | Centrarchidae | 2009 | 4.42 | 10 | 17 | | 45 |
| Fish | Smallmouth Bass (*Micropterus dolomieu*) | Centrarchidae | 2010 | 8.00 | 6 | 17 | | 27 |
| Fish | Smallmouth Bass (*Micropterus dolomieu*) | Centrarchidae | 2011 | 6.17 | 11 | 17 | | 48 |
| Fish | Spotfin Shiner (*Cyprinella spiloptera*) | Cyprinidae | 2000 | 0.33 | 21 | 3 | | 84 |
| Fish | Spotfin Shiner (*Cyprinella spiloptera*) | Cyprinidae | 2004 | 0.08 | 25 | 3 | | 100 |
| Fish | Spotfin Shiner (*Cyprinella spiloptera*) | Cyprinidae | 2006 | 0.10 | 22 | 3 | | 100 |
| Fish | Walleye (*Sander vitreus*) | Percidae | 1995 | 1.00 | 13 | 17 | | 57 |
| Fish | Walleye (*Sander vitreus*) | Percidae | 1996 | 1.17 | 10 | 17 | | 42 |
| Fish | Walleye (*Sander vitreus*) | Percidae | 1997 | 2.00 | 10 | 17 | | 50 |
| Fish | Walleye (*Sander vitreus*) | Percidae | 1998 | 2.58 | 10 | 17 | | 42 |
| Fish | Walleye (*Sander vitreus*) | Percidae | 1999 | 2.21 | 14 | 17 | | 56 |
| Fish | Walleye (*Sander vitreus*) | Percidae | 2000 | 4.51 | 12 | 17 | | 48 |
| Fish | Walleye (*Sander vitreus*) | Percidae | 2001 | 5.33 | 10 | 17 | | 40 |
| Fish | Walleye (*Sander vitreus*) | Percidae | 2002 | 7.12 | 10 | 17 | | 40 |
| Fish | Walleye (*Sander vitreus*) | Percidae | 2003 | 4.67 | 9 | 17 | | 36 |
| Fish | Walleye (*Sander vitreus*) | Percidae | 2004 | 9.75 | 8 | 17 | | 32 |
| Fish | Walleye (*Sander vitreus*) | Percidae | 2005 | 7.02 | 9 | 17 | | 32 |
| Fish | Walleye (*Sander vitreus*) | Percidae | 2006 | 2.67 | 9 | 17 | | 41 |
| Fish | Walleye (*Sander vitreus*) | Percidae | 2007 | 4.17 | 9 | 17 | | 41 |
| Fish | Walleye (*Sander vitreus*) | Percidae | 2008 | 6.83 | 9 | 17 | | 36 |
| Fish | Walleye (*Sander vitreus*) | Percidae | 2009 | 4.83 | 9 | 17 | | 41 |
| Fish | Walleye (*Sander vitreus*) | Percidae | 2010 | 1.67 | 15 | 17 | | 68 |
| Fish | Walleye (*Sander vitreus*) | Percidae | 2011 | 3.17 | 14 | 17 | | 61 |
| Fish | White Bass (*Morone chrysops*) | Moronidae | 1995 | 20.48 | 4 | 17 | | 17 |
| Fish | White Bass (*Morone chrysops*) | Moronidae | 1996 | 19.90 | 4 | 17 | | 17 |
| Fish | White Bass (*Morone chrysops*) | Moronidae | 1997 | 12.25 | 3 | 17 | | 15 |
| Fish | White Bass (*Morone chrysops*) | Moronidae | 1998 | 29.43 | 1 | 17 | | 4 |
| Fish | White Bass (*Morone chrysops*) | Moronidae | 1999 | 40.51 | 3 | 17 | | 12 |
| Fish | White Bass (*Morone chrysops*) | Moronidae | 2000 | 27.94 | 4 | 17 | | 16 |
| Fish | White Bass (*Morone chrysops*) | Moronidae | 2001 | 25.77 | 4 | 17 | | 16 |
| Fish | White Bass (*Morone chrysops*) | Moronidae | 2002 | 27.91 | 3 | 17 | | 12 |
| Fish | White Bass (*Morone chrysops*) | Moronidae | 2003 | 15.13 | 5 | 17 | | 20 |
| Fish | White Bass (*Morone chrysops*) | Moronidae | 2004 | 10.42 | 7 | 17 | | 28 |
| Fish | White Bass (*Morone chrysops*) | Moronidae | 2005 | 49.82 | 2 | 17 | | 7 |
| Fish | White Bass (*Morone chrysops*) | Moronidae | 2006 | 11.47 | 4 | 17 | | 18 |
| Fish | White Bass (*Morone chrysops*) | Moronidae | 2007 | 17.47 | 4 | 17 | | 18 |
| Fish | White Bass (*Morone chrysops*) | Moronidae | 2008 | 28.36 | 4 | 17 | | 16 |
| Fish | White Bass (*Morone chrysops*) | Moronidae | 2009 | 24.79 | 3 | 17 | | 14 |
| Fish | White Bass (*Morone chrysops*) | Moronidae | 2010 | 30.59 | 3 | 17 | | 14 |
| Fish | White Bass (*Morone chrysops*) | Moronidae | 2011 | 15.00 | 4 | 17 | | 17 |
| Fish | White Crappie (*Pomoxis annularis*) | Centrarchidae | 1996 | 0.28 | 15 | 8 | | 63 |
| Fish | White Crappie (*Pomoxis annularis*) | Centrarchidae | 1997 | 0.33 | 16 | 8 | | 80 |
| Fish | White Crappie (*Pomoxis annularis*) | Centrarchidae | 1998 | 0.17 | 22 | 8 | | 92 |
| Fish | White Crappie (*Pomoxis annularis*) | Centrarchidae | 1999 | 0.08 | 25 | 8 | | 100 |
| Fish | White Crappie (*Pomoxis annularis*) | Centrarchidae | 2000 | 2.58 | 15 | 8 | | 60 |
| Fish | White Crappie (*Pomoxis annularis*) | Centrarchidae | 2002 | 0.20 | 24 | 8 | | 96 |
| Fish | White Crappie (*Pomoxis annularis*) | Centrarchidae | 2005 | 0.17 | 26 | 8 | | 93 |
| Fish | White Crappie (*Pomoxis annularis*) | Centrarchidae | 2010 | 0.17 | 22 | 8 | | 100 |
| Fish | White Sucker (*Catostomus commersonii*) | Catostomidae | 1995 | 1.25 | 11 | 17 | | 48 |
| Fish | White Sucker (*Catostomus commersonii*) | Catostomidae | 1996 | 0.25 | 16 | 17 | | 67 |
| Fish | White Sucker (*Catostomus commersonii*) | Catostomidae | 1997 | 1.33 | 13 | 17 | | 65 |
| Fish | White Sucker (*Catostomus commersonii*) | Catostomidae | 1998 | 0.83 | 15 | 17 | | 63 |
| Fish | White Sucker (*Catostomus commersonii*) | Catostomidae | 1999 | 2.50 | 13 | 17 | | 52 |
| Fish | White Sucker (*Catostomus commersonii*) | Catostomidae | 2000 | 2.00 | 18 | 17 | | 72 |
| Fish | White Sucker (*Catostomus commersonii*) | Catostomidae | 2001 | 1.33 | 16 | 17 | | 64 |
| Fish | White Sucker (*Catostomus commersonii*) | Catostomidae | 2002 | 3.83 | 13 | 17 | | 52 |
| Fish | White Sucker (*Catostomus commersonii*) | Catostomidae | 2003 | 2.00 | 15 | 17 | | 60 |
| Fish | White Sucker (*Catostomus commersonii*) | Catostomidae | 2004 | 1.50 | 16 | 17 | | 64 |
| Fish | White Sucker (*Catostomus commersonii*) | Catostomidae | 2005 | 1.75 | 16 | 17 | | 57 |
| Fish | White Sucker (*Catostomus commersonii*) | Catostomidae | 2006 | 1.00 | 17 | 17 | | 77 |
| Fish | White Sucker (*Catostomus commersonii*) | Catostomidae | 2007 | 1.50 | 13 | 17 | | 59 |
| Fish | White Sucker (*Catostomus commersonii*) | Catostomidae | 2008 | 1.33 | 16 | 17 | | 64 |
| Fish | White Sucker (*Catostomus commersonii*) | Catostomidae | 2009 | 1.67 | 16 | 17 | | 73 |
| Fish | White Sucker (*Catostomus commersonii*) | Catostomidae | 2010 | 2.83 | 12 | 17 | | 55 |
| Fish | White Sucker (*Catostomus commersonii*) | Catostomidae | 2011 | 1.83 | 16 | 17 | | 70 |
| Fish | Yellow Bass (*Morone mississippiensis*) | Moronidae | 1997 | 0.10 | 20 | 8 | | 100 |
| Fish | Yellow Bass (*Morone mississippiensis*) | Moronidae | 1999 | 33.27 | 4 | 8 | | 16 |
| Fish | Yellow Bass (*Morone mississippiensis*) | Moronidae | 2001 | 0.50 | 21 | 8 | | 84 |
| Fish | Yellow Bass (*Morone mississippiensis*) | Moronidae | 2002 | 0.33 | 23 | 8 | | 92 |
| Fish | Yellow Bass (*Morone mississippiensis*) | Moronidae | 2003 | 5.83 | 8 | 8 | | 32 |
| Fish | Yellow Bass (*Morone mississippiensis*) | Moronidae | 2004 | 0.33 | 23 | 8 | | 92 |
| Fish | Yellow Bass (*Morone mississippiensis*) | Moronidae | 2005 | 1.25 | 18 | 8 | | 64 |
| Fish | Yellow Bass (*Morone mississippiensis*) | Moronidae | 2011 | 4.67 | 12 | 8 | | 52 |
| Fish | Yellow bullhead (*Ameiurus natalis*) | Ictaluridae | 1995 | 0.17 | 22 | 13 | | 96 |
| Fish | Yellow bullhead (*Ameiurus natalis*) | Ictaluridae | 1996 | 0.06 | 21 | 13 | | 88 |
| Fish | Yellow bullhead (*Ameiurus natalis*) | Ictaluridae | 1997 | 0.33 | 18 | 13 | | 90 |
| Fish | Yellow bullhead (*Ameiurus natalis*) | Ictaluridae | 1998 | 1.00 | 14 | 13 | | 58 |
| Fish | Yellow bullhead (*Ameiurus natalis*) | Ictaluridae | 1999 | 5.50 | 9 | 13 | | 36 |
| Fish | Yellow bullhead (*Ameiurus natalis*) | Ictaluridae | 2000 | 7.50 | 7 | 13 | | 28 |
| Fish | Yellow bullhead (*Ameiurus natalis*) | Ictaluridae | 2001 | 8.33 | 8 | 13 | | 32 |
| Fish | Yellow bullhead (*Ameiurus natalis*) | Ictaluridae | 2003 | 1.17 | 19 | 13 | | 76 |
| Fish | Yellow bullhead (*Ameiurus natalis*) | Ictaluridae | 2005 | 3.00 | 14 | 13 | | 50 |
| Fish | Yellow bullhead (*Ameiurus natalis*) | Ictaluridae | 2008 | 0.67 | 21 | 13 | | 84 |
| Fish | Yellow bullhead (*Ameiurus natalis*) | Ictaluridae | 2009 | 2.67 | 12 | 13 | | 55 |
| Fish | Yellow bullhead (*Ameiurus natalis*) | Ictaluridae | 2010 | 2.00 | 13 | 13 | | 59 |
| Fish | Yellow bullhead (*Ameiurus natalis*) | Ictaluridae | 2011 | 0.17 | 21 | 13 | | 91 |
| Fish | Yellow Perch (*Perca flavescens*) | Percidae | 1995 | 50.12 | 2 | 17 | | 9 |
| Fish | Yellow Perch (*Perca flavescens*) | Percidae | 1996 | 140.99 | 1 | 17 | | 4 |
| Fish | Yellow Perch (*Perca flavescens*) | Percidae | 1997 | 81.02 | 1 | 17 | | 5 |
| Fish | Yellow Perch (*Perca flavescens*) | Percidae | 1998 | 23.59 | 3 | 17 | | 13 |
| Fish | Yellow Perch (*Perca flavescens*) | Percidae | 1999 | 376.11 | 1 | 17 | | 4 |
| Fish | Yellow Perch (*Perca flavescens*) | Percidae | 2000 | 238.50 | 1 | 17 | | 4 |
| Fish | Yellow Perch (*Perca flavescens*) | Percidae | 2001 | 66.83 | 2 | 17 | | 8 |
| Fish | Yellow Perch (*Perca flavescens*) | Percidae | 2002 | 16.75 | 4 | 17 | | 16 |
| Fish | Yellow Perch (*Perca flavescens*) | Percidae | 2003 | 5.83 | 7 | 17 | | 28 |
| Fish | Yellow Perch (*Perca flavescens*) | Percidae | 2004 | 62.40 | 1 | 17 | | 4 |
| Fish | Yellow Perch (*Perca flavescens*) | Percidae | 2005 | 28.98 | 4 | 17 | | 14 |
| Fish | Yellow Perch (*Perca flavescens*) | Percidae | 2006 | 86.76 | 2 | 17 | | 9 |
| Fish | Yellow Perch (*Perca flavescens*) | Percidae | 2007 | 47.57 | 2 | 17 | | 9 |
| Fish | Yellow Perch (*Perca flavescens*) | Percidae | 2008 | 34.83 | 3 | 17 | | 12 |
| Fish | Yellow Perch (*Perca flavescens*) | Percidae | 2009 | 10.97 | 6 | 17 | | 27 |
| Fish | Yellow Perch (*Perca flavescens*) | Percidae | 2010 | 8.73 | 5 | 17 | | 23 |
| Fish | Yellow Perch (*Perca flavescens*) | Percidae | 2011 | 72.07 | 2 | 17 | | 9 |
| Phytoplankton | *Achnanthes exigua* | Bacillariophyta | 1999 | 4.38 | 71 | 1 | | 99 |
| Phytoplankton | *Achnanthes minutissima* | Bacillariophyta | 1995 | 18.79 | 50 | 7 | | 70 |
| Phytoplankton | *Achnanthes minutissima* | Bacillariophyta | 1996 | 34.73 | 38 | 7 | | 58 |
| Phytoplankton | *Achnanthes minutissima* | Bacillariophyta | 1998 | 5.21 | 64 | 7 | | 89 |
| Phytoplankton | *Achnanthes minutissima* | Bacillariophyta | 1999 | 5.79 | 67 | 7 | | 93 |
| Phytoplankton | *Achnanthes minutissima* | Bacillariophyta | 2000 | 6.95 | 79 | 7 | | 99 |
| Phytoplankton | *Achnanthes minutissima* | Bacillariophyta | 2001 | 52.09 | 50 | 7 | | 68 |
| Phytoplankton | *Achnanthes minutissima* | Bacillariophyta | 2005 | 54.53 | 43 | 7 | | 65 |
| Phytoplankton | *Actinastrum hantzschii* | Chlorophyta | 1996 | 6.58 | 55 | 6 | | 83 |
| Phytoplankton | *Actinastrum hantzschii* | Chlorophyta | 2000 | 781.30 | 21 | 6 | | 26 |
| Phytoplankton | *Actinastrum hantzschii* | Chlorophyta | 2001 | 231.50 | 32 | 6 | | 43 |
| Phytoplankton | *Actinastrum hantzschii* | Chlorophyta | 2008 | 151.46 | 30 | 6 | | 45 |
| Phytoplankton | *Actinastrum hantzschii* | Chlorophyta | 2009 | 454.38 | 29 | 6 | | 36 |
| Phytoplankton | *Actinastrum hantzschii* | Chlorophyta | 2010 | 181.75 | 49 | 6 | | 57 |
| Phytoplankton | *Amphidinium sp. 1* | Pyrrhophyta | 1995 | 312.52 | 22 | 1 | | 31 |
| Phytoplankton | *Amphora pediculus* | Bacillariophyta | 1998 | 4.34 | 71 | 2 | | 99 |
| Phytoplankton | *Amphora pediculus* | Bacillariophyta | 1999 | 5.79 | 69 | 2 | | 96 |
| Phytoplankton | *Anabaena aphanizomenoides* | Cyanophyta | 1998 | 347.26 | 23 | 6 | | 32 |
| Phytoplankton | *Anabaena aphanizomenoides* | Cyanophyta | 2001 | 1266.00 | 22 | 6 | | 30 |
| Phytoplankton | *Anabaena aphanizomenoides* | Cyanophyta | 2004 | 105.33 | 25 | 6 | | 40 |
| Phytoplankton | *Anabaena aphanizomenoides* | Cyanophyta | 2005 | 1135.96 | 17 | 6 | | 26 |
| Phytoplankton | *Anabaena aphanizomenoides* | Cyanophyta | 2006 | 757.31 | 23 | 6 | | 35 |
| Phytoplankton | *Anabaena aphanizomenoides* | Cyanophyta | 2010 | 151.46 | 52 | 6 | | 60 |
| Phytoplankton | *Anabaena augstumalis* | Cyanophyta | 2001 | 350.72 | 30 | 8 | | 41 |
| Phytoplankton | *Anabaena augstumalis* | Cyanophyta | 2004 | 274.31 | 19 | 8 | | 30 |
| Phytoplankton | *Anabaena augstumalis* | Cyanophyta | 2005 | 6488.61 | 8 | 8 | | 12 |
| Phytoplankton | *Anabaena augstumalis* | Cyanophyta | 2006 | 227.19 | 31 | 8 | | 48 |
| Phytoplankton | *Anabaena augstumalis* | Cyanophyta | 2007 | 5298.13 | 9 | 8 | | 12 |
| Phytoplankton | *Anabaena augstumalis* | Cyanophyta | 2008 | 58962.66 | 5 | 8 | | 7 |
| Phytoplankton | *Anabaena augstumalis* | Cyanophyta | 2009 | 3029.24 | 15 | 8 | | 19 |
| Phytoplankton | *Anabaena augstumalis* | Cyanophyta | 2010 | 1514.62 | 18 | 8 | | 21 |
| Phytoplankton | *Anabaena circinalis* | Cyanophyta | 1995 | 109.60 | 32 | 15 | | 45 |
| Phytoplankton | *Anabaena circinalis* | Cyanophyta | 1997 | 1497.50 | 12 | 15 | | 20 |
| Phytoplankton | *Anabaena circinalis* | Cyanophyta | 1998 | 1601.67 | 12 | 15 | | 17 |
| Phytoplankton | *Anabaena circinalis* | Cyanophyta | 2000 | 347.24 | 26 | 15 | | 33 |
| Phytoplankton | *Anabaena circinalis* | Cyanophyta | 2001 | 3617.14 | 12 | 15 | | 16 |
| Phytoplankton | *Anabaena circinalis* | Cyanophyta | 2002 | 74.41 | 31 | 15 | | 63 |
| Phytoplankton | *Anabaena circinalis* | Cyanophyta | 2003 | 15.90 | 41 | 15 | | 80 |
| Phytoplankton | *Anabaena circinalis* | Cyanophyta | 2004 | 21.94 | 42 | 15 | | 67 |
| Phytoplankton | *Anabaena circinalis* | Cyanophyta | 2005 | 2351.44 | 14 | 15 | | 21 |
| Phytoplankton | *Anabaena circinalis* | Cyanophyta | 2006 | 1817.54 | 19 | 15 | | 29 |
| Phytoplankton | *Anabaena circinalis* | Cyanophyta | 2007 | 643.71 | 25 | 15 | | 34 |
| Phytoplankton | *Anabaena circinalis* | Cyanophyta | 2008 | 4389.36 | 14 | 15 | | 21 |
| Phytoplankton | *Anabaena circinalis* | Cyanophyta | 2009 | 7909.65 | 11 | 15 | | 14 |
| Phytoplankton | *Anabaena circinalis* | Cyanophyta | 2010 | 1018.23 | 28 | 15 | | 33 |
| Phytoplankton | *Anabaena circinalis* | Cyanophyta | 2011 | 81.81 | 42 | 15 | | 57 |
| Phytoplankton | *Anabaena flos-aquae* | Cyanophyta | 1995 | 9416.51 | 11 | 10 | | 15 |
| Phytoplankton | *Anabaena flos-aquae* | Cyanophyta | 1996 | 5080.87 | 10 | 10 | | 15 |
| Phytoplankton | *Anabaena flos-aquae* | Cyanophyta | 1997 | 989.62 | 13 | 10 | | 22 |
| Phytoplankton | *Anabaena flos-aquae* | Cyanophyta | 1998 | 208.35 | 26 | 10 | | 36 |
| Phytoplankton | *Anabaena flos-aquae* | Cyanophyta | 1999 | 2994.99 | 13 | 10 | | 18 |
| Phytoplankton | *Anabaena flos-aquae* | Cyanophyta | 2000 | 3858.77 | 13 | 10 | | 16 |
| Phytoplankton | *Anabaena flos-aquae* | Cyanophyta | 2001 | 1639.77 | 21 | 10 | | 28 |
| Phytoplankton | *Anabaena flos-aquae* | Cyanophyta | 2002 | 10309.31 | 7 | 10 | | 14 |
| Phytoplankton | *Anabaena flos-aquae* | Cyanophyta | 2003 | 636.14 | 15 | 10 | | 29 |
| Phytoplankton | *Anabaena flos-aquae* | Cyanophyta | 2004 | 27.43 | 40 | 10 | | 63 |
| Phytoplankton | *Anabaena lemmermannii* | Cyanophyta | 2007 | 5053.77 | 10 | 3 | | 14 |
| Phytoplankton | *Anabaena lemmermannii* | Cyanophyta | 2009 | 3281.67 | 14 | 3 | | 17 |
| Phytoplankton | *Anabaena lemmermannii* | Cyanophyta | 2010 | 2726.31 | 15 | 3 | | 17 |
| Phytoplankton | *Anabaena macrospora* | Cyanophyta | 2001 | 795.77 | 25 | 6 | | 34 |
| Phytoplankton | *Anabaena macrospora* | Cyanophyta | 2004 | 3.66 | 60 | 6 | | 95 |
| Phytoplankton | *Anabaena macrospora* | Cyanophyta | 2005 | 1235.93 | 16 | 6 | | 24 |
| Phytoplankton | *Anabaena macrospora* | Cyanophyta | 2006 | 212.05 | 33 | 6 | | 51 |
| Phytoplankton | *Anabaena macrospora* | Cyanophyta | 2007 | 21.94 | 60 | 6 | | 82 |
| Phytoplankton | *Anabaena macrospora* | Cyanophyta | 2010 | 1022.37 | 27 | 6 | | 31 |
| Phytoplankton | *Anabaena mendotae* | Cyanophyta | 2004 | 1991.26 | 12 | 6 | | 19 |
| Phytoplankton | *Anabaena mendotae* | Cyanophyta | 2005 | 48.45 | 44 | 6 | | 67 |
| Phytoplankton | *Anabaena mendotae* | Cyanophyta | 2006 | 11132.43 | 6 | 6 | | 9 |
| Phytoplankton | *Anabaena mendotae* | Cyanophyta | 2007 | 4259.86 | 11 | 6 | | 15 |
| Phytoplankton | *Anabaena mendotae* | Cyanophyta | 2009 | 2048.17 | 18 | 6 | | 22 |
| Phytoplankton | *Anabaena mendotae* | Cyanophyta | 2011 | 1439.77 | 15 | 6 | | 20 |
| Phytoplankton | *Anabaena oscillarioides* | Cyanophyta | 2010 | 1009.75 | 29 | 1 | | 34 |
| Phytoplankton | *Anabaena spiroides* | Cyanophyta | 1997 | 328.80 | 17 | 2 | | 28 |
| Phytoplankton | *Anabaena spiroides* | Cyanophyta | 2008 | 1635.79 | 17 | 2 | | 25 |
| Phytoplankton | *Anabaenopsis circularis* | Cyanophyta | 2004 | 17.56 | 43 | 1 | | 68 |
| Phytoplankton | *Ankistrodesmus braunii* | Chlorophyta | 2008 | 3.03 | 67 | 2 | | 100 |
| Phytoplankton | *Ankistrodesmus braunii* | Chlorophyta | 2010 | 100.97 | 61 | 2 | | 71 |
| Phytoplankton | *Ankistrodesmus convolutus* | Chlorophyta | 1995 | 69.45 | 41 | 8 | | 58 |
| Phytoplankton | *Ankistrodesmus convolutus* | Chlorophyta | 1999 | 43.41 | 47 | 8 | | 65 |
| Phytoplankton | *Ankistrodesmus convolutus* | Chlorophyta | 2000 | 17.36 | 69 | 8 | | 86 |
| Phytoplankton | *Ankistrodesmus convolutus* | Chlorophyta | 2001 | 72.34 | 43 | 8 | | 58 |
| Phytoplankton | *Ankistrodesmus convolutus* | Chlorophyta | 2002 | 6.06 | 43 | 8 | | 88 |
| Phytoplankton | *Ankistrodesmus convolutus* | Chlorophyta | 2005 | 37.87 | 47 | 8 | | 71 |
| Phytoplankton | *Ankistrodesmus convolutus* | Chlorophyta | 2010 | 50.49 | 68 | 8 | | 79 |
| Phytoplankton | *Ankistrodesmus convolutus* | Chlorophyta | 2011 | 25.41 | 56 | 8 | | 76 |
| Phytoplankton | *Ankistrodesmus falcatus* | Chlorophyta | 2002 | 7.23 | 41 | 10 | | 84 |
| Phytoplankton | *Ankistrodesmus falcatus* | Chlorophyta | 2003 | 9.09 | 43 | 10 | | 84 |
| Phytoplankton | *Ankistrodesmus falcatus* | Chlorophyta | 2004 | 16.23 | 45 | 10 | | 71 |
| Phytoplankton | *Ankistrodesmus falcatus* | Chlorophyta | 2005 | 6.49 | 60 | 10 | | 91 |
| Phytoplankton | *Ankistrodesmus falcatus* | Chlorophyta | 2006 | 6.06 | 60 | 10 | | 92 |
| Phytoplankton | *Ankistrodesmus falcatus* | Chlorophyta | 2007 | 30.29 | 53 | 10 | | 73 |
| Phytoplankton | *Ankistrodesmus falcatus* | Chlorophyta | 2008 | 9.09 | 54 | 10 | | 81 |
| Phytoplankton | *Ankistrodesmus falcatus* | Chlorophyta | 2009 | 50.49 | 54 | 10 | | 67 |
| Phytoplankton | *Ankistrodesmus falcatus* | Chlorophyta | 2010 | 151.46 | 51 | 10 | | 59 |
| Phytoplankton | *Ankistrodesmus falcatus* | Chlorophyta | 2011 | 105.87 | 36 | 10 | | 49 |
| Phytoplankton | *Anomoeoneis vitrea* | Bacillariophyta | 2003 | 2.19 | 49 | 2 | | 96 |
| Phytoplankton | *Anomoeoneis vitrea* | Bacillariophyta | 2010 | 50.49 | 70 | 2 | | 81 |
| Phytoplankton | *Aphanizomenon flos-aquae* | Cyanophyta | 1995 | 92260.90 | 4 | 17 | | 6 |
| Phytoplankton | *Aphanizomenon flos-aquae* | Cyanophyta | 1996 | 113885.03 | 2 | 17 | | 3 |
| Phytoplankton | *Aphanizomenon flos-aquae* | Cyanophyta | 1997 | 28677.56 | 4 | 17 | | 7 |
| Phytoplankton | *Aphanizomenon flos-aquae* | Cyanophyta | 1998 | 58566.82 | 5 | 17 | | 7 |
| Phytoplankton | *Aphanizomenon flos-aquae* | Cyanophyta | 1999 | 89231.00 | 3 | 17 | | 4 |
| Phytoplankton | *Aphanizomenon flos-aquae* | Cyanophyta | 2000 | 138847.25 | 1 | 17 | | 1 |
| Phytoplankton | *Aphanizomenon flos-aquae* | Cyanophyta | 2001 | 105297.27 | 3 | 17 | | 4 |
| Phytoplankton | *Aphanizomenon flos-aquae* | Cyanophyta | 2002 | 6904.08 | 8 | 17 | | 16 |
| Phytoplankton | *Aphanizomenon flos-aquae* | Cyanophyta | 2003 | 20536.65 | 4 | 17 | | 8 |
| Phytoplankton | *Aphanizomenon flos-aquae* | Cyanophyta | 2004 | 53215.91 | 2 | 17 | | 3 |
| Phytoplankton | *Aphanizomenon flos-aquae* | Cyanophyta | 2005 | 81165.15 | 2 | 17 | | 3 |
| Phytoplankton | *Aphanizomenon flos-aquae* | Cyanophyta | 2006 | 35945.38 | 3 | 17 | | 5 |
| Phytoplankton | *Aphanizomenon flos-aquae* | Cyanophyta | 2007 | 54372.15 | 5 | 17 | | 7 |
| Phytoplankton | *Aphanizomenon flos-aquae* | Cyanophyta | 2008 | 212061.07 | 1 | 17 | | 1 |
| Phytoplankton | *Aphanizomenon flos-aquae* | Cyanophyta | 2009 | 123989.63 | 1 | 17 | | 1 |
| Phytoplankton | *Aphanizomenon flos-aquae* | Cyanophyta | 2010 | 43034.78 | 5 | 17 | | 6 |
| Phytoplankton | *Aphanizomenon flos-aquae* | Cyanophyta | 2011 | 56951.76 | 5 | 17 | | 7 |
| Phytoplankton | *Aphanizomenon gracile* | Cyanophyta | 2011 | 95.87 | 39 | 1 | | 53 |
| Phytoplankton | *Aphanizomenon issatschenkoi* | Cyanophyta | 1998 | 1606.01 | 11 | 12 | | 15 |
| Phytoplankton | *Aphanizomenon issatschenkoi* | Cyanophyta | 1999 | 2630.38 | 14 | 12 | | 19 |
| Phytoplankton | *Aphanizomenon issatschenkoi* | Cyanophyta | 2000 | 6191.82 | 10 | 12 | | 13 |
| Phytoplankton | *Aphanizomenon issatschenkoi* | Cyanophyta | 2001 | 2130.10 | 16 | 12 | | 22 |
| Phytoplankton | *Aphanizomenon issatschenkoi* | Cyanophyta | 2002 | 868.11 | 14 | 12 | | 29 |
| Phytoplankton | *Aphanizomenon issatschenkoi* | Cyanophyta | 2004 | 60.47 | 31 | 12 | | 49 |
| Phytoplankton | *Aphanizomenon issatschenkoi* | Cyanophyta | 2005 | 403.90 | 25 | 12 | | 38 |
| Phytoplankton | *Aphanizomenon issatschenkoi* | Cyanophyta | 2006 | 605.85 | 25 | 12 | | 38 |
| Phytoplankton | *Aphanizomenon issatschenkoi* | Cyanophyta | 2007 | 151.46 | 38 | 12 | | 52 |
| Phytoplankton | *Aphanizomenon issatschenkoi* | Cyanophyta | 2009 | 5679.81 | 13 | 12 | | 16 |
| Phytoplankton | *Aphanizomenon issatschenkoi* | Cyanophyta | 2010 | 3054.47 | 14 | 12 | | 16 |
| Phytoplankton | *Aphanizomenon issatschenkoi* | Cyanophyta | 2011 | 409.02 | 23 | 12 | | 31 |
| Phytoplankton | *Aphanocapsa delicatissima* | Cyanophyta | 1995 | 686244.13 | 1 | 16 | | 1 |
| Phytoplankton | *Aphanocapsa delicatissima* | Cyanophyta | 1997 | 58024.87 | 3 | 16 | | 5 |
| Phytoplankton | *Aphanocapsa delicatissima* | Cyanophyta | 1998 | 645111.06 | 1 | 16 | | 1 |
| Phytoplankton | *Aphanocapsa delicatissima* | Cyanophyta | 1999 | 135946.65 | 2 | 16 | | 3 |
| Phytoplankton | *Aphanocapsa delicatissima* | Cyanophyta | 2000 | 40478.08 | 5 | 16 | | 6 |
| Phytoplankton | *Aphanocapsa delicatissima* | Cyanophyta | 2001 | 213894.62 | 1 | 16 | | 1 |
| Phytoplankton | *Aphanocapsa delicatissima* | Cyanophyta | 2002 | 201836.51 | 2 | 16 | | 4 |
| Phytoplankton | *Aphanocapsa delicatissima* | Cyanophyta | 2003 | 31579.74 | 2 | 16 | | 4 |
| Phytoplankton | *Aphanocapsa delicatissima* | Cyanophyta | 2004 | 95966.06 | 1 | 16 | | 2 |
| Phytoplankton | *Aphanocapsa delicatissima* | Cyanophyta | 2005 | 642083.76 | 1 | 16 | | 2 |
| Phytoplankton | *Aphanocapsa delicatissima* | Cyanophyta | 2006 | 72095.74 | 2 | 16 | | 3 |
| Phytoplankton | *Aphanocapsa delicatissima* | Cyanophyta | 2007 | 326707.69 | 1 | 16 | | 1 |
| Phytoplankton | *Aphanocapsa delicatissima* | Cyanophyta | 2008 | 99373.95 | 2 | 16 | | 3 |
| Phytoplankton | *Aphanocapsa delicatissima* | Cyanophyta | 2009 | 93148.88 | 2 | 16 | | 2 |
| Phytoplankton | *Aphanocapsa delicatissima* | Cyanophyta | 2010 | 124410.55 | 4 | 16 | | 5 |
| Phytoplankton | *Aphanocapsa delicatissima* | Cyanophyta | 2011 | 124392.20 | 2 | 16 | | 3 |
| Phytoplankton | *Aphanocapsa elachista* | Cyanophyta | 1995 | 77240.98 | 5 | 12 | | 7 |
| Phytoplankton | *Aphanocapsa elachista* | Cyanophyta | 1996 | 76597.31 | 3 | 12 | | 5 |
| Phytoplankton | *Aphanocapsa elachista* | Cyanophyta | 1998 | 166.68 | 27 | 12 | | 38 |
| Phytoplankton | *Aphanocapsa elachista* | Cyanophyta | 1999 | 694.50 | 19 | 12 | | 26 |
| Phytoplankton | *Aphanocapsa elachista* | Cyanophyta | 2000 | 5903.25 | 11 | 12 | | 14 |
| Phytoplankton | *Aphanocapsa elachista* | Cyanophyta | 2001 | 8681.14 | 10 | 12 | | 14 |
| Phytoplankton | *Aphanocapsa elachista* | Cyanophyta | 2002 | 496.07 | 16 | 12 | | 33 |
| Phytoplankton | *Aphanocapsa elachista* | Cyanophyta | 2004 | 454.38 | 15 | 12 | | 24 |
| Phytoplankton | *Aphanocapsa elachista* | Cyanophyta | 2008 | 946.63 | 22 | 12 | | 33 |
| Phytoplankton | *Aphanocapsa elachista* | Cyanophyta | 2009 | 1817.54 | 21 | 12 | | 26 |
| Phytoplankton | *Aphanocapsa elachista* | Cyanophyta | 2010 | 1476.75 | 20 | 12 | | 23 |
| Phytoplankton | *Aphanocapsa elachista* | Cyanophyta | 2011 | 952.79 | 20 | 12 | | 27 |
| Phytoplankton | *Aphanocapsa holsatica* | Cyanophyta | 2009 | 58767.10 | 5 | 2 | | 6 |
| Phytoplankton | *Aphanocapsa holsatica* | Cyanophyta | 2010 | 17670.52 | 10 | 2 | | 12 |
| Phytoplankton | *Aphanocapsa incerta* | Cyanophyta | 2005 | 511.18 | 22 | 3 | | 33 |
| Phytoplankton | *Aphanocapsa incerta* | Cyanophyta | 2007 | 851.97 | 23 | 3 | | 32 |
| Phytoplankton | *Aphanocapsa incerta* | Cyanophyta | 2009 | 145.40 | 41 | 3 | | 51 |
| Phytoplankton | *Aphanocapsa koordersi* | Cyanophyta | 1996 | 21.92 | 43 | 1 | | 65 |
| Phytoplankton | *Aphanothece clathrata* | Cyanophyta | 1995 | 7813.05 | 13 | 4 | | 18 |
| Phytoplankton | *Aphanothece clathrata* | Cyanophyta | 2006 | 4922.50 | 12 | 4 | | 18 |
| Phytoplankton | *Aphanothece clathrata* | Cyanophyta | 2010 | 1371.54 | 22 | 4 | | 26 |
| Phytoplankton | *Aphanothece clathrata* | Cyanophyta | 2011 | 83457.27 | 3 | 4 | | 4 |
| Phytoplankton | *Aphanothece nidulans* | Cyanophyta | 1995 | 65108.53 | 6 | 17 | | 8 |
| Phytoplankton | *Aphanothece nidulans* | Cyanophyta | 1996 | 292227.66 | 1 | 17 | | 2 |
| Phytoplankton | *Aphanothece nidulans* | Cyanophyta | 1997 | 273022.29 | 1 | 17 | | 2 |
| Phytoplankton | *Aphanothece nidulans* | Cyanophyta | 1998 | 290994.69 | 2 | 17 | | 3 |
| Phytoplankton | *Aphanothece nidulans* | Cyanophyta | 1999 | 1244658.54 | 1 | 17 | | 1 |
| Phytoplankton | *Aphanothece nidulans* | Cyanophyta | 2000 | 105475.88 | 3 | 17 | | 4 |
| Phytoplankton | *Aphanothece nidulans* | Cyanophyta | 2001 | 390.60 | 29 | 17 | | 39 |
| Phytoplankton | *Aphanothece nidulans* | Cyanophyta | 2002 | 1157023.36 | 1 | 17 | | 2 |
| Phytoplankton | *Aphanothece nidulans* | Cyanophyta | 2003 | 213511.42 | 1 | 17 | | 2 |
| Phytoplankton | *Aphanothece nidulans* | Cyanophyta | 2004 | 5301.15 | 9 | 17 | | 14 |
| Phytoplankton | *Aphanothece nidulans* | Cyanophyta | 2005 | 36047.85 | 3 | 17 | | 5 |
| Phytoplankton | *Aphanothece nidulans* | Cyanophyta | 2006 | 312368.71 | 1 | 17 | | 2 |
| Phytoplankton | *Aphanothece nidulans* | Cyanophyta | 2007 | 162556.15 | 2 | 17 | | 3 |
| Phytoplankton | *Aphanothece nidulans* | Cyanophyta | 2008 | 5289.04 | 13 | 17 | | 19 |
| Phytoplankton | *Aphanothece nidulans* | Cyanophyta | 2009 | 30322.61 | 7 | 17 | | 9 |
| Phytoplankton | *Aphanothece nidulans* | Cyanophyta | 2010 | 149197.23 | 3 | 17 | | 3 |
| Phytoplankton | *Aphanothece nidulans* | Cyanophyta | 2011 | 208237.39 | 1 | 17 | | 1 |
| Phytoplankton | *Aphanothece saxicola* | Cyanophyta | 1995 | 35.07 | 46 | 2 | | 65 |
| Phytoplankton | *Aphanothece saxicola* | Cyanophyta | 2006 | 272.63 | 29 | 2 | | 45 |
| Phytoplankton | *Asterionella formosa* | Bacillariophyta | 1995 | 17.36 | 52 | 6 | | 73 |
| Phytoplankton | *Asterionella formosa* | Bacillariophyta | 2000 | 69.45 | 41 | 6 | | 51 |
| Phytoplankton | *Asterionella formosa* | Bacillariophyta | 2002 | 17.54 | 38 | 6 | | 78 |
| Phytoplankton | *Asterionella formosa* | Bacillariophyta | 2005 | 103.86 | 37 | 6 | | 56 |
| Phytoplankton | *Asterionella formosa* | Bacillariophyta | 2008 | 7.31 | 55 | 6 | | 82 |
| Phytoplankton | *Asterionella formosa* | Bacillariophyta | 2009 | 12.98 | 71 | 6 | | 88 |
| Phytoplankton | *Aulacoseira ambigua* | Bacillariophyta | 1999 | 260.43 | 23 | 11 | | 32 |
| Phytoplankton | *Aulacoseira ambigua* | Bacillariophyta | 2000 | 43.41 | 48 | 11 | | 60 |
| Phytoplankton | *Aulacoseira ambigua* | Bacillariophyta | 2001 | 73.98 | 41 | 11 | | 55 |
| Phytoplankton | *Aulacoseira ambigua* | Bacillariophyta | 2003 | 27.26 | 30 | 11 | | 59 |
| Phytoplankton | *Aulacoseira ambigua* | Bacillariophyta | 2004 | 1.77 | 62 | 11 | | 98 |
| Phytoplankton | *Aulacoseira ambigua* | Bacillariophyta | 2005 | 103.86 | 36 | 11 | | 55 |
| Phytoplankton | *Aulacoseira ambigua* | Bacillariophyta | 2006 | 8.78 | 55 | 11 | | 85 |
| Phytoplankton | *Aulacoseira ambigua* | Bacillariophyta | 2007 | 17.56 | 62 | 11 | | 85 |
| Phytoplankton | *Aulacoseira ambigua* | Bacillariophyta | 2008 | 72.70 | 35 | 11 | | 52 |
| Phytoplankton | *Aulacoseira ambigua* | Bacillariophyta | 2009 | 302.92 | 33 | 11 | | 41 |
| Phytoplankton | *Aulacoseira ambigua* | Bacillariophyta | 2010 | 8.78 | 83 | 11 | | 97 |
| Phytoplankton | *Aulacoseira granulata* | Bacillariophyta | 1995 | 13.15 | 56 | 14 | | 79 |
| Phytoplankton | *Aulacoseira granulata* | Bacillariophyta | 1996 | 47.86 | 32 | 14 | | 48 |
| Phytoplankton | *Aulacoseira granulata* | Bacillariophyta | 1997 | 500.03 | 15 | 14 | | 25 |
| Phytoplankton | *Aulacoseira granulata* | Bacillariophyta | 1998 | 260.43 | 24 | 14 | | 33 |
| Phytoplankton | *Aulacoseira granulata* | Bacillariophyta | 2000 | 455.76 | 24 | 14 | | 30 |
| Phytoplankton | *Aulacoseira granulata* | Bacillariophyta | 2001 | 1808.59 | 19 | 14 | | 26 |
| Phytoplankton | *Aulacoseira granulata* | Bacillariophyta | 2002 | 3.47 | 47 | 14 | | 96 |
| Phytoplankton | *Aulacoseira granulata* | Bacillariophyta | 2003 | 9.09 | 44 | 14 | | 86 |
| Phytoplankton | *Aulacoseira granulata* | Bacillariophyta | 2004 | 4.39 | 58 | 14 | | 92 |
| Phytoplankton | *Aulacoseira granulata* | Bacillariophyta | 2006 | 146.30 | 35 | 14 | | 54 |
| Phytoplankton | *Aulacoseira granulata* | Bacillariophyta | 2008 | 17.46 | 48 | 14 | | 72 |
| Phytoplankton | *Aulacoseira granulata* | Bacillariophyta | 2009 | 151.46 | 38 | 14 | | 47 |
| Phytoplankton | *Aulacoseira granulata* | Bacillariophyta | 2010 | 890.65 | 32 | 14 | | 37 |
| Phytoplankton | *Aulacoseira granulata* | Bacillariophyta | 2011 | 70.58 | 43 | 14 | | 58 |
| Phytoplankton | *Botryococcus braunii* | Chlorophyta | 1996 | 32.90 | 41 | 5 | | 62 |
| Phytoplankton | *Botryococcus braunii* | Chlorophyta | 2001 | 173.62 | 36 | 5 | | 49 |
| Phytoplankton | *Botryococcus braunii* | Chlorophyta | 2003 | 105.33 | 21 | 5 | | 41 |
| Phytoplankton | *Botryococcus braunii* | Chlorophyta | 2005 | 290.81 | 28 | 5 | | 42 |
| Phytoplankton | *Botryococcus braunii* | Chlorophyta | 2007 | 438.89 | 27 | 5 | | 37 |
| Phytoplankton | *Caloneis sp. 1* | Bacillariophyta | 1999 | 5.79 | 65 | 1 | | 90 |
| Phytoplankton | *Carteria platyrhyncha* | Chlorophyta | 1995 | 17.36 | 54 | 2 | | 76 |
| Phytoplankton | *Carteria platyrhyncha* | Chlorophyta | 2009 | 18.18 | 65 | 2 | | 80 |
| Phytoplankton | *Characium limneticum* | Chlorophyta | 2001 | 2.60 | 72 | 3 | | 97 |
| Phytoplankton | *Characium limneticum* | Chlorophyta | 2009 | 18.18 | 66 | 3 | | 81 |
| Phytoplankton | *Characium limneticum* | Chlorophyta | 2011 | 84.69 | 40 | 3 | | 54 |
| Phytoplankton | *Chlamydomonas augustae* | Chlorophyta | 1998 | 17.36 | 52 | 1 | | 72 |
| Phytoplankton | *Chlamydomonas globosa* | Chlorophyta | 1995 | 364.61 | 21 | 14 | | 30 |
| Phytoplankton | *Chlamydomonas globosa* | Chlorophyta | 1996 | 833.39 | 14 | 14 | | 21 |
| Phytoplankton | *Chlamydomonas globosa* | Chlorophyta | 1997 | 289.37 | 20 | 14 | | 33 |
| Phytoplankton | *Chlamydomonas globosa* | Chlorophyta | 1998 | 130.22 | 29 | 14 | | 40 |
| Phytoplankton | *Chlamydomonas globosa* | Chlorophyta | 1999 | 36.17 | 48 | 14 | | 67 |
| Phytoplankton | *Chlamydomonas globosa* | Chlorophyta | 2000 | 17.36 | 71 | 14 | | 89 |
| Phytoplankton | *Chlamydomonas globosa* | Chlorophyta | 2001 | 156.26 | 39 | 14 | | 53 |
| Phytoplankton | *Chlamydomonas globosa* | Chlorophyta | 2002 | 86.81 | 27 | 14 | | 55 |
| Phytoplankton | *Chlamydomonas globosa* | Chlorophyta | 2003 | 90.88 | 22 | 14 | | 43 |
| Phytoplankton | *Chlamydomonas globosa* | Chlorophyta | 2004 | 178.51 | 23 | 14 | | 37 |
| Phytoplankton | *Chlamydomonas globosa* | Chlorophyta | 2005 | 37.87 | 48 | 14 | | 73 |
| Phytoplankton | *Chlamydomonas globosa* | Chlorophyta | 2006 | 12.12 | 53 | 14 | | 82 |
| Phytoplankton | *Chlamydomonas globosa* | Chlorophyta | 2007 | 45.44 | 52 | 14 | | 71 |
| Phytoplankton | *Chlamydomonas globosa* | Chlorophyta | 2008 | 75.73 | 34 | 14 | | 51 |
| Phytoplankton | *Chlamydomonas incerta* | Chlorophyta | 1995 | 156.26 | 30 | 6 | | 42 |
| Phytoplankton | *Chlamydomonas incerta* | Chlorophyta | 1996 | 34.73 | 37 | 6 | | 56 |
| Phytoplankton | *Chlamydomonas incerta* | Chlorophyta | 1998 | 8.68 | 62 | 6 | | 86 |
| Phytoplankton | *Chlamydomonas incerta* | Chlorophyta | 1999 | 43.41 | 46 | 6 | | 64 |
| Phytoplankton | *Chlamydomonas incerta* | Chlorophyta | 2000 | 34.73 | 54 | 6 | | 68 |
| Phytoplankton | *Chlamydomonas incerta* | Chlorophyta | 2001 | 28.94 | 56 | 6 | | 76 |
| Phytoplankton | *Chlamydomonas inepta* | Chlorophyta | 1995 | 104.17 | 35 | 3 | | 49 |
| Phytoplankton | *Chlamydomonas inepta* | Chlorophyta | 1997 | 57.87 | 34 | 3 | | 57 |
| Phytoplankton | *Chlamydomonas inepta* | Chlorophyta | 1998 | 52.09 | 39 | 3 | | 54 |
| Phytoplankton | *Chlamydomonas platystigma* | Chlorophyta | 1996 | 17.36 | 45 | 10 | | 68 |
| Phytoplankton | *Chlamydomonas platystigma* | Chlorophyta | 1998 | 17.36 | 53 | 10 | | 74 |
| Phytoplankton | *Chlamydomonas platystigma* | Chlorophyta | 1999 | 173.62 | 32 | 10 | | 44 |
| Phytoplankton | *Chlamydomonas platystigma* | Chlorophyta | 2000 | 69.45 | 43 | 10 | | 54 |
| Phytoplankton | *Chlamydomonas platystigma* | Chlorophyta | 2002 | 86.81 | 29 | 10 | | 59 |
| Phytoplankton | *Chlamydomonas platystigma* | Chlorophyta | 2003 | 22.72 | 31 | 10 | | 61 |
| Phytoplankton | *Chlamydomonas platystigma* | Chlorophyta | 2004 | 90.88 | 29 | 10 | | 46 |
| Phytoplankton | *Chlamydomonas platystigma* | Chlorophyta | 2005 | 15.15 | 56 | 10 | | 85 |
| Phytoplankton | *Chlamydomonas platystigma* | Chlorophyta | 2006 | 27.26 | 49 | 10 | | 75 |
| Phytoplankton | *Chlamydomonas platystigma* | Chlorophyta | 2007 | 90.88 | 42 | 10 | | 58 |
| Phytoplankton | *Chlamydomonas pumilio* | Chlorophyta | 2003 | 54.53 | 25 | 1 | | 49 |
| Phytoplankton | *Chlorella vulgaris* | Chlorophyta | 1997 | 546.91 | 14 | 1 | | 23 |
| Phytoplankton | *Chlorocloster sp. 1* | Xanthophyta | 1995 | 4.38 | 67 | 1 | | 94 |
| Phytoplankton | *Chlorogonium sp. 1* | Chlorophyta | 2006 | 4.54 | 63 | 1 | | 97 |
| Phytoplankton | *Chloromonas pumilio* | Chlorophyta | 2002 | 91.15 | 25 | 1 | | 51 |
| Phytoplankton | *Chromulina sp. 1* | Chrysophyta | 2008 | 1417.68 | 19 | 4 | | 28 |
| Phytoplankton | *Chromulina sp. 1* | Chrysophyta | 2009 | 817.89 | 25 | 4 | | 31 |
| Phytoplankton | *Chromulina sp. 1* | Chrysophyta | 2010 | 1363.15 | 23 | 4 | | 27 |
| Phytoplankton | *Chromulina sp. 1* | Chrysophyta | 2011 | 1552.69 | 14 | 4 | | 19 |
| Phytoplankton | *Chroococcus limneticus* | Cyanophyta | 1995 | 13.48 | 55 | 6 | | 77 |
| Phytoplankton | *Chroococcus limneticus* | Cyanophyta | 1996 | 46.76 | 33 | 6 | | 50 |
| Phytoplankton | *Chroococcus limneticus* | Cyanophyta | 1997 | 35.07 | 38 | 6 | | 63 |
| Phytoplankton | *Chroococcus limneticus* | Cyanophyta | 1998 | 750.06 | 17 | 6 | | 24 |
| Phytoplankton | *Chroococcus limneticus* | Cyanophyta | 1999 | 210.43 | 28 | 6 | | 39 |
| Phytoplankton | *Chroococcus limneticus* | Cyanophyta | 2000 | 70.14 | 40 | 6 | | 50 |
| Phytoplankton | *Chroococcus minimus* | Cyanophyta | 1997 | 26.04 | 41 | 6 | | 68 |
| Phytoplankton | *Chroococcus minimus* | Cyanophyta | 2003 | 51.93 | 27 | 6 | | 53 |
| Phytoplankton | *Chroococcus minimus* | Cyanophyta | 2005 | 484.68 | 23 | 6 | | 35 |
| Phytoplankton | *Chroococcus minimus* | Cyanophyta | 2009 | 109.05 | 43 | 6 | | 53 |
| Phytoplankton | *Chroococcus minimus* | Cyanophyta | 2010 | 37.87 | 76 | 6 | | 88 |
| Phytoplankton | *Chroococcus minimus* | Cyanophyta | 2011 | 33.88 | 49 | 6 | | 66 |
| Phytoplankton | *Chroococcus minutus* | Cyanophyta | 1995 | 116.92 | 31 | 8 | | 44 |
| Phytoplankton | *Chroococcus minutus* | Cyanophyta | 2001 | 34.72 | 54 | 8 | | 73 |
| Phytoplankton | *Chroococcus minutus* | Cyanophyta | 2004 | 327.16 | 16 | 8 | | 25 |
| Phytoplankton | *Chroococcus minutus* | Cyanophyta | 2005 | 121.17 | 34 | 8 | | 52 |
| Phytoplankton | *Chroococcus minutus* | Cyanophyta | 2006 | 90.88 | 39 | 8 | | 60 |
| Phytoplankton | *Chroococcus minutus* | Cyanophyta | 2007 | 378.65 | 29 | 8 | | 40 |
| Phytoplankton | *Chroococcus minutus* | Cyanophyta | 2008 | 1817.54 | 16 | 8 | | 24 |
| Phytoplankton | *Chroococcus minutus* | Cyanophyta | 2011 | 327.22 | 25 | 8 | | 34 |
| Phytoplankton | *Chrysocapsa sp. 1* | Chrysophyta | 1996 | 40.92 | 34 | 1 | | 52 |
| Phytoplankton | *Chrysococcus minutus* | Chrysophyta | 1996 | 208.35 | 22 | 7 | | 33 |
| Phytoplankton | *Chrysococcus minutus* | Chrysophyta | 1998 | 5.21 | 68 | 7 | | 94 |
| Phytoplankton | *Chrysococcus minutus* | Chrysophyta | 2000 | 7.23 | 78 | 7 | | 98 |
| Phytoplankton | *Chrysococcus minutus* | Chrysophyta | 2001 | 52.09 | 49 | 7 | | 66 |
| Phytoplankton | *Chrysococcus minutus* | Chrysophyta | 2005 | 30.29 | 51 | 7 | | 77 |
| Phytoplankton | *Chrysococcus minutus* | Chrysophyta | 2006 | 218.10 | 32 | 7 | | 49 |
| Phytoplankton | *Chrysococcus minutus* | Chrysophyta | 2009 | 56.80 | 51 | 7 | | 63 |
| Phytoplankton | *Chrysosphaerella longispina* | Chrysophyta | 2005 | 181.75 | 31 | 1 | | 47 |
| Phytoplankton | *Closterium gracile* | Chlorophyta | 2000 | 43.41 | 47 | 1 | | 59 |
| Phytoplankton | *Closterium moniliferum* | Chlorophyta | 1995 | 8.77 | 65 | 5 | | 92 |
| Phytoplankton | *Closterium moniliferum* | Chlorophyta | 2000 | 17.36 | 64 | 5 | | 80 |
| Phytoplankton | *Closterium moniliferum* | Chlorophyta | 2001 | 72.34 | 42 | 5 | | 57 |
| Phytoplankton | *Closterium moniliferum* | Chlorophyta | 2008 | 5.49 | 59 | 5 | | 88 |
| Phytoplankton | *Closterium moniliferum* | Chlorophyta | 2010 | 37.87 | 73 | 5 | | 85 |
| Phytoplankton | *Cocconeis placentula* | Bacillariophyta | 1995 | 8.77 | 63 | 7 | | 89 |
| Phytoplankton | *Cocconeis placentula* | Bacillariophyta | 1996 | 8.77 | 53 | 7 | | 80 |
| Phytoplankton | *Cocconeis placentula* | Bacillariophyta | 1997 | 2.19 | 60 | 7 | | 100 |
| Phytoplankton | *Cocconeis placentula* | Bacillariophyta | 1999 | 10.96 | 56 | 7 | | 78 |
| Phytoplankton | *Cocconeis placentula* | Bacillariophyta | 2000 | 10.96 | 73 | 7 | | 91 |
| Phytoplankton | *Cocconeis placentula* | Bacillariophyta | 2001 | 2.92 | 68 | 7 | | 92 |
| Phytoplankton | *Cocconeis placentula* | Bacillariophyta | 2007 | 6.06 | 67 | 7 | | 92 |
| Phytoplankton | *Coelastrum astroideum* | Chlorophyta | 2000 | 27.78 | 60 | 1 | | 75 |
| Phytoplankton | *Coelastrum cambricum* | Chlorophyta | 2010 | 35.11 | 77 | 1 | | 90 |
| Phytoplankton | *Coelastrum microporum* | Chlorophyta | 2005 | 8.78 | 58 | 2 | | 88 |
| Phytoplankton | *Coelastrum microporum* | Chlorophyta | 2011 | 32.29 | 52 | 2 | | 70 |
| Phytoplankton | *Coelastrum proboscideum* | Chlorophyta | 2011 | 28.87 | 53 | 1 | | 72 |
| Phytoplankton | *Coelastrum reticulatum* | Chlorophyta | 2006 | 35.11 | 46 | 3 | | 71 |
| Phytoplankton | *Coelastrum reticulatum* | Chlorophyta | 2010 | 201.95 | 47 | 3 | | 55 |
| Phytoplankton | *Coelastrum reticulatum* | Chlorophyta | 2011 | 84.69 | 41 | 3 | | 55 |
| Phytoplankton | *Cosmarium sp. 1* | Chlorophyta | 1997 | 4.70 | 57 | 1 | | 95 |
| Phytoplankton | *Crucigenia crucifera* | Chlorophyta | 1999 | 347.25 | 21 | 3 | | 29 |
| Phytoplankton | *Crucigenia crucifera* | Chlorophyta | 2004 | 6.58 | 55 | 3 | | 87 |
| Phytoplankton | *Crucigenia crucifera* | Chlorophyta | 2008 | 65.83 | 37 | 3 | | 55 |
| Phytoplankton | *Crucigenia rectangularis* | Chlorophyta | 1996 | 5.85 | 56 | 1 | | 85 |
| Phytoplankton | *Crucigenia tetrapedia* | Chlorophyta | 1997 | 10.42 | 51 | 2 | | 85 |
| Phytoplankton | *Crucigenia tetrapedia* | Chlorophyta | 2011 | 8.47 | 67 | 2 | | 91 |
| Phytoplankton | *Cryptomonas erosa* | Cryptophyta | 1995 | 201.66 | 26 | 17 | | 37 |
| Phytoplankton | *Cryptomonas erosa* | Cryptophyta | 1996 | 312.52 | 19 | 17 | | 29 |
| Phytoplankton | *Cryptomonas erosa* | Cryptophyta | 1997 | 127.13 | 25 | 17 | | 42 |
| Phytoplankton | *Cryptomonas erosa* | Cryptophyta | 1998 | 1139.40 | 15 | 17 | | 21 |
| Phytoplankton | *Cryptomonas erosa* | Cryptophyta | 1999 | 248.03 | 25 | 17 | | 35 |
| Phytoplankton | *Cryptomonas erosa* | Cryptophyta | 2000 | 694.49 | 22 | 17 | | 28 |
| Phytoplankton | *Cryptomonas erosa* | Cryptophyta | 2001 | 156.26 | 38 | 17 | | 51 |
| Phytoplankton | *Cryptomonas erosa* | Cryptophyta | 2002 | 57.87 | 34 | 17 | | 69 |
| Phytoplankton | *Cryptomonas erosa* | Cryptophyta | 2003 | 52.67 | 26 | 17 | | 51 |
| Phytoplankton | *Cryptomonas erosa* | Cryptophyta | 2004 | 243.42 | 20 | 17 | | 32 |
| Phytoplankton | *Cryptomonas erosa* | Cryptophyta | 2005 | 318.07 | 27 | 17 | | 41 |
| Phytoplankton | *Cryptomonas erosa* | Cryptophyta | 2006 | 77.25 | 41 | 17 | | 63 |
| Phytoplankton | *Cryptomonas erosa* | Cryptophyta | 2007 | 212.05 | 36 | 17 | | 49 |
| Phytoplankton | *Cryptomonas erosa* | Cryptophyta | 2008 | 272.63 | 27 | 17 | | 40 |
| Phytoplankton | *Cryptomonas erosa* | Cryptophyta | 2009 | 308.98 | 31 | 17 | | 38 |
| Phytoplankton | *Cryptomonas erosa* | Cryptophyta | 2010 | 706.82 | 33 | 17 | | 38 |
| Phytoplankton | *Cryptomonas erosa* | Cryptophyta | 2011 | 176.44 | 30 | 17 | | 41 |
| Phytoplankton | *Cryptomonas gracilis* | Cryptophyta | 1998 | 32.55 | 46 | 9 | | 64 |
| Phytoplankton | *Cryptomonas gracilis* | Cryptophyta | 1999 | 52.09 | 40 | 9 | | 56 |
| Phytoplankton | *Cryptomonas gracilis* | Cryptophyta | 2000 | 8.68 | 77 | 9 | | 96 |
| Phytoplankton | *Cryptomonas gracilis* | Cryptophyta | 2002 | 7.23 | 42 | 9 | | 86 |
| Phytoplankton | *Cryptomonas gracilis* | Cryptophyta | 2003 | 13.17 | 42 | 9 | | 82 |
| Phytoplankton | *Cryptomonas gracilis* | Cryptophyta | 2004 | 34.08 | 37 | 9 | | 59 |
| Phytoplankton | *Cryptomonas gracilis* | Cryptophyta | 2006 | 6.06 | 61 | 9 | | 94 |
| Phytoplankton | *Cryptomonas gracilis* | Cryptophyta | 2007 | 2.73 | 71 | 9 | | 97 |
| Phytoplankton | *Cryptomonas gracilis* | Cryptophyta | 2008 | 15.15 | 49 | 9 | | 73 |
| Phytoplankton | *Cryptomonas lucens* | Cryptophyta | 1996 | 1.10 | 63 | 6 | | 95 |
| Phytoplankton | *Cryptomonas lucens* | Cryptophyta | 1998 | 13.02 | 57 | 6 | | 79 |
| Phytoplankton | *Cryptomonas lucens* | Cryptophyta | 2001 | 28.94 | 57 | 6 | | 77 |
| Phytoplankton | *Cryptomonas lucens* | Cryptophyta | 2002 | 43.41 | 36 | 6 | | 73 |
| Phytoplankton | *Cryptomonas lucens* | Cryptophyta | 2007 | 60.58 | 46 | 6 | | 63 |
| Phytoplankton | *Cryptomonas lucens* | Cryptophyta | 2011 | 2.82 | 74 | 6 | | 100 |
| Phytoplankton | *Cryptomonas ovata* | Cryptophyta | 1995 | 61.37 | 42 | 5 | | 59 |
| Phytoplankton | *Cryptomonas ovata* | Cryptophyta | 1996 | 8.77 | 50 | 5 | | 76 |
| Phytoplankton | *Cryptomonas ovata* | Cryptophyta | 1997 | 36.46 | 37 | 5 | | 62 |
| Phytoplankton | *Cryptomonas ovata* | Cryptophyta | 1998 | 488.31 | 21 | 5 | | 29 |
| Phytoplankton | *Cryptomonas ovata* | Cryptophyta | 2004 | 16.23 | 46 | 5 | | 73 |
| Phytoplankton | *Cryptomonas rostratiformis* | Cryptophyta | 1999 | 23.15 | 54 | 12 | | 75 |
| Phytoplankton | *Cryptomonas rostratiformis* | Cryptophyta | 2000 | 17.36 | 66 | 12 | | 83 |
| Phytoplankton | *Cryptomonas rostratiformis* | Cryptophyta | 2001 | 2.60 | 73 | 12 | | 99 |
| Phytoplankton | *Cryptomonas rostratiformis* | Cryptophyta | 2002 | 4.39 | 46 | 12 | | 94 |
| Phytoplankton | *Cryptomonas rostratiformis* | Cryptophyta | 2003 | 4.39 | 47 | 12 | | 92 |
| Phytoplankton | *Cryptomonas rostratiformis* | Cryptophyta | 2004 | 16.23 | 44 | 12 | | 70 |
| Phytoplankton | *Cryptomonas rostratiformis* | Cryptophyta | 2005 | 32.46 | 50 | 12 | | 76 |
| Phytoplankton | *Cryptomonas rostratiformis* | Cryptophyta | 2007 | 15.15 | 63 | 12 | | 86 |
| Phytoplankton | *Cryptomonas rostratiformis* | Cryptophyta | 2008 | 27.26 | 45 | 12 | | 67 |
| Phytoplankton | *Cryptomonas rostratiformis* | Cryptophyta | 2009 | 8.78 | 77 | 12 | | 95 |
| Phytoplankton | *Cryptomonas rostratiformis* | Cryptophyta | 2010 | 50.49 | 71 | 12 | | 83 |
| Phytoplankton | *Cryptomonas rostratiformis* | Cryptophyta | 2011 | 26.47 | 55 | 12 | | 74 |
| Phytoplankton | *Cyanocatena planctonica* | Cyanophyta | 2008 | 12319.29 | 9 | 1 | | 13 |
| Phytoplankton | *Cyanogranis ferruginea* | Cyanophyta | 2001 | 19310.24 | 7 | 5 | | 9 |
| Phytoplankton | *Cyanogranis ferruginea* | Cyanophyta | 2005 | 9855.44 | 6 | 5 | | 9 |
| Phytoplankton | *Cyanogranis ferruginea* | Cyanophyta | 2006 | 1368.81 | 20 | 5 | | 31 |
| Phytoplankton | *Cyanogranis ferruginea* | Cyanophyta | 2007 | 657.03 | 24 | 5 | | 33 |
| Phytoplankton | *Cyanogranis ferruginea* | Cyanophyta | 2008 | 30113.69 | 8 | 5 | | 12 |
| Phytoplankton | *Cyanonephron styloides* | Cyanophyta | 2011 | 635.19 | 21 | 1 | | 28 |
| Phytoplankton | *Cyclostephanos invisitatus* | Bacillariophyta | 1995 | 22322.93 | 7 | 4 | | 10 |
| Phytoplankton | *Cyclostephanos invisitatus* | Bacillariophyta | 1996 | 28335.24 | 5 | 4 | | 8 |
| Phytoplankton | *Cyclostephanos invisitatus* | Bacillariophyta | 1997 | 23612.70 | 5 | 4 | | 8 |
| Phytoplankton | *Cyclostephanos invisitatus* | Bacillariophyta | 1998 | 31252.11 | 6 | 4 | | 8 |
| Phytoplankton | *Cyclotella bodanica* | Bacillariophyta | 1995 | 104.17 | 34 | 2 | | 48 |
| Phytoplankton | *Cyclotella bodanica* | Bacillariophyta | 2000 | 8.68 | 76 | 2 | | 95 |
| Phytoplankton | *Cyclotella cf ocellata* | Bacillariophyta | 2003 | 2.02 | 50 | 1 | | 98 |
| Phytoplankton | *Cyclotella meneghiniana* | Bacillariophyta | 2000 | 86.81 | 36 | 3 | | 45 |
| Phytoplankton | *Cyclotella meneghiniana* | Bacillariophyta | 2008 | 45.44 | 40 | 3 | | 60 |
| Phytoplankton | *Cyclotella meneghiniana* | Bacillariophyta | 2011 | 16.94 | 59 | 3 | | 80 |
| Phytoplankton | *Cyclotella ocellata* | Bacillariophyta | 2000 | 6.95 | 80 | 2 | | 100 |
| Phytoplankton | *Cyclotella ocellata* | Bacillariophyta | 2008 | 37.87 | 42 | 2 | | 63 |
| Phytoplankton | *Cyclotella pseudostelligera* | Bacillariophyta | 2010 | 37.87 | 75 | 1 | | 87 |
| Phytoplankton | *Cyclotella sp. 1* | Bacillariophyta | 1998 | 31.25 | 49 | 10 | | 68 |
| Phytoplankton | *Cyclotella sp. 1* | Bacillariophyta | 1999 | 6771.29 | 10 | 10 | | 14 |
| Phytoplankton | *Cyclotella sp. 1* | Bacillariophyta | 2000 | 7604.68 | 9 | 10 | | 11 |
| Phytoplankton | *Cyclotella sp. 1* | Bacillariophyta | 2001 | 20730.56 | 6 | 10 | | 8 |
| Phytoplankton | *Cyclotella sp. 1* | Bacillariophyta | 2004 | 102.24 | 26 | 10 | | 41 |
| Phytoplankton | *Cyclotella sp. 1* | Bacillariophyta | 2005 | 38.95 | 45 | 10 | | 68 |
| Phytoplankton | *Cyclotella sp. 1* | Bacillariophyta | 2006 | 399.86 | 27 | 10 | | 42 |
| Phytoplankton | *Cyclotella sp. 1* | Bacillariophyta | 2008 | 15.15 | 51 | 10 | | 76 |
| Phytoplankton | *Cyclotella sp. 1* | Bacillariophyta | 2009 | 97.37 | 46 | 10 | | 57 |
| Phytoplankton | *Cyclotella sp. 1* | Bacillariophyta | 2011 | 8.47 | 64 | 10 | | 86 |
| Phytoplankton | *Cyclotella stelligera* | Bacillariophyta | 2009 | 2019.49 | 19 | 2 | | 23 |
| Phytoplankton | *Cyclotella stelligera* | Bacillariophyta | 2010 | 50.49 | 69 | 2 | | 80 |
| Phytoplankton | *Cylindrospermopsis raciborskii* | Cyanophyta | 1995 | 6544.04 | 14 | 8 | | 20 |
| Phytoplankton | *Cylindrospermopsis raciborskii* | Cyanophyta | 1998 | 705.35 | 18 | 8 | | 25 |
| Phytoplankton | *Cylindrospermopsis raciborskii* | Cyanophyta | 1999 | 2242.56 | 15 | 8 | | 21 |
| Phytoplankton | *Cylindrospermopsis raciborskii* | Cyanophyta | 2000 | 868.12 | 18 | 8 | | 23 |
| Phytoplankton | *Cylindrospermopsis raciborskii* | Cyanophyta | 2001 | 2491.81 | 14 | 8 | | 19 |
| Phytoplankton | *Cylindrospermopsis raciborskii* | Cyanophyta | 2006 | 545.26 | 26 | 8 | | 40 |
| Phytoplankton | *Cylindrospermopsis raciborskii* | Cyanophyta | 2007 | 1034.99 | 21 | 8 | | 29 |
| Phytoplankton | *Cylindrospermopsis raciborskii* | Cyanophyta | 2010 | 969.35 | 30 | 8 | | 35 |
| Phytoplankton | *Cymbella prostrata* | Bacillariophyta | 2011 | 3.83 | 72 | 1 | | 97 |
| Phytoplankton | *Cymbella sp. 1* | Bacillariophyta | 1998 | 5.21 | 67 | 2 | | 93 |
| Phytoplankton | *Cymbella sp. 1* | Bacillariophyta | 2006 | 4.54 | 62 | 2 | | 95 |
| Phytoplankton | *Cystomonas starrii* | Chlorophyta | 1998 | 8.77 | 59 | 1 | | 82 |
| Phytoplankton | *Dactylococcopsis irregularis* | Cyanophyta | 1995 | 4.38 | 69 | 3 | | 97 |
| Phytoplankton | *Dactylococcopsis irregularis* | Cyanophyta | 1996 | 34.73 | 40 | 3 | | 61 |
| Phytoplankton | *Dactylococcopsis irregularis* | Cyanophyta | 1998 | 13.02 | 56 | 3 | | 78 |
| Phytoplankton | *Deasonia gigantica* | Chlorophyta | 1995 | 11.58 | 59 | 9 | | 83 |
| Phytoplankton | *Deasonia gigantica* | Chlorophyta | 1996 | 8.77 | 54 | 9 | | 82 |
| Phytoplankton | *Deasonia gigantica* | Chlorophyta | 1997 | 65.11 | 33 | 9 | | 55 |
| Phytoplankton | *Deasonia gigantica* | Chlorophyta | 2003 | 18.18 | 34 | 9 | | 67 |
| Phytoplankton | *Deasonia gigantica* | Chlorophyta | 2004 | 11.36 | 53 | 9 | | 84 |
| Phytoplankton | *Deasonia gigantica* | Chlorophyta | 2005 | 0.29 | 66 | 9 | | 100 |
| Phytoplankton | *Deasonia gigantica* | Chlorophyta | 2006 | 45.44 | 44 | 9 | | 68 |
| Phytoplankton | *Deasonia gigantica* | Chlorophyta | 2007 | 0.69 | 73 | 9 | | 100 |
| Phytoplankton | *Deasonia gigantica* | Chlorophyta | 2010 | 0.88 | 86 | 9 | | 100 |
| Phytoplankton | *Desmarella sp. 1* | Chrysophyta | 1997 | 26.04 | 43 | 5 | | 72 |
| Phytoplankton | *Desmarella sp. 1* | Chrysophyta | 1998 | 162.77 | 28 | 5 | | 39 |
| Phytoplankton | *Desmarella sp. 1* | Chrysophyta | 2004 | 97.37 | 27 | 5 | | 43 |
| Phytoplankton | *Desmarella sp. 1* | Chrysophyta | 2005 | 106.02 | 35 | 5 | | 53 |
| Phytoplankton | *Desmarella sp. 1* | Chrysophyta | 2010 | 136.32 | 55 | 5 | | 64 |
| Phytoplankton | *Diatoma tenue* | Bacillariophyta | 2002 | 57.87 | 33 | 1 | | 67 |
| Phytoplankton | *Diatoma tenuis* | Bacillariophyta | 2009 | 165.40 | 36 | 1 | | 44 |
| Phytoplankton | *Dictyosphaerium pulchellum* | Chlorophyta | 1995 | 100.21 | 36 | 9 | | 51 |
| Phytoplankton | *Dictyosphaerium pulchellum* | Chlorophyta | 2000 | 138.90 | 33 | 9 | | 41 |
| Phytoplankton | *Dictyosphaerium pulchellum* | Chlorophyta | 2001 | 868.11 | 24 | 9 | | 32 |
| Phytoplankton | *Dictyosphaerium pulchellum* | Chlorophyta | 2002 | 95.49 | 24 | 9 | | 49 |
| Phytoplankton | *Dictyosphaerium pulchellum* | Chlorophyta | 2006 | 113.60 | 37 | 9 | | 57 |
| Phytoplankton | *Dictyosphaerium pulchellum* | Chlorophyta | 2008 | 1363.15 | 21 | 9 | | 31 |
| Phytoplankton | *Dictyosphaerium pulchellum* | Chlorophyta | 2009 | 140.45 | 42 | 9 | | 52 |
| Phytoplankton | *Dictyosphaerium pulchellum* | Chlorophyta | 2010 | 113.60 | 58 | 9 | | 67 |
| Phytoplankton | *Dictyosphaerium pulchellum* | Chlorophyta | 2011 | 33.88 | 47 | 9 | | 64 |
| Phytoplankton | *Dinobryon cylindricum* | Chrysophyta | 2009 | 6.06 | 80 | 1 | | 99 |
| Phytoplankton | *Dinobryon divergens* | Chrysophyta | 2003 | 2.02 | 51 | 1 | | 100 |
| Phytoplankton | *Dinobryon sertularia* | Chrysophyta | 1998 | 13.02 | 55 | 6 | | 76 |
| Phytoplankton | *Dinobryon sertularia* | Chrysophyta | 1999 | 8.77 | 58 | 6 | | 81 |
| Phytoplankton | *Dinobryon sertularia* | Chrysophyta | 2000 | 34.73 | 52 | 6 | | 65 |
| Phytoplankton | *Dinobryon sertularia* | Chrysophyta | 2004 | 11.36 | 52 | 6 | | 83 |
| Phytoplankton | *Dinobryon sertularia* | Chrysophyta | 2005 | 30.29 | 52 | 6 | | 79 |
| Phytoplankton | *Dinobryon sertularia* | Chrysophyta | 2007 | 18.18 | 61 | 6 | | 84 |
| Phytoplankton | *Dinobryon sociale* | Chrysophyta | 2009 | 18.18 | 67 | 1 | | 83 |
| Phytoplankton | *Diploneis sp. 1* | Bacillariophyta | 1996 | 1.10 | 62 | 1 | | 94 |
| Phytoplankton | *Elakatothrix gelatinosa* | Chlorophyta | 1995 | 5.85 | 66 | 2 | | 93 |
| Phytoplankton | *Elakatothrix gelatinosa* | Chlorophyta | 1999 | 86.81 | 36 | 2 | | 50 |
| Phytoplankton | *Entomoneis cf ornata* | Bacillariophyta | 2010 | 2.19 | 85 | 1 | | 99 |
| Phytoplankton | *Erkenia subaequiciliata* | Chrysophyta | 1996 | 243.07 | 20 | 16 | | 30 |
| Phytoplankton | *Erkenia subaequiciliata* | Chrysophyta | 1997 | 115.75 | 26 | 16 | | 43 |
| Phytoplankton | *Erkenia subaequiciliata* | Chrysophyta | 1998 | 555.59 | 20 | 16 | | 28 |
| Phytoplankton | *Erkenia subaequiciliata* | Chrysophyta | 1999 | 260.43 | 24 | 16 | | 33 |
| Phytoplankton | *Erkenia subaequiciliata* | Chrysophyta | 2000 | 34.73 | 53 | 16 | | 66 |
| Phytoplankton | *Erkenia subaequiciliata* | Chrysophyta | 2001 | 15.63 | 62 | 16 | | 84 |
| Phytoplankton | *Erkenia subaequiciliata* | Chrysophyta | 2002 | 136.32 | 21 | 16 | | 43 |
| Phytoplankton | *Erkenia subaequiciliata* | Chrysophyta | 2003 | 109.05 | 20 | 16 | | 39 |
| Phytoplankton | *Erkenia subaequiciliata* | Chrysophyta | 2004 | 2017.47 | 11 | 16 | | 17 |
| Phytoplankton | *Erkenia subaequiciliata* | Chrysophyta | 2005 | 340.79 | 26 | 16 | | 39 |
| Phytoplankton | *Erkenia subaequiciliata* | Chrysophyta | 2006 | 749.73 | 24 | 16 | | 37 |
| Phytoplankton | *Erkenia subaequiciliata* | Chrysophyta | 2007 | 1408.59 | 17 | 16 | | 23 |
| Phytoplankton | *Erkenia subaequiciliata* | Chrysophyta | 2008 | 454.38 | 25 | 16 | | 37 |
| Phytoplankton | *Erkenia subaequiciliata* | Chrysophyta | 2009 | 490.74 | 28 | 16 | | 35 |
| Phytoplankton | *Erkenia subaequiciliata* | Chrysophyta | 2010 | 2589.99 | 16 | 16 | | 19 |
| Phytoplankton | *Erkenia subaequiciliata* | Chrysophyta | 2011 | 502.86 | 22 | 16 | | 30 |
| Phytoplankton | *Eudorina elegans* | Chlorophyta | 2000 | 104.17 | 35 | 1 | | 44 |
| Phytoplankton | *Euglena sp. 1* | Euglenophyta | 1996 | 1.10 | 64 | 6 | | 97 |
| Phytoplankton | *Euglena sp. 1* | Bacillariophyta | 1996 | 14.62 | 47 | 1 | | 71 |
| Phytoplankton | *Euglena sp. 1* | Euglenophyta | 1997 | 13.02 | 48 | 6 | | 80 |
| Phytoplankton | *Euglena sp. 1* | Euglenophyta | 2004 | 54.53 | 33 | 6 | | 52 |
| Phytoplankton | *Euglena sp. 1* | Euglenophyta | 2005 | 35.66 | 49 | 6 | | 74 |
| Phytoplankton | *Euglena sp. 1* | Euglenophyta | 2007 | 15.15 | 65 | 6 | | 89 |
| Phytoplankton | *Euglena sp. 1* | Euglenophyta | 2008 | 3.03 | 65 | 6 | | 97 |
| Phytoplankton | *Excentrosphaera viridis* | Chlorophyta | 1998 | 32.55 | 47 | 1 | | 65 |
| Phytoplankton | *Fragilaria capucina* | Bacillariophyta | 1995 | 11.69 | 57 | 8 | | 80 |
| Phytoplankton | *Fragilaria capucina* | Bacillariophyta | 1996 | 73.06 | 27 | 8 | | 41 |
| Phytoplankton | *Fragilaria capucina* | Bacillariophyta | 1999 | 26.30 | 51 | 8 | | 71 |
| Phytoplankton | *Fragilaria capucina* | Bacillariophyta | 2002 | 482.22 | 17 | 8 | | 35 |
| Phytoplankton | *Fragilaria capucina* | Bacillariophyta | 2003 | 19.20 | 32 | 8 | | 63 |
| Phytoplankton | *Fragilaria capucina* | Bacillariophyta | 2005 | 93.47 | 38 | 8 | | 58 |
| Phytoplankton | *Fragilaria capucina* | Bacillariophyta | 2006 | 7.57 | 58 | 8 | | 89 |
| Phytoplankton | *Fragilaria capucina* | Bacillariophyta | 2008 | 7.31 | 56 | 8 | | 84 |
| Phytoplankton | *Fragilaria construens* | Bacillariophyta | 1996 | 0.97 | 65 | 8 | | 98 |
| Phytoplankton | *Fragilaria construens* | Bacillariophyta | 1999 | 87.68 | 34 | 8 | | 47 |
| Phytoplankton | *Fragilaria construens* | Bacillariophyta | 2001 | 2.60 | 70 | 8 | | 95 |
| Phytoplankton | *Fragilaria construens* | Bacillariophyta | 2002 | 90.88 | 26 | 8 | | 53 |
| Phytoplankton | *Fragilaria construens* | Bacillariophyta | 2006 | 30.29 | 47 | 8 | | 72 |
| Phytoplankton | *Fragilaria construens* | Bacillariophyta | 2007 | 339.41 | 32 | 8 | | 44 |
| Phytoplankton | *Fragilaria construens* | Bacillariophyta | 2008 | 53.01 | 39 | 8 | | 58 |
| Phytoplankton | *Fragilaria construens* | Bacillariophyta | 2010 | 120.70 | 56 | 8 | | 65 |
| Phytoplankton | *Fragilaria crotonensis* | Bacillariophyta | 1997 | 130.22 | 24 | 4 | | 40 |
| Phytoplankton | *Fragilaria crotonensis* | Bacillariophyta | 2000 | 17.36 | 63 | 4 | | 79 |
| Phytoplankton | *Fragilaria crotonensis* | Bacillariophyta | 2002 | 57.54 | 35 | 4 | | 71 |
| Phytoplankton | *Fragilaria crotonensis* | Bacillariophyta | 2007 | 90.88 | 41 | 4 | | 56 |
| Phytoplankton | *Glenodinium quadridens* | Pyrrhophyta | 1995 | 175.35 | 28 | 2 | | 39 |
| Phytoplankton | *Glenodinium quadridens* | Pyrrhophyta | 1997 | 21.92 | 45 | 2 | | 75 |
| Phytoplankton | *Gloeococcus minor* | Chlorophyta | 2011 | 127.04 | 35 | 1 | | 47 |
| Phytoplankton | *Gloeocystis vesiculosa* | Chlorophyta | 1996 | 69.45 | 30 | 4 | | 45 |
| Phytoplankton | *Gloeocystis vesiculosa* | Chlorophyta | 2000 | 34.72 | 58 | 4 | | 73 |
| Phytoplankton | *Gloeocystis vesiculosa* | Chlorophyta | 2003 | 121.17 | 19 | 4 | | 37 |
| Phytoplankton | *Gloeocystis vesiculosa* | Chlorophyta | 2011 | 20.45 | 58 | 4 | | 78 |
| Phytoplankton | *Gloeotrichia echinulata* | Cyanophyta | 2003 | 470.24 | 17 | 4 | | 33 |
| Phytoplankton | *Gloeotrichia echinulata* | Cyanophyta | 2004 | 4114.63 | 10 | 4 | | 16 |
| Phytoplankton | *Gloeotrichia echinulata* | Cyanophyta | 2006 | 6846.74 | 9 | 4 | | 14 |
| Phytoplankton | *Gloeotrichia echinulata* | Cyanophyta | 2009 | 1987.93 | 20 | 4 | | 25 |
| Phytoplankton | *Golenkinia radiata* | Chlorophyta | 2001 | 217.03 | 35 | 2 | | 47 |
| Phytoplankton | *Golenkinia radiata* | Chlorophyta | 2010 | 37.87 | 74 | 2 | | 86 |
| Phytoplankton | *Gomphonema parvulum* | Bacillariophyta | 1996 | 11.58 | 49 | 5 | | 74 |
| Phytoplankton | *Gomphonema parvulum* | Bacillariophyta | 1997 | 4.70 | 54 | 5 | | 90 |
| Phytoplankton | *Gomphonema parvulum* | Bacillariophyta | 1998 | 5.21 | 69 | 5 | | 96 |
| Phytoplankton | *Gomphonema parvulum* | Bacillariophyta | 1999 | 8.27 | 60 | 5 | | 83 |
| Phytoplankton | *Gomphonema parvulum* | Bacillariophyta | 2001 | 2.60 | 71 | 5 | | 96 |
| Phytoplankton | *Gomphosphaeria lacustris* | Cyanophyta | 1995 | 250.52 | 24 | 8 | | 34 |
| Phytoplankton | *Gomphosphaeria lacustris* | Cyanophyta | 1998 | 243.07 | 25 | 8 | | 35 |
| Phytoplankton | *Gomphosphaeria lacustris* | Cyanophyta | 2000 | 694.48 | 23 | 8 | | 29 |
| Phytoplankton | *Gomphosphaeria lacustris* | Cyanophyta | 2001 | 1736.23 | 20 | 8 | | 27 |
| Phytoplankton | *Gomphosphaeria lacustris* | Cyanophyta | 2004 | 145.40 | 24 | 8 | | 38 |
| Phytoplankton | *Gomphosphaeria lacustris* | Cyanophyta | 2005 | 908.77 | 19 | 8 | | 29 |
| Phytoplankton | *Gomphosphaeria lacustris* | Cyanophyta | 2007 | 605.85 | 26 | 8 | | 36 |
| Phytoplankton | *Gomphosphaeria lacustris* | Cyanophyta | 2010 | 908.77 | 31 | 8 | | 36 |
| Phytoplankton | *Gonium sp. 1* | Chlorophyta | 1997 | 75.15 | 32 | 1 | | 53 |
| Phytoplankton | *Gymnodinium sp. 1* | Pyrrhophyta | 1999 | 8.27 | 61 | 2 | | 85 |
| Phytoplankton | *Gymnodinium sp. 1* | Pyrrhophyta | 2009 | 18.18 | 69 | 2 | | 85 |
| Phytoplankton | *Gymnodinium sp. 2* | Pyrrhophyta | 1995 | 109.60 | 33 | 12 | | 46 |
| Phytoplankton | *Gymnodinium sp. 2* | Pyrrhophyta | 1996 | 26.30 | 42 | 12 | | 64 |
| Phytoplankton | *Gymnodinium sp. 2* | Pyrrhophyta | 1998 | 52.09 | 38 | 12 | | 53 |
| Phytoplankton | *Gymnodinium sp. 2* | Pyrrhophyta | 1999 | 4.38 | 70 | 12 | | 97 |
| Phytoplankton | *Gymnodinium sp. 2* | Pyrrhophyta | 2000 | 34.73 | 51 | 12 | | 64 |
| Phytoplankton | *Gymnodinium sp. 2* | Pyrrhophyta | 2002 | 6.06 | 45 | 12 | | 92 |
| Phytoplankton | *Gymnodinium sp. 2* | Pyrrhophyta | 2004 | 32.46 | 38 | 12 | | 60 |
| Phytoplankton | *Gymnodinium sp. 2* | Pyrrhophyta | 2005 | 15.15 | 55 | 12 | | 83 |
| Phytoplankton | *Gymnodinium sp. 2* | Pyrrhophyta | 2007 | 22.72 | 59 | 12 | | 81 |
| Phytoplankton | *Gymnodinium sp. 2* | Pyrrhophyta | 2008 | 9.09 | 52 | 12 | | 78 |
| Phytoplankton | *Gymnodinium sp. 2* | Pyrrhophyta | 2010 | 242.34 | 46 | 12 | | 53 |
| Phytoplankton | *Gymnodinium sp. 2* | Pyrrhophyta | 2011 | 132.33 | 34 | 12 | | 46 |
| Phytoplankton | *Gymnodinium sp. 3* | Pyrrhophyta | 1995 | 677.13 | 19 | 15 | | 27 |
| Phytoplankton | *Gymnodinium sp. 3* | Pyrrhophyta | 1996 | 104.17 | 25 | 15 | | 38 |
| Phytoplankton | *Gymnodinium sp. 3* | Pyrrhophyta | 1997 | 28.94 | 39 | 15 | | 65 |
| Phytoplankton | *Gymnodinium sp. 3* | Pyrrhophyta | 1998 | 20.84 | 51 | 15 | | 71 |
| Phytoplankton | *Gymnodinium sp. 3* | Pyrrhophyta | 2000 | 69.45 | 42 | 15 | | 53 |
| Phytoplankton | *Gymnodinium sp. 3* | Pyrrhophyta | 2001 | 52.09 | 51 | 15 | | 69 |
| Phytoplankton | *Gymnodinium sp. 3* | Pyrrhophyta | 2002 | 1.74 | 49 | 15 | | 100 |
| Phytoplankton | *Gymnodinium sp. 3* | Pyrrhophyta | 2003 | 18.18 | 35 | 15 | | 69 |
| Phytoplankton | *Gymnodinium sp. 3* | Pyrrhophyta | 2004 | 90.88 | 28 | 15 | | 44 |
| Phytoplankton | *Gymnodinium sp. 3* | Pyrrhophyta | 2006 | 36.35 | 45 | 15 | | 69 |
| Phytoplankton | *Gymnodinium sp. 3* | Pyrrhophyta | 2007 | 30.29 | 54 | 15 | | 74 |
| Phytoplankton | *Gymnodinium sp. 3* | Pyrrhophyta | 2008 | 9.09 | 53 | 15 | | 79 |
| Phytoplankton | *Gymnodinium sp. 3* | Pyrrhophyta | 2009 | 50.49 | 57 | 15 | | 70 |
| Phytoplankton | *Gymnodinium sp. 3* | Pyrrhophyta | 2010 | 443.03 | 37 | 15 | | 43 |
| Phytoplankton | *Gymnodinium sp. 3* | Pyrrhophyta | 2011 | 291.13 | 26 | 15 | | 35 |
| Phytoplankton | *Kephyrion gracilis* | Chrysophyta | 1999 | 5.79 | 63 | 4 | | 88 |
| Phytoplankton | *Kephyrion gracilis* | Chrysophyta | 2004 | 22.72 | 41 | 4 | | 65 |
| Phytoplankton | *Kephyrion gracilis* | Chrysophyta | 2006 | 6.06 | 59 | 4 | | 91 |
| Phytoplankton | *Kephyrion gracilis* | Chrysophyta | 2007 | 27.26 | 57 | 4 | | 78 |
| Phytoplankton | *Kephyrion planctonicum* | Chrysophyta | 2008 | 18.18 | 47 | 1 | | 70 |
| Phytoplankton | *Kephyrion sp. 5* | Chrysophyta | 1995 | 11.58 | 58 | 1 | | 82 |
| Phytoplankton | *Kirchneriella lunaris* | Chlorophyta | 2010 | 151.46 | 54 | 1 | | 63 |
| Phytoplankton | *Kirchneriella obesa* | Chlorophyta | 1997 | 26.04 | 42 | 1 | | 70 |
| Phytoplankton | *Lagerheimia ciliata* | Chlorophyta | 1997 | 13.02 | 49 | 2 | | 82 |
| Phytoplankton | *Lagerheimia ciliata* | Chlorophyta | 2009 | 50.49 | 56 | 2 | | 69 |
| Phytoplankton | *Limnothrix redekei* | Cyanophyta | 2010 | 378.65 | 38 | 1 | | 44 |
| Phytoplankton | *Lobomonas sp. 1* | Chlorophyta | 1996 | 17.36 | 46 | 5 | | 70 |
| Phytoplankton | *Lobomonas sp. 1* | Chlorophyta | 1998 | 4.34 | 72 | 5 | | 100 |
| Phytoplankton | *Lobomonas sp. 1* | Chlorophyta | 2001 | 52.09 | 48 | 5 | | 65 |
| Phytoplankton | *Lobomonas sp. 1* | Chlorophyta | 2009 | 9.09 | 75 | 5 | | 93 |
| Phytoplankton | *Lobomonas sp. 1* | Chlorophyta | 2011 | 16.94 | 61 | 5 | | 82 |
| Phytoplankton | *Lyngbya circumcreta* | Cyanophyta | 1995 | 14063.49 | 9 | 3 | | 13 |
| Phytoplankton | *Lyngbya circumcreta* | Cyanophyta | 1996 | 657.60 | 16 | 3 | | 24 |
| Phytoplankton | *Lyngbya circumcreta* | Cyanophyta | 2001 | 218.76 | 34 | 3 | | 46 |
| Phytoplankton | *Lyngbya lagerheimia* | Cyanophyta | 1996 | 315.62 | 18 | 3 | | 27 |
| Phytoplankton | *Lyngbya lagerheimia* | Cyanophyta | 2010 | 1514.62 | 19 | 3 | | 22 |
| Phytoplankton | *Lyngbya lagerheimia* | Cyanophyta | 2011 | 1058.65 | 19 | 3 | | 26 |
| Phytoplankton | *Lyngbya limnetica* | Cyanophyta | 1995 | 175.36 | 27 | 9 | | 38 |
| Phytoplankton | *Lyngbya limnetica* | Cyanophyta | 1996 | 716.03 | 15 | 9 | | 23 |
| Phytoplankton | *Lyngbya limnetica* | Cyanophyta | 2000 | 3067.29 | 14 | 9 | | 18 |
| Phytoplankton | *Lyngbya limnetica* | Cyanophyta | 2001 | 5401.60 | 11 | 9 | | 15 |
| Phytoplankton | *Lyngbya limnetica* | Cyanophyta | 2005 | 425.99 | 24 | 9 | | 36 |
| Phytoplankton | *Lyngbya limnetica* | Cyanophyta | 2007 | 1135.96 | 19 | 9 | | 26 |
| Phytoplankton | *Lyngbya limnetica* | Cyanophyta | 2009 | 1720.61 | 22 | 9 | | 27 |
| Phytoplankton | *Lyngbya limnetica* | Cyanophyta | 2010 | 192386.51 | 1 | 9 | | 1 |
| Phytoplankton | *Lyngbya limnetica* | Cyanophyta | 2011 | 2117.31 | 12 | 9 | | 16 |
| Phytoplankton | *Mallomonas akrokomas* | Chrysophyta | 1999 | 24.80 | 52 | 7 | | 72 |
| Phytoplankton | *Mallomonas akrokomas* | Chrysophyta | 2000 | 34.73 | 49 | 7 | | 61 |
| Phytoplankton | *Mallomonas akrokomas* | Chrysophyta | 2003 | 18.18 | 40 | 7 | | 78 |
| Phytoplankton | *Mallomonas akrokomas* | Chrysophyta | 2006 | 90.88 | 38 | 7 | | 58 |
| Phytoplankton | *Mallomonas akrokomas* | Chrysophyta | 2007 | 72.70 | 44 | 7 | | 60 |
| Phytoplankton | *Mallomonas akrokomas* | Chrysophyta | 2008 | 3.66 | 62 | 7 | | 93 |
| Phytoplankton | *Mallomonas akrokomas* | Chrysophyta | 2009 | 9.09 | 74 | 7 | | 91 |
| Phytoplankton | *Mallomonas caudata* | Chrysophyta | 1995 | 0.54 | 71 | 1 | | 100 |
| Phytoplankton | *Melosira varians* | Bacillariophyta | 2007 | 70.22 | 45 | 1 | | 62 |
| Phytoplankton | *Merismopedia elegans* | Cyanophyta | 1997 | 140.29 | 23 | 1 | | 38 |
| Phytoplankton | *Merismopedia minima* | Cyanophyta | 1998 | 144113.85 | 3 | 2 | | 4 |
| Phytoplankton | *Merismopedia minima* | Cyanophyta | 2001 | 31.25 | 55 | 2 | | 74 |
| Phytoplankton | *Merismopedia punctata* | Cyanophyta | 2010 | 277.68 | 44 | 1 | | 51 |
| Phytoplankton | *Merismopedia tenuissima* | Cyanophyta | 1995 | 178137.00 | 3 | 14 | | 4 |
| Phytoplankton | *Merismopedia tenuissima* | Cyanophyta | 1996 | 10547.58 | 8 | 14 | | 12 |
| Phytoplankton | *Merismopedia tenuissima* | Cyanophyta | 1997 | 104.18 | 28 | 14 | | 47 |
| Phytoplankton | *Merismopedia tenuissima* | Cyanophyta | 1999 | 27345.59 | 5 | 14 | | 7 |
| Phytoplankton | *Merismopedia tenuissima* | Cyanophyta | 2000 | 4861.44 | 12 | 14 | | 15 |
| Phytoplankton | *Merismopedia tenuissima* | Cyanophyta | 2001 | 85653.69 | 4 | 14 | | 5 |
| Phytoplankton | *Merismopedia tenuissima* | Cyanophyta | 2002 | 90283.72 | 4 | 14 | | 8 |
| Phytoplankton | *Merismopedia tenuissima* | Cyanophyta | 2003 | 6179.63 | 7 | 14 | | 14 |
| Phytoplankton | *Merismopedia tenuissima* | Cyanophyta | 2005 | 1817.54 | 15 | 14 | | 23 |
| Phytoplankton | *Merismopedia tenuissima* | Cyanophyta | 2006 | 2326.45 | 18 | 14 | | 28 |
| Phytoplankton | *Merismopedia tenuissima* | Cyanophyta | 2007 | 4038.98 | 13 | 14 | | 18 |
| Phytoplankton | *Merismopedia tenuissima* | Cyanophyta | 2009 | 7270.16 | 12 | 14 | | 15 |
| Phytoplankton | *Merismopedia tenuissima* | Cyanophyta | 2010 | 14540.31 | 11 | 14 | | 13 |
| Phytoplankton | *Merismopedia tenuissima* | Cyanophyta | 2011 | 1626.09 | 13 | 14 | | 18 |
| Phytoplankton | *Merismopedia warmingiana* | Cyanophyta | 2003 | 6785.48 | 6 | 9 | | 12 |
| Phytoplankton | *Merismopedia warmingiana* | Cyanophyta | 2004 | 290.81 | 17 | 9 | | 27 |
| Phytoplankton | *Merismopedia warmingiana* | Cyanophyta | 2005 | 8057.76 | 7 | 9 | | 11 |
| Phytoplankton | *Merismopedia warmingiana* | Cyanophyta | 2006 | 6106.93 | 10 | 9 | | 15 |
| Phytoplankton | *Merismopedia warmingiana* | Cyanophyta | 2007 | 142131.55 | 3 | 9 | | 4 |
| Phytoplankton | *Merismopedia warmingiana* | Cyanophyta | 2008 | 31100.11 | 7 | 9 | | 10 |
| Phytoplankton | *Merismopedia warmingiana* | Cyanophyta | 2009 | 25243.57 | 8 | 9 | | 10 |
| Phytoplankton | *Merismopedia warmingiana* | Cyanophyta | 2010 | 152814.64 | 2 | 9 | | 2 |
| Phytoplankton | *Merismopedia warmingiana* | Cyanophyta | 2011 | 33347.62 | 6 | 9 | | 8 |
| Phytoplankton | *Micractinium pusillum* | Chlorophyta | 1999 | 46.30 | 42 | 3 | | 58 |
| Phytoplankton | *Micractinium pusillum* | Chlorophyta | 2001 | 1851.98 | 18 | 3 | | 24 |
| Phytoplankton | *Micractinium pusillum* | Chlorophyta | 2010 | 68.16 | 65 | 3 | | 76 |
| Phytoplankton | *Microcystis aeruginosa* | Cyanophyta | 1995 | 208608.68 | 2 | 17 | | 3 |
| Phytoplankton | *Microcystis aeruginosa* | Cyanophyta | 1996 | 18543.12 | 6 | 17 | | 9 |
| Phytoplankton | *Microcystis aeruginosa* | Cyanophyta | 1997 | 76368.88 | 2 | 17 | | 3 |
| Phytoplankton | *Microcystis aeruginosa* | Cyanophyta | 1998 | 130662.75 | 4 | 17 | | 6 |
| Phytoplankton | *Microcystis aeruginosa* | Cyanophyta | 1999 | 87870.53 | 4 | 17 | | 6 |
| Phytoplankton | *Microcystis aeruginosa* | Cyanophyta | 2000 | 55765.09 | 4 | 17 | | 5 |
| Phytoplankton | *Microcystis aeruginosa* | Cyanophyta | 2001 | 68754.63 | 5 | 17 | | 7 |
| Phytoplankton | *Microcystis aeruginosa* | Cyanophyta | 2002 | 14974.97 | 5 | 17 | | 10 |
| Phytoplankton | *Microcystis aeruginosa* | Cyanophyta | 2003 | 28853.42 | 3 | 17 | | 6 |
| Phytoplankton | *Microcystis aeruginosa* | Cyanophyta | 2004 | 31436.78 | 3 | 17 | | 5 |
| Phytoplankton | *Microcystis aeruginosa* | Cyanophyta | 2005 | 18932.70 | 5 | 17 | | 8 |
| Phytoplankton | *Microcystis aeruginosa* | Cyanophyta | 2006 | 7951.73 | 7 | 17 | | 11 |
| Phytoplankton | *Microcystis aeruginosa* | Cyanophyta | 2007 | 47551.36 | 6 | 17 | | 8 |
| Phytoplankton | *Microcystis aeruginosa* | Cyanophyta | 2008 | 88352.59 | 3 | 17 | | 4 |
| Phytoplankton | *Microcystis aeruginosa* | Cyanophyta | 2009 | 61432.82 | 4 | 17 | | 5 |
| Phytoplankton | *Microcystis aeruginosa* | Cyanophyta | 2010 | 20819.08 | 8 | 17 | | 9 |
| Phytoplankton | *Microcystis aeruginosa* | Cyanophyta | 2011 | 13656.65 | 8 | 17 | | 11 |
| Phytoplankton | *Microcystis flos-aquae* | Cyanophyta | 2005 | 658.34 | 20 | 2 | | 30 |
| Phytoplankton | *Microcystis flos-aquae* | Cyanophyta | 2006 | 3017.39 | 15 | 2 | | 23 |
| Phytoplankton | *Microcystis novacekii* | Cyanophyta | 2007 | 158.00 | 37 | 1 | | 51 |
| Phytoplankton | *Microcystis wesenbergii* | Cyanophyta | 2006 | 877.79 | 22 | 3 | | 34 |
| Phytoplankton | *Microcystis wesenbergii* | Cyanophyta | 2008 | 822.93 | 23 | 3 | | 34 |
| Phytoplankton | *Microcystis wesenbergii* | Cyanophyta | 2010 | 1329.08 | 24 | 3 | | 28 |
| Phytoplankton | *Monodus sp. 1* | Xanthophyta | 2010 | 3.03 | 84 | 1 | | 98 |
| Phytoplankton | *Monomastix astigmata* | Chlorophyta | 1997 | 286.48 | 21 | 13 | | 35 |
| Phytoplankton | *Monomastix astigmata* | Chlorophyta | 1998 | 32.55 | 45 | 13 | | 63 |
| Phytoplankton | *Monomastix astigmata* | Chlorophyta | 1999 | 43.41 | 44 | 13 | | 61 |
| Phytoplankton | *Monomastix astigmata* | Chlorophyta | 2000 | 43.41 | 44 | 13 | | 55 |
| Phytoplankton | *Monomastix astigmata* | Chlorophyta | 2001 | 5.21 | 66 | 13 | | 89 |
| Phytoplankton | *Monomastix astigmata* | Chlorophyta | 2003 | 3189.78 | 11 | 13 | | 22 |
| Phytoplankton | *Monomastix astigmata* | Chlorophyta | 2005 | 9.09 | 57 | 13 | | 86 |
| Phytoplankton | *Monomastix astigmata* | Chlorophyta | 2006 | 3226.13 | 14 | 13 | | 22 |
| Phytoplankton | *Monomastix astigmata* | Chlorophyta | 2007 | 327.16 | 33 | 13 | | 45 |
| Phytoplankton | *Monomastix astigmata* | Chlorophyta | 2008 | 545.26 | 24 | 13 | | 36 |
| Phytoplankton | *Monomastix astigmata* | Chlorophyta | 2009 | 2921.04 | 16 | 13 | | 20 |
| Phytoplankton | *Monomastix astigmata* | Chlorophyta | 2010 | 50.49 | 67 | 13 | | 78 |
| Phytoplankton | *Monomastix astigmata* | Chlorophyta | 2011 | 8.47 | 65 | 13 | | 88 |
| Phytoplankton | *Monomastix minuta* | Chlorophyta | 2006 | 3.03 | 65 | 2 | | 100 |
| Phytoplankton | *Monomastix minuta* | Chlorophyta | 2011 | 16.94 | 60 | 2 | | 81 |
| Phytoplankton | *Monoraphidium arcuatum* | Chlorophyta | 1995 | 225.71 | 25 | 16 | | 35 |
| Phytoplankton | *Monoraphidium arcuatum* | Chlorophyta | 1996 | 17.54 | 44 | 16 | | 67 |
| Phytoplankton | *Monoraphidium arcuatum* | Chlorophyta | 1997 | 13.02 | 47 | 16 | | 78 |
| Phytoplankton | *Monoraphidium arcuatum* | Chlorophyta | 1998 | 8.68 | 63 | 16 | | 88 |
| Phytoplankton | *Monoraphidium arcuatum* | Chlorophyta | 1999 | 173.62 | 31 | 16 | | 43 |
| Phytoplankton | *Monoraphidium arcuatum* | Chlorophyta | 2000 | 86.81 | 37 | 16 | | 46 |
| Phytoplankton | *Monoraphidium arcuatum* | Chlorophyta | 2001 | 72.34 | 44 | 16 | | 59 |
| Phytoplankton | *Monoraphidium arcuatum* | Chlorophyta | 2003 | 18.18 | 37 | 16 | | 73 |
| Phytoplankton | *Monoraphidium arcuatum* | Chlorophyta | 2004 | 11.36 | 50 | 16 | | 79 |
| Phytoplankton | *Monoraphidium arcuatum* | Chlorophyta | 2005 | 6.49 | 59 | 16 | | 89 |
| Phytoplankton | *Monoraphidium arcuatum* | Chlorophyta | 2006 | 12.12 | 54 | 16 | | 83 |
| Phytoplankton | *Monoraphidium arcuatum* | Chlorophyta | 2007 | 28.40 | 55 | 16 | | 75 |
| Phytoplankton | *Monoraphidium arcuatum* | Chlorophyta | 2008 | 56.80 | 38 | 16 | | 57 |
| Phytoplankton | *Monoraphidium arcuatum* | Chlorophyta | 2009 | 151.46 | 40 | 16 | | 49 |
| Phytoplankton | *Monoraphidium arcuatum* | Chlorophyta | 2010 | 318.07 | 43 | 16 | | 50 |
| Phytoplankton | *Monoraphidium arcuatum* | Chlorophyta | 2011 | 8.47 | 66 | 16 | | 89 |
| Phytoplankton | *Monoraphidium capricornutum* | Chlorophyta | 1995 | 416.70 | 20 | 17 | | 28 |
| Phytoplankton | *Monoraphidium capricornutum* | Chlorophyta | 1996 | 138.90 | 24 | 17 | | 36 |
| Phytoplankton | *Monoraphidium capricornutum* | Chlorophyta | 1997 | 104.17 | 29 | 17 | | 48 |
| Phytoplankton | *Monoraphidium capricornutum* | Chlorophyta | 1998 | 86.81 | 33 | 17 | | 46 |
| Phytoplankton | *Monoraphidium capricornutum* | Chlorophyta | 1999 | 434.06 | 20 | 17 | | 28 |
| Phytoplankton | *Monoraphidium capricornutum* | Chlorophyta | 2000 | 173.62 | 29 | 17 | | 36 |
| Phytoplankton | *Monoraphidium capricornutum* | Chlorophyta | 2001 | 506.40 | 28 | 17 | | 38 |
| Phytoplankton | *Monoraphidium capricornutum* | Chlorophyta | 2002 | 759.60 | 15 | 17 | | 31 |
| Phytoplankton | *Monoraphidium capricornutum* | Chlorophyta | 2003 | 60.58 | 24 | 17 | | 47 |
| Phytoplankton | *Monoraphidium capricornutum* | Chlorophyta | 2004 | 54.53 | 34 | 17 | | 54 |
| Phytoplankton | *Monoraphidium capricornutum* | Chlorophyta | 2005 | 151.46 | 33 | 17 | | 50 |
| Phytoplankton | *Monoraphidium capricornutum* | Chlorophyta | 2006 | 90.88 | 40 | 17 | | 62 |
| Phytoplankton | *Monoraphidium capricornutum* | Chlorophyta | 2007 | 353.41 | 30 | 17 | | 41 |
| Phytoplankton | *Monoraphidium capricornutum* | Chlorophyta | 2008 | 37.87 | 41 | 17 | | 61 |
| Phytoplankton | *Monoraphidium capricornutum* | Chlorophyta | 2009 | 50.49 | 55 | 17 | | 68 |
| Phytoplankton | *Monoraphidium capricornutum* | Chlorophyta | 2010 | 1060.23 | 26 | 17 | | 30 |
| Phytoplankton | *Monoraphidium capricornutum* | Chlorophyta | 2011 | 264.66 | 27 | 17 | | 36 |
| Phytoplankton | *Monosiga sp. 1* | Chrysophyta | 1998 | 2552.26 | 9 | 1 | | 13 |
| Phytoplankton | *Myxobaktron salinum* | Cyanophyta | 2010 | 201.95 | 48 | 1 | | 56 |
| Phytoplankton | *Navicula bacillum* | Bacillariophyta | 1999 | 4.38 | 72 | 1 | | 100 |
| Phytoplankton | *Navicula capitata* | Bacillariophyta | 1999 | 8.27 | 59 | 1 | | 82 |
| Phytoplankton | *Navicula cf. lacunolaciniata* | Bacillariophyta | 1999 | 5.79 | 62 | 1 | | 86 |
| Phytoplankton | *Navicula cryptocephala* | Bacillariophyta | 1996 | 5.85 | 59 | 7 | | 89 |
| Phytoplankton | *Navicula cryptocephala* | Bacillariophyta | 1997 | 28.94 | 40 | 7 | | 67 |
| Phytoplankton | *Navicula cryptocephala* | Bacillariophyta | 1998 | 8.68 | 61 | 7 | | 85 |
| Phytoplankton | *Navicula cryptocephala* | Bacillariophyta | 1999 | 10.96 | 57 | 7 | | 79 |
| Phytoplankton | *Navicula cryptocephala* | Bacillariophyta | 2000 | 17.36 | 68 | 7 | | 85 |
| Phytoplankton | *Navicula cryptocephala* | Bacillariophyta | 2002 | 2.92 | 48 | 7 | | 98 |
| Phytoplankton | *Navicula cryptocephala* | Bacillariophyta | 2006 | 7.57 | 57 | 7 | | 88 |
| Phytoplankton | *Navicula gregaria* | Bacillariophyta | 2009 | 4.39 | 81 | 1 | | 100 |
| Phytoplankton | *Navicula rhynchocephala* | Bacillariophyta | 1995 | 8.77 | 61 | 1 | | 86 |
| Phytoplankton | *Navicula scutelloides* | Bacillariophyta | 1999 | 24.80 | 53 | 1 | | 74 |
| Phytoplankton | *Navicula sp. 1* | Bacillariophyta | 1995 | 8.77 | 62 | 1 | | 87 |
| Phytoplankton | *Navicula tripunctata* | Bacillariophyta | 2005 | 0.29 | 64 | 1 | | 97 |
| Phytoplankton | *Navicula viridula* | Bacillariophyta | 2008 | 3.66 | 61 | 1 | | 91 |
| Phytoplankton | *Nephroselmis sp. 1* | Chlorophyta | 2009 | 6.49 | 79 | 1 | | 98 |
| Phytoplankton | *Nitzschia acicularis* | Bacillariophyta | 1995 | 23.15 | 49 | 11 | | 69 |
| Phytoplankton | *Nitzschia acicularis* | Bacillariophyta | 1996 | 5.85 | 58 | 11 | | 88 |
| Phytoplankton | *Nitzschia acicularis* | Bacillariophyta | 1997 | 4.38 | 58 | 11 | | 97 |
| Phytoplankton | *Nitzschia acicularis* | Bacillariophyta | 1998 | 32.55 | 48 | 11 | | 67 |
| Phytoplankton | *Nitzschia acicularis* | Bacillariophyta | 1999 | 86.81 | 37 | 11 | | 51 |
| Phytoplankton | *Nitzschia acicularis* | Bacillariophyta | 2000 | 34.73 | 50 | 11 | | 63 |
| Phytoplankton | *Nitzschia acicularis* | Bacillariophyta | 2004 | 4.39 | 57 | 11 | | 90 |
| Phytoplankton | *Nitzschia acicularis* | Bacillariophyta | 2008 | 6.06 | 58 | 11 | | 87 |
| Phytoplankton | *Nitzschia acicularis* | Bacillariophyta | 2009 | 9.09 | 76 | 11 | | 94 |
| Phytoplankton | *Nitzschia acicularis* | Bacillariophyta | 2010 | 34.08 | 78 | 11 | | 91 |
| Phytoplankton | *Nitzschia acicularis* | Bacillariophyta | 2011 | 132.33 | 33 | 11 | | 45 |
| Phytoplankton | *Nitzschia amphibia* | Bacillariophyta | 2002 | 6.06 | 44 | 1 | | 90 |
| Phytoplankton | *Nitzschia gracilis* | Bacillariophyta | 1999 | 5.79 | 64 | 4 | | 89 |
| Phytoplankton | *Nitzschia gracilis* | Bacillariophyta | 2000 | 8.68 | 75 | 4 | | 94 |
| Phytoplankton | *Nitzschia gracilis* | Bacillariophyta | 2002 | 12.40 | 39 | 4 | | 80 |
| Phytoplankton | *Nitzschia gracilis* | Bacillariophyta | 2007 | 50.49 | 50 | 4 | | 68 |
| Phytoplankton | *Nitzschia linearis* | Bacillariophyta | 2005 | 3.14 | 61 | 1 | | 92 |
| Phytoplankton | *Nitzschia palea* | Bacillariophyta | 1995 | 2.92 | 70 | 11 | | 99 |
| Phytoplankton | *Nitzschia palea* | Bacillariophyta | 1999 | 130.22 | 33 | 11 | | 46 |
| Phytoplankton | *Nitzschia palea* | Bacillariophyta | 2000 | 86.81 | 38 | 11 | | 48 |
| Phytoplankton | *Nitzschia palea* | Bacillariophyta | 2001 | 28.94 | 58 | 11 | | 78 |
| Phytoplankton | *Nitzschia palea* | Bacillariophyta | 2002 | 86.81 | 28 | 11 | | 57 |
| Phytoplankton | *Nitzschia palea* | Bacillariophyta | 2005 | 38.40 | 46 | 11 | | 70 |
| Phytoplankton | *Nitzschia palea* | Bacillariophyta | 2006 | 27.26 | 48 | 11 | | 74 |
| Phytoplankton | *Nitzschia palea* | Bacillariophyta | 2007 | 340.79 | 31 | 11 | | 42 |
| Phytoplankton | *Nitzschia palea* | Bacillariophyta | 2008 | 100.97 | 32 | 11 | | 48 |
| Phytoplankton | *Nitzschia palea* | Bacillariophyta | 2009 | 353.42 | 30 | 11 | | 37 |
| Phytoplankton | *Nitzschia palea* | Bacillariophyta | 2010 | 68.16 | 64 | 11 | | 74 |
| Phytoplankton | *Nitzschia perminuta* | Bacillariophyta | 1995 | 17.36 | 51 | 3 | | 72 |
| Phytoplankton | *Nitzschia perminuta* | Bacillariophyta | 1998 | 91.15 | 32 | 3 | | 44 |
| Phytoplankton | *Nitzschia perminuta* | Bacillariophyta | 1999 | 17.36 | 55 | 3 | | 76 |
| Phytoplankton | *Nitzschia sigma* | Bacillariophyta | 2004 | 16.23 | 47 | 1 | | 75 |
| Phytoplankton | *Nitzschia sigmoidea* | Bacillariophyta | 2000 | 17.36 | 70 | 1 | | 88 |
| Phytoplankton | *Nitzschia subacicularis* | Bacillariophyta | 2009 | 36.35 | 60 | 1 | | 74 |
| Phytoplankton | *Ochromonas sp. 1* | Chrysophyta | 1998 | 43.41 | 42 | 3 | | 58 |
| Phytoplankton | *Ochromonas sp. 1* | Chrysophyta | 1999 | 34.73 | 49 | 3 | | 68 |
| Phytoplankton | *Ochromonas sp. 1* | Chrysophyta | 2003 | 18.18 | 39 | 3 | | 76 |
| Phytoplankton | *Oocystis borgei* | Chlorophyta | 1997 | 4.70 | 55 | 3 | | 92 |
| Phytoplankton | *Oocystis borgei* | Chlorophyta | 1998 | 5.21 | 65 | 3 | | 90 |
| Phytoplankton | *Oocystis borgei* | Chlorophyta | 2000 | 34.72 | 57 | 3 | | 71 |
| Phytoplankton | *Oocystis lacustris* | Chlorophyta | 2000 | 34.72 | 59 | 5 | | 74 |
| Phytoplankton | *Oocystis lacustris* | Chlorophyta | 2007 | 2.27 | 72 | 5 | | 99 |
| Phytoplankton | *Oocystis lacustris* | Chlorophyta | 2008 | 6.06 | 57 | 5 | | 85 |
| Phytoplankton | *Oocystis lacustris* | Chlorophyta | 2010 | 18.18 | 80 | 5 | | 93 |
| Phytoplankton | *Oocystis lacustris* | Chlorophyta | 2011 | 5.65 | 69 | 5 | | 93 |
| Phytoplankton | *Oocystis parva* | Chlorophyta | 1995 | 277.83 | 23 | 15 | | 32 |
| Phytoplankton | *Oocystis parva* | Chlorophyta | 1996 | 208.35 | 21 | 15 | | 32 |
| Phytoplankton | *Oocystis parva* | Chlorophyta | 1997 | 2.60 | 59 | 15 | | 98 |
| Phytoplankton | *Oocystis parva* | Chlorophyta | 1998 | 83.34 | 35 | 15 | | 49 |
| Phytoplankton | *Oocystis parva* | Chlorophyta | 1999 | 43.84 | 43 | 15 | | 60 |
| Phytoplankton | *Oocystis parva* | Chlorophyta | 2000 | 138.90 | 32 | 15 | | 40 |
| Phytoplankton | *Oocystis parva* | Chlorophyta | 2001 | 289.37 | 31 | 15 | | 42 |
| Phytoplankton | *Oocystis parva* | Chlorophyta | 2002 | 909.45 | 13 | 15 | | 27 |
| Phytoplankton | *Oocystis parva* | Chlorophyta | 2003 | 79.00 | 23 | 15 | | 45 |
| Phytoplankton | *Oocystis parva* | Chlorophyta | 2004 | 218.10 | 21 | 15 | | 33 |
| Phytoplankton | *Oocystis parva* | Chlorophyta | 2006 | 181.75 | 34 | 15 | | 52 |
| Phytoplankton | *Oocystis parva* | Chlorophyta | 2008 | 201.95 | 29 | 15 | | 43 |
| Phytoplankton | *Oocystis parva* | Chlorophyta | 2009 | 302.92 | 32 | 15 | | 40 |
| Phytoplankton | *Oocystis parva* | Chlorophyta | 2010 | 454.38 | 36 | 15 | | 42 |
| Phytoplankton | *Oocystis parva* | Chlorophyta | 2011 | 142.28 | 31 | 15 | | 42 |
| Phytoplankton | *Oocystis pusilla* | Chlorophyta | 1996 | 416.70 | 17 | 3 | | 26 |
| Phytoplankton | *Oocystis pusilla* | Chlorophyta | 2001 | 43.41 | 52 | 3 | | 70 |
| Phytoplankton | *Oocystis pusilla* | Chlorophyta | 2011 | 101.63 | 38 | 3 | | 51 |
| Phytoplankton | *Oscillatoria amphibia* | Cyanophyta | 2000 | 260.44 | 27 | 1 | | 34 |
| Phytoplankton | *Oscillatoria angustissima* | Cyanophyta | 1998 | 937.55 | 16 | 1 | | 22 |
| Phytoplankton | *Oscillatoria chlorina* | Cyanophyta | 1998 | 694.48 | 19 | 3 | | 26 |
| Phytoplankton | *Oscillatoria chlorina* | Cyanophyta | 1999 | 3906.54 | 11 | 3 | | 15 |
| Phytoplankton | *Oscillatoria chlorina* | Cyanophyta | 2000 | 20371.93 | 8 | 3 | | 10 |
| Phytoplankton | *Oscillatoria limnetica* | Cyanophyta | 2007 | 880.37 | 22 | 1 | | 30 |
| Phytoplankton | *Oscillatoria minnesotensis* | Cyanophyta | 2001 | 723.43 | 27 | 1 | | 36 |
| Phytoplankton | *Oscillatoria tenuis* | Cyanophyta | 1995 | 39.46 | 45 | 2 | | 63 |
| Phytoplankton | *Oscillatoria tenuis* | Cyanophyta | 1998 | 46.88 | 41 | 2 | | 57 |
| Phytoplankton | *Pandorina morum* | Chlorophyta | 1996 | 8.77 | 52 | 4 | | 79 |
| Phytoplankton | *Pandorina morum* | Chlorophyta | 1997 | 328.79 | 18 | 4 | | 30 |
| Phytoplankton | *Pandorina morum* | Chlorophyta | 2000 | 173.62 | 31 | 4 | | 39 |
| Phytoplankton | *Pandorina morum* | Chlorophyta | 2009 | 65.83 | 49 | 4 | | 60 |
| Phytoplankton | *Pediastrum boryanum* | Chlorophyta | 2000 | 17.36 | 72 | 4 | | 90 |
| Phytoplankton | *Pediastrum boryanum* | Chlorophyta | 2001 | 160.74 | 37 | 4 | | 50 |
| Phytoplankton | *Pediastrum boryanum* | Chlorophyta | 2010 | 1211.69 | 25 | 4 | | 29 |
| Phytoplankton | *Pediastrum boryanum* | Chlorophyta | 2011 | 28.23 | 54 | 4 | | 73 |
| Phytoplankton | *Pediastrum duplex* | Chlorophyta | 1995 | 70.14 | 39 | 3 | | 55 |
| Phytoplankton | *Pediastrum duplex* | Chlorophyta | 1997 | 23.15 | 44 | 3 | | 73 |
| Phytoplankton | *Pediastrum duplex* | Chlorophyta | 2009 | 64.91 | 50 | 3 | | 62 |
| Phytoplankton | *Pediastrum simplex* | Chlorophyta | 1996 | 39.46 | 35 | 3 | | 53 |
| Phytoplankton | *Pediastrum simplex* | Chlorophyta | 2004 | 11.70 | 49 | 3 | | 78 |
| Phytoplankton | *Pediastrum simplex* | Chlorophyta | 2007 | 4.54 | 68 | 3 | | 93 |
| Phytoplankton | *Pedinomonas minutissima* | Chlorophyta | 2009 | 25.96 | 64 | 1 | | 79 |
| Phytoplankton | *Peridinium cinctum* | Pyrrhophyta | 2004 | 1.10 | 63 | 2 | | 100 |
| Phytoplankton | *Peridinium cinctum* | Pyrrhophyta | 2006 | 3.03 | 64 | 2 | | 98 |
| Phytoplankton | *Peridinium polonicum* | Pyrrhophyta | 2008 | 3.66 | 64 | 1 | | 96 |
| Phytoplankton | *Peridinium umbonatum* | Pyrrhophyta | 1996 | 104.17 | 26 | 9 | | 39 |
| Phytoplankton | *Peridinium umbonatum* | Pyrrhophyta | 1997 | 173.62 | 22 | 9 | | 37 |
| Phytoplankton | *Peridinium umbonatum* | Pyrrhophyta | 1998 | 82.47 | 36 | 9 | | 50 |
| Phytoplankton | *Peridinium umbonatum* | Pyrrhophyta | 2000 | 43.41 | 46 | 9 | | 58 |
| Phytoplankton | *Peridinium umbonatum* | Pyrrhophyta | 2001 | 52.09 | 47 | 9 | | 64 |
| Phytoplankton | *Peridinium umbonatum* | Pyrrhophyta | 2004 | 64.91 | 30 | 9 | | 48 |
| Phytoplankton | *Peridinium umbonatum* | Pyrrhophyta | 2005 | 19.47 | 54 | 9 | | 82 |
| Phytoplankton | *Peridinium umbonatum* | Pyrrhophyta | 2007 | 15.15 | 64 | 9 | | 88 |
| Phytoplankton | *Peridinium umbonatum* | Pyrrhophyta | 2010 | 151.46 | 50 | 9 | | 58 |
| Phytoplankton | *Phacotus lendneri* | Chlorophyta | 1995 | 23.15 | 48 | 7 | | 68 |
| Phytoplankton | *Phacotus lendneri* | Chlorophyta | 1997 | 4.70 | 56 | 7 | | 93 |
| Phytoplankton | *Phacotus lendneri* | Chlorophyta | 1998 | 69.45 | 37 | 7 | | 51 |
| Phytoplankton | *Phacotus lendneri* | Chlorophyta | 2000 | 130.22 | 34 | 7 | | 43 |
| Phytoplankton | *Phacotus lendneri* | Chlorophyta | 2002 | 96.46 | 23 | 7 | | 47 |
| Phytoplankton | *Phacotus lendneri* | Chlorophyta | 2003 | 18.18 | 38 | 7 | | 75 |
| Phytoplankton | *Phacotus lendneri* | Chlorophyta | 2008 | 3.03 | 66 | 7 | | 99 |
| Phytoplankton | *Phacus sp. 1* | Euglenophyta | 1997 | 8.77 | 53 | 1 | | 88 |
| Phytoplankton | *Phormidium sp. 1* | Cyanophyta | 2008 | 3407.89 | 15 | 1 | | 22 |
| Phytoplankton | *Planktothrix agardhii* | Cyanophyta | 1999 | 3906.51 | 12 | 6 | | 17 |
| Phytoplankton | *Planktothrix agardhii* | Cyanophyta | 2000 | 127393.85 | 2 | 6 | | 3 |
| Phytoplankton | *Planktothrix agardhii* | Cyanophyta | 2001 | 133689.63 | 2 | 6 | | 3 |
| Phytoplankton | *Planktothrix agardhii* | Cyanophyta | 2009 | 76673.45 | 3 | 6 | | 4 |
| Phytoplankton | *Planktothrix agardhii* | Cyanophyta | 2010 | 13774.35 | 12 | 6 | | 14 |
| Phytoplankton | *Planktothrix agardhii* | Cyanophyta | 2011 | 38.35 | 46 | 6 | | 62 |
| Phytoplankton | *Polygoniochloris circularis* | Chrysophyta | 1995 | 17.36 | 53 | 1 | | 75 |
| Phytoplankton | *Pseudanabaena biceps* | Cyanophyta | 1997 | 16.44 | 46 | 1 | | 77 |
| Phytoplankton | *Pseudanabaena franquetii* | Cyanophyta | 2003 | 415.44 | 18 | 1 | | 35 |
| Phytoplankton | *Pseudanabaena galeata* | Cyanophyta | 2001 | 78.12 | 40 | 3 | | 54 |
| Phytoplankton | *Pseudanabaena galeata* | Cyanophyta | 2006 | 18.18 | 50 | 3 | | 77 |
| Phytoplankton | *Pseudanabaena galeata* | Cyanophyta | 2009 | 151.46 | 37 | 3 | | 46 |
| Phytoplankton | *Pseudanabaena limnetica* | Cyanophyta | 1995 | 14857.82 | 8 | 17 | | 11 |
| Phytoplankton | *Pseudanabaena limnetica* | Cyanophyta | 1996 | 28458.52 | 4 | 17 | | 6 |
| Phytoplankton | *Pseudanabaena limnetica* | Cyanophyta | 1997 | 2025.60 | 10 | 17 | | 17 |
| Phytoplankton | *Pseudanabaena limnetica* | Cyanophyta | 1998 | 1810.38 | 10 | 17 | | 14 |
| Phytoplankton | *Pseudanabaena limnetica* | Cyanophyta | 1999 | 17213.83 | 6 | 17 | | 8 |
| Phytoplankton | *Pseudanabaena limnetica* | Cyanophyta | 2000 | 23813.45 | 7 | 17 | | 9 |
| Phytoplankton | *Pseudanabaena limnetica* | Cyanophyta | 2001 | 14890.54 | 9 | 17 | | 12 |
| Phytoplankton | *Pseudanabaena limnetica* | Cyanophyta | 2002 | 1929.14 | 11 | 17 | | 22 |
| Phytoplankton | *Pseudanabaena limnetica* | Cyanophyta | 2003 | 3489.67 | 10 | 17 | | 20 |
| Phytoplankton | *Pseudanabaena limnetica* | Cyanophyta | 2004 | 12063.92 | 5 | 17 | | 8 |
| Phytoplankton | *Pseudanabaena limnetica* | Cyanophyta | 2005 | 3276.61 | 11 | 17 | | 17 |
| Phytoplankton | *Pseudanabaena limnetica* | Cyanophyta | 2006 | 2714.20 | 17 | 17 | | 26 |
| Phytoplankton | *Pseudanabaena limnetica* | Cyanophyta | 2007 | 4165.19 | 12 | 17 | | 16 |
| Phytoplankton | *Pseudanabaena limnetica* | Cyanophyta | 2008 | 6815.77 | 12 | 17 | | 18 |
| Phytoplankton | *Pseudanabaena limnetica* | Cyanophyta | 2009 | 34457.51 | 6 | 17 | | 7 |
| Phytoplankton | *Pseudanabaena limnetica* | Cyanophyta | 2010 | 19826.32 | 9 | 17 | | 10 |
| Phytoplankton | *Pseudanabaena limnetica* | Cyanophyta | 2011 | 3089.85 | 11 | 17 | | 15 |
| Phytoplankton | *Pseudanabaena mucicola* | Cyanophyta | 1995 | 60.77 | 43 | 17 | | 61 |
| Phytoplankton | *Pseudanabaena mucicola* | Cyanophyta | 1996 | 6003.43 | 9 | 17 | | 14 |
| Phytoplankton | *Pseudanabaena mucicola* | Cyanophyta | 1997 | 11402.36 | 6 | 17 | | 10 |
| Phytoplankton | *Pseudanabaena mucicola* | Cyanophyta | 1998 | 1504.16 | 14 | 17 | | 19 |
| Phytoplankton | *Pseudanabaena mucicola* | Cyanophyta | 1999 | 11621.88 | 8 | 17 | | 11 |
| Phytoplankton | *Pseudanabaena mucicola* | Cyanophyta | 2000 | 798.66 | 20 | 17 | | 25 |
| Phytoplankton | *Pseudanabaena mucicola* | Cyanophyta | 2001 | 2314.97 | 15 | 17 | | 20 |
| Phytoplankton | *Pseudanabaena mucicola* | Cyanophyta | 2002 | 3515.86 | 9 | 17 | | 18 |
| Phytoplankton | *Pseudanabaena mucicola* | Cyanophyta | 2003 | 1325.30 | 13 | 17 | | 25 |
| Phytoplankton | *Pseudanabaena mucicola* | Cyanophyta | 2004 | 6664.24 | 6 | 17 | | 10 |
| Phytoplankton | *Pseudanabaena mucicola* | Cyanophyta | 2005 | 6361.39 | 9 | 17 | | 14 |
| Phytoplankton | *Pseudanabaena mucicola* | Cyanophyta | 2006 | 5755.54 | 11 | 17 | | 17 |
| Phytoplankton | *Pseudanabaena mucicola* | Cyanophyta | 2007 | 11359.62 | 7 | 17 | | 10 |
| Phytoplankton | *Pseudanabaena mucicola* | Cyanophyta | 2008 | 10791.53 | 10 | 17 | | 15 |
| Phytoplankton | *Pseudanabaena mucicola* | Cyanophyta | 2009 | 8945.70 | 10 | 17 | | 12 |
| Phytoplankton | *Pseudanabaena mucicola* | Cyanophyta | 2010 | 1467.66 | 21 | 17 | | 24 |
| Phytoplankton | *Pseudanabaena mucicola* | Cyanophyta | 2011 | 1422.83 | 16 | 17 | | 22 |
| Phytoplankton | *Pyramichlamys dissecta* | Chlorophyta | 2004 | 11.36 | 51 | 5 | | 81 |
| Phytoplankton | *Pyramichlamys dissecta* | Chlorophyta | 2008 | 5.49 | 60 | 5 | | 90 |
| Phytoplankton | *Pyramichlamys dissecta* | Chlorophyta | 2009 | 75.73 | 47 | 5 | | 58 |
| Phytoplankton | *Pyramichlamys dissecta* | Chlorophyta | 2010 | 113.60 | 57 | 5 | | 66 |
| Phytoplankton | *Pyramichlamys dissecta* | Chlorophyta | 2011 | 4.09 | 71 | 5 | | 96 |
| Phytoplankton | *Quadrigula lacustris* | Chlorophyta | 1995 | 4.38 | 68 | 15 | | 96 |
| Phytoplankton | *Quadrigula lacustris* | Chlorophyta | 1996 | 11.69 | 48 | 15 | | 73 |
| Phytoplankton | *Quadrigula lacustris* | Chlorophyta | 1997 | 78.13 | 31 | 15 | | 52 |
| Phytoplankton | *Quadrigula lacustris* | Chlorophyta | 1998 | 8.68 | 60 | 15 | | 83 |
| Phytoplankton | *Quadrigula lacustris* | Chlorophyta | 1999 | 49.61 | 41 | 15 | | 57 |
| Phytoplankton | *Quadrigula lacustris* | Chlorophyta | 2001 | 6.94 | 65 | 15 | | 88 |
| Phytoplankton | *Quadrigula lacustris* | Chlorophyta | 2002 | 69.45 | 32 | 15 | | 65 |
| Phytoplankton | *Quadrigula lacustris* | Chlorophyta | 2003 | 708.84 | 14 | 15 | | 27 |
| Phytoplankton | *Quadrigula lacustris* | Chlorophyta | 2004 | 36.35 | 35 | 15 | | 56 |
| Phytoplankton | *Quadrigula lacustris* | Chlorophyta | 2005 | 75.73 | 39 | 15 | | 59 |
| Phytoplankton | *Quadrigula lacustris* | Chlorophyta | 2006 | 45.44 | 43 | 15 | | 66 |
| Phytoplankton | *Quadrigula lacustris* | Chlorophyta | 2007 | 56.80 | 49 | 15 | | 67 |
| Phytoplankton | *Quadrigula lacustris* | Chlorophyta | 2009 | 50.49 | 52 | 15 | | 64 |
| Phytoplankton | *Quadrigula lacustris* | Chlorophyta | 2010 | 75.73 | 62 | 15 | | 72 |
| Phytoplankton | *Quadrigula lacustris* | Chlorophyta | 2011 | 52.93 | 44 | 15 | | 59 |
| Phytoplankton | *Rhabdoderma lineare* | Cyanophyta | 2006 | 7156.56 | 8 | 4 | | 12 |
| Phytoplankton | *Rhabdoderma lineare* | Cyanophyta | 2007 | 1211.69 | 18 | 4 | | 25 |
| Phytoplankton | *Rhabdoderma lineare* | Cyanophyta | 2009 | 757.31 | 26 | 4 | | 32 |
| Phytoplankton | *Rhabdoderma lineare* | Cyanophyta | 2010 | 545.26 | 35 | 4 | | 41 |
| Phytoplankton | *Rhodomonas minuta* | Cryptophyta | 1995 | 1828.83 | 16 | 17 | | 23 |
| Phytoplankton | *Rhodomonas minuta* | Cryptophyta | 1996 | 1145.91 | 12 | 17 | | 18 |
| Phytoplankton | *Rhodomonas minuta* | Cryptophyta | 1997 | 1927.21 | 11 | 17 | | 18 |
| Phytoplankton | *Rhodomonas minuta* | Cryptophyta | 1998 | 6250.42 | 8 | 17 | | 11 |
| Phytoplankton | *Rhodomonas minuta* | Cryptophyta | 1999 | 824.71 | 18 | 17 | | 25 |
| Phytoplankton | *Rhodomonas minuta* | Cryptophyta | 2000 | 2569.62 | 15 | 17 | | 19 |
| Phytoplankton | *Rhodomonas minuta* | Cryptophyta | 2001 | 1996.66 | 17 | 17 | | 23 |
| Phytoplankton | *Rhodomonas minuta* | Cryptophyta | 2002 | 933.22 | 12 | 17 | | 24 |
| Phytoplankton | *Rhodomonas minuta* | Cryptophyta | 2003 | 590.70 | 16 | 17 | | 31 |
| Phytoplankton | *Rhodomonas minuta* | Cryptophyta | 2004 | 872.42 | 13 | 17 | | 21 |
| Phytoplankton | *Rhodomonas minuta* | Cryptophyta | 2005 | 567.98 | 21 | 17 | | 32 |
| Phytoplankton | *Rhodomonas minuta* | Cryptophyta | 2006 | 1249.56 | 21 | 17 | | 32 |
| Phytoplankton | *Rhodomonas minuta* | Cryptophyta | 2007 | 1135.96 | 20 | 17 | | 27 |
| Phytoplankton | *Rhodomonas minuta* | Cryptophyta | 2008 | 1590.35 | 18 | 17 | | 27 |
| Phytoplankton | *Rhodomonas minuta* | Cryptophyta | 2009 | 908.77 | 24 | 17 | | 30 |
| Phytoplankton | *Rhodomonas minuta* | Cryptophyta | 2010 | 1716.56 | 17 | 17 | | 20 |
| Phytoplankton | *Rhodomonas minuta* | Cryptophyta | 2011 | 1296.85 | 17 | 17 | | 23 |
| Phytoplankton | *Rhoicosphenia curvata* | Bacillariophyta | 1999 | 5.79 | 68 | 2 | | 94 |
| Phytoplankton | *Rhoicosphenia curvata* | Bacillariophyta | 2001 | 19.29 | 60 | 2 | | 81 |
| Phytoplankton | *Romeria okensis* | Cyanophyta | 2010 | 363.51 | 39 | 1 | | 45 |
| Phytoplankton | *Scenedesmus abundans* | Chlorophyta | 2001 | 231.50 | 33 | 7 | | 45 |
| Phytoplankton | *Scenedesmus abundans* | Chlorophyta | 2004 | 218.10 | 22 | 7 | | 35 |
| Phytoplankton | *Scenedesmus abundans* | Chlorophyta | 2007 | 121.17 | 39 | 7 | | 53 |
| Phytoplankton | *Scenedesmus abundans* | Chlorophyta | 2008 | 151.46 | 31 | 7 | | 46 |
| Phytoplankton | *Scenedesmus abundans* | Chlorophyta | 2009 | 201.95 | 34 | 7 | | 42 |
| Phytoplankton | *Scenedesmus abundans* | Chlorophyta | 2010 | 100.97 | 59 | 7 | | 69 |
| Phytoplankton | *Scenedesmus abundans* | Chlorophyta | 2011 | 42.35 | 45 | 7 | | 61 |
| Phytoplankton | *Scenedesmus acutus* | Chlorophyta | 2004 | 32.46 | 39 | 2 | | 62 |
| Phytoplankton | *Scenedesmus acutus* | Chlorophyta | 2007 | 75.73 | 43 | 2 | | 59 |
| Phytoplankton | *Scenedesmus armatus* | Chlorophyta | 1998 | 15.63 | 54 | 1 | | 75 |
| Phytoplankton | *Scenedesmus bicaudatus* | Chlorophyta | 1996 | 35.07 | 36 | 2 | | 55 |
| Phytoplankton | *Scenedesmus bicaudatus* | Chlorophyta | 2009 | 29.26 | 62 | 2 | | 77 |
| Phytoplankton | *Scenedesmus bijuga* | Chlorophyta | 1995 | 69.45 | 40 | 11 | | 56 |
| Phytoplankton | *Scenedesmus bijuga* | Chlorophyta | 1996 | 69.45 | 28 | 11 | | 42 |
| Phytoplankton | *Scenedesmus bijuga* | Chlorophyta | 1997 | 52.09 | 36 | 11 | | 60 |
| Phytoplankton | *Scenedesmus bijuga* | Chlorophyta | 1998 | 52.08 | 40 | 11 | | 56 |
| Phytoplankton | *Scenedesmus bijuga* | Chlorophyta | 1999 | 86.81 | 35 | 11 | | 49 |
| Phytoplankton | *Scenedesmus bijuga* | Chlorophyta | 2000 | 17.54 | 61 | 11 | | 76 |
| Phytoplankton | *Scenedesmus bijuga* | Chlorophyta | 2001 | 3.47 | 67 | 11 | | 91 |
| Phytoplankton | *Scenedesmus bijuga* | Chlorophyta | 2003 | 30.29 | 29 | 11 | | 57 |
| Phytoplankton | *Scenedesmus bijuga* | Chlorophyta | 2004 | 36.35 | 36 | 11 | | 57 |
| Phytoplankton | *Scenedesmus bijuga* | Chlorophyta | 2007 | 60.58 | 47 | 11 | | 64 |
| Phytoplankton | *Scenedesmus bijuga* | Chlorophyta | 2009 | 72.70 | 48 | 11 | | 59 |
| Phytoplankton | *Scenedesmus dimorphus* | Chlorophyta | 1995 | 9.68 | 60 | 4 | | 85 |
| Phytoplankton | *Scenedesmus dimorphus* | Chlorophyta | 1999 | 347.24 | 22 | 4 | | 31 |
| Phytoplankton | *Scenedesmus dimorphus* | Chlorophyta | 2000 | 34.72 | 56 | 4 | | 70 |
| Phytoplankton | *Scenedesmus dimorphus* | Chlorophyta | 2001 | 1.74 | 74 | 4 | | 100 |
| Phytoplankton | *Scenedesmus incrassatulus* | Chlorophyta | 1999 | 173.62 | 29 | 1 | | 40 |
| Phytoplankton | *Scenedesmus intermedius* | Chlorophyta | 1999 | 173.62 | 30 | 1 | | 42 |
| Phytoplankton | *Scenedesmus longus* | Chlorophyta | 1998 | 115.75 | 31 | 1 | | 43 |
| Phytoplankton | *Scenedesmus opoliensis* | Chlorophyta | 2001 | 10.42 | 63 | 5 | | 85 |
| Phytoplankton | *Scenedesmus opoliensis* | Chlorophyta | 2005 | 25.96 | 53 | 5 | | 80 |
| Phytoplankton | *Scenedesmus opoliensis* | Chlorophyta | 2008 | 90.88 | 33 | 5 | | 49 |
| Phytoplankton | *Scenedesmus opoliensis* | Chlorophyta | 2009 | 16.46 | 70 | 5 | | 86 |
| Phytoplankton | *Scenedesmus opoliensis* | Chlorophyta | 2011 | 33.88 | 51 | 5 | | 69 |
| Phytoplankton | *Scenedesmus quadricauda* | Chlorophyta | 1995 | 8.77 | 64 | 11 | | 90 |
| Phytoplankton | *Scenedesmus quadricauda* | Chlorophyta | 1996 | 2.92 | 61 | 11 | | 92 |
| Phytoplankton | *Scenedesmus quadricauda* | Chlorophyta | 1997 | 115.75 | 27 | 11 | | 45 |
| Phytoplankton | *Scenedesmus quadricauda* | Chlorophyta | 1999 | 217.07 | 27 | 11 | | 38 |
| Phytoplankton | *Scenedesmus quadricauda* | Chlorophyta | 2000 | 78.13 | 39 | 11 | | 49 |
| Phytoplankton | *Scenedesmus quadricauda* | Chlorophyta | 2001 | 20.83 | 59 | 11 | | 80 |
| Phytoplankton | *Scenedesmus quadricauda* | Chlorophyta | 2005 | 56.80 | 42 | 11 | | 64 |
| Phytoplankton | *Scenedesmus quadricauda* | Chlorophyta | 2006 | 75.73 | 42 | 11 | | 65 |
| Phytoplankton | *Scenedesmus quadricauda* | Chlorophyta | 2007 | 28.40 | 56 | 11 | | 77 |
| Phytoplankton | *Scenedesmus quadricauda* | Chlorophyta | 2010 | 100.97 | 60 | 11 | | 70 |
| Phytoplankton | *Scenedesmus quadricauda* | Chlorophyta | 2011 | 16.94 | 62 | 11 | | 84 |
| Phytoplankton | *Scenedesmus serratus* | Chlorophyta | 1995 | 23.38 | 47 | 8 | | 66 |
| Phytoplankton | *Scenedesmus serratus* | Chlorophyta | 2000 | 34.72 | 55 | 8 | | 69 |
| Phytoplankton | *Scenedesmus serratus* | Chlorophyta | 2001 | 62.01 | 46 | 8 | | 62 |
| Phytoplankton | *Scenedesmus serratus* | Chlorophyta | 2005 | 56.80 | 41 | 8 | | 62 |
| Phytoplankton | *Scenedesmus serratus* | Chlorophyta | 2007 | 45.44 | 51 | 8 | | 70 |
| Phytoplankton | *Scenedesmus serratus* | Chlorophyta | 2009 | 36.35 | 59 | 8 | | 73 |
| Phytoplankton | *Scenedesmus serratus* | Chlorophyta | 2010 | 151.46 | 53 | 8 | | 62 |
| Phytoplankton | *Scenedesmus serratus* | Chlorophyta | 2011 | 135.51 | 32 | 8 | | 43 |
| Phytoplankton | *Scenedesmus sp. 2* | Chlorophyta | 1996 | 69.45 | 29 | 2 | | 44 |
| Phytoplankton | *Scenedesmus sp. 2* | Chlorophyta | 1997 | 8.77 | 52 | 2 | | 87 |
| Phytoplankton | *Schizochlamys compacta* | Chlorophyta | 1995 | 8286.23 | 12 | 4 | | 17 |
| Phytoplankton | *Schizochlamys compacta* | Chlorophyta | 1996 | 2943.75 | 11 | 4 | | 17 |
| Phytoplankton | *Schizochlamys compacta* | Chlorophyta | 1997 | 2968.94 | 9 | 4 | | 15 |
| Phytoplankton | *Schizochlamys compacta* | Chlorophyta | 1998 | 83.34 | 34 | 4 | | 47 |
| Phytoplankton | *Schroederia judayi* | Chlorophyta | 1995 | 946.24 | 17 | 17 | | 24 |
| Phytoplankton | *Schroederia judayi* | Chlorophyta | 1996 | 1076.46 | 13 | 17 | | 20 |
| Phytoplankton | *Schroederia judayi* | Chlorophyta | 1997 | 416.70 | 16 | 17 | | 27 |
| Phytoplankton | *Schroederia judayi* | Chlorophyta | 1998 | 121.54 | 30 | 17 | | 42 |
| Phytoplankton | *Schroederia judayi* | Chlorophyta | 1999 | 831.94 | 17 | 17 | | 24 |
| Phytoplankton | *Schroederia judayi* | Chlorophyta | 2000 | 395.86 | 25 | 17 | | 31 |
| Phytoplankton | *Schroederia judayi* | Chlorophyta | 2001 | 729.22 | 26 | 17 | | 35 |
| Phytoplankton | *Schroederia judayi* | Chlorophyta | 2002 | 372.05 | 18 | 17 | | 37 |
| Phytoplankton | *Schroederia judayi* | Chlorophyta | 2003 | 18.18 | 36 | 17 | | 71 |
| Phytoplankton | *Schroederia judayi* | Chlorophyta | 2004 | 599.79 | 14 | 17 | | 22 |
| Phytoplankton | *Schroederia judayi* | Chlorophyta | 2005 | 199.93 | 30 | 17 | | 45 |
| Phytoplankton | *Schroederia judayi* | Chlorophyta | 2006 | 272.63 | 28 | 17 | | 43 |
| Phytoplankton | *Schroederia judayi* | Chlorophyta | 2007 | 227.19 | 35 | 17 | | 48 |
| Phytoplankton | *Schroederia judayi* | Chlorophyta | 2008 | 227.19 | 28 | 17 | | 42 |
| Phytoplankton | *Schroederia judayi* | Chlorophyta | 2009 | 584.21 | 27 | 17 | | 33 |
| Phytoplankton | *Schroederia judayi* | Chlorophyta | 2010 | 348.36 | 41 | 17 | | 48 |
| Phytoplankton | *Schroederia judayi* | Chlorophyta | 2011 | 203.26 | 29 | 17 | | 39 |
| Phytoplankton | *Schroederia setigera* | Chlorophyta | 1996 | 4.38 | 60 | 6 | | 91 |
| Phytoplankton | *Schroederia setigera* | Chlorophyta | 2004 | 4.39 | 59 | 6 | | 94 |
| Phytoplankton | *Schroederia setigera* | Chlorophyta | 2007 | 22.72 | 58 | 6 | | 79 |
| Phytoplankton | *Schroederia setigera* | Chlorophyta | 2008 | 3.66 | 63 | 6 | | 94 |
| Phytoplankton | *Schroederia setigera* | Chlorophyta | 2009 | 12.98 | 73 | 6 | | 90 |
| Phytoplankton | *Schroederia setigera* | Chlorophyta | 2011 | 4.09 | 70 | 6 | | 95 |
| Phytoplankton | *Selenastrum minutum* | Chlorophyta | 1996 | 34.73 | 39 | 5 | | 59 |
| Phytoplankton | *Selenastrum minutum* | Chlorophyta | 2000 | 43.41 | 45 | 5 | | 56 |
| Phytoplankton | *Selenastrum minutum* | Chlorophyta | 2009 | 12.98 | 72 | 5 | | 89 |
| Phytoplankton | *Selenastrum minutum* | Chlorophyta | 2010 | 22.72 | 79 | 5 | | 92 |
| Phytoplankton | *Selenastrum minutum* | Chlorophyta | 2011 | 33.88 | 48 | 5 | | 65 |
| Phytoplankton | *Skeletonema potamos* | Bacillariophyta | 2010 | 60.58 | 66 | 1 | | 77 |
| Phytoplankton | *Snowella litoralis* | Cyanophyta | 2006 | 254.46 | 30 | 1 | | 46 |
| Phytoplankton | *Sphaerocystis schroeteri* | Chlorophyta | 1995 | 170.96 | 29 | 16 | | 41 |
| Phytoplankton | *Sphaerocystis schroeteri* | Chlorophyta | 1997 | 6380.64 | 7 | 16 | | 12 |
| Phytoplankton | *Sphaerocystis schroeteri* | Chlorophyta | 1998 | 364.60 | 22 | 16 | | 31 |
| Phytoplankton | *Sphaerocystis schroeteri* | Chlorophyta | 1999 | 1805.67 | 16 | 16 | | 22 |
| Phytoplankton | *Sphaerocystis schroeteri* | Chlorophyta | 2000 | 841.73 | 19 | 16 | | 24 |
| Phytoplankton | *Sphaerocystis schroeteri* | Chlorophyta | 2001 | 69.45 | 45 | 16 | | 61 |
| Phytoplankton | *Sphaerocystis schroeteri* | Chlorophyta | 2002 | 2083.47 | 10 | 16 | | 20 |
| Phytoplankton | *Sphaerocystis schroeteri* | Chlorophyta | 2003 | 1363.15 | 12 | 16 | | 24 |
| Phytoplankton | *Sphaerocystis schroeteri* | Chlorophyta | 2004 | 279.79 | 18 | 16 | | 29 |
| Phytoplankton | *Sphaerocystis schroeteri* | Chlorophyta | 2005 | 1090.52 | 18 | 16 | | 27 |
| Phytoplankton | *Sphaerocystis schroeteri* | Chlorophyta | 2006 | 2953.50 | 16 | 16 | | 25 |
| Phytoplankton | *Sphaerocystis schroeteri* | Chlorophyta | 2007 | 1635.79 | 16 | 16 | | 22 |
| Phytoplankton | *Sphaerocystis schroeteri* | Chlorophyta | 2008 | 363.51 | 26 | 16 | | 39 |
| Phytoplankton | *Sphaerocystis schroeteri* | Chlorophyta | 2009 | 109.05 | 44 | 16 | | 54 |
| Phytoplankton | *Sphaerocystis schroeteri* | Chlorophyta | 2010 | 7224.72 | 13 | 16 | | 15 |
| Phytoplankton | *Sphaerocystis schroeteri* | Chlorophyta | 2011 | 406.52 | 24 | 16 | | 32 |
| Phytoplankton | *Spirogyra sp. 1* | Chlorophyta | 2005 | 1.76 | 62 | 1 | | 94 |
| Phytoplankton | *Staurastrum sp. 1* | Chlorophyta | 2003 | 2.19 | 48 | 1 | | 94 |
| Phytoplankton | *Stephanodiscus hantzschii* | Bacillariophyta | 1995 | 868.11 | 18 | 11 | | 25 |
| Phytoplankton | *Stephanodiscus hantzschii* | Bacillariophyta | 1997 | 87.68 | 30 | 11 | | 50 |
| Phytoplankton | *Stephanodiscus hantzschii* | Bacillariophyta | 1998 | 1562.61 | 13 | 11 | | 18 |
| Phytoplankton | *Stephanodiscus hantzschii* | Bacillariophyta | 1999 | 33.07 | 50 | 11 | | 69 |
| Phytoplankton | *Stephanodiscus hantzschii* | Bacillariophyta | 2000 | 243.07 | 28 | 11 | | 35 |
| Phytoplankton | *Stephanodiscus hantzschii* | Bacillariophyta | 2001 | 38.20 | 53 | 11 | | 72 |
| Phytoplankton | *Stephanodiscus hantzschii* | Bacillariophyta | 2002 | 86.81 | 30 | 11 | | 61 |
| Phytoplankton | *Stephanodiscus hantzschii* | Bacillariophyta | 2005 | 0.59 | 63 | 11 | | 95 |
| Phytoplankton | *Stephanodiscus hantzschii* | Bacillariophyta | 2008 | 68.16 | 36 | 11 | | 54 |
| Phytoplankton | *Stephanodiscus hantzschii* | Bacillariophyta | 2009 | 100.97 | 45 | 11 | | 56 |
| Phytoplankton | *Stephanodiscus hantzschii* | Bacillariophyta | 2010 | 333.22 | 42 | 11 | | 49 |
| Phytoplankton | *Stephanodiscus medius* | Bacillariophyta | 2007 | 4.39 | 69 | 4 | | 95 |
| Phytoplankton | *Stephanodiscus medius* | Bacillariophyta | 2008 | 18.18 | 46 | 4 | | 69 |
| Phytoplankton | *Stephanodiscus medius* | Bacillariophyta | 2009 | 50.49 | 53 | 4 | | 65 |
| Phytoplankton | *Stephanodiscus medius* | Bacillariophyta | 2011 | 33.88 | 50 | 4 | | 68 |
| Phytoplankton | *Stephanodiscus minutulus* | Bacillariophyta | 1995 | 2222.37 | 15 | 1 | | 21 |
| Phytoplankton | *Stephanodiscus niagarae* | Bacillariophyta | 1995 | 93.52 | 37 | 12 | | 52 |
| Phytoplankton | *Stephanodiscus niagarae* | Bacillariophyta | 1996 | 49.68 | 31 | 12 | | 47 |
| Phytoplankton | *Stephanodiscus niagarae* | Bacillariophyta | 1997 | 57.87 | 35 | 12 | | 58 |
| Phytoplankton | *Stephanodiscus niagarae* | Bacillariophyta | 1998 | 41.67 | 43 | 12 | | 60 |
| Phytoplankton | *Stephanodiscus niagarae* | Bacillariophyta | 1999 | 65.11 | 39 | 12 | | 54 |
| Phytoplankton | *Stephanodiscus niagarae* | Bacillariophyta | 2000 | 17.54 | 62 | 12 | | 78 |
| Phytoplankton | *Stephanodiscus niagarae* | Bacillariophyta | 2001 | 6.94 | 64 | 12 | | 86 |
| Phytoplankton | *Stephanodiscus niagarae* | Bacillariophyta | 2002 | 8.22 | 40 | 12 | | 82 |
| Phytoplankton | *Stephanodiscus niagarae* | Bacillariophyta | 2003 | 4.54 | 46 | 12 | | 90 |
| Phytoplankton | *Stephanodiscus niagarae* | Bacillariophyta | 2009 | 6.49 | 78 | 12 | | 96 |
| Phytoplankton | *Stephanodiscus niagarae* | Bacillariophyta | 2010 | 45.44 | 72 | 12 | | 84 |
| Phytoplankton | *Stephanodiscus niagarae* | Bacillariophyta | 2011 | 237.14 | 28 | 12 | | 38 |
| Phytoplankton | *Stephanodiscus parvus* | Bacillariophyta | 2001 | 19.10 | 61 | 10 | | 82 |
| Phytoplankton | *Stephanodiscus parvus* | Bacillariophyta | 2002 | 13151.93 | 6 | 10 | | 12 |
| Phytoplankton | *Stephanodiscus parvus* | Bacillariophyta | 2003 | 5943.35 | 8 | 10 | | 16 |
| Phytoplankton | *Stephanodiscus parvus* | Bacillariophyta | 2004 | 5939.46 | 8 | 10 | | 13 |
| Phytoplankton | *Stephanodiscus parvus* | Bacillariophyta | 2005 | 3771.39 | 10 | 10 | | 15 |
| Phytoplankton | *Stephanodiscus parvus* | Bacillariophyta | 2007 | 9905.59 | 8 | 10 | | 11 |
| Phytoplankton | *Stephanodiscus parvus* | Bacillariophyta | 2008 | 72019.98 | 4 | 10 | | 6 |
| Phytoplankton | *Stephanodiscus parvus* | Bacillariophyta | 2009 | 2290.10 | 17 | 10 | | 21 |
| Phytoplankton | *Stephanodiscus parvus* | Bacillariophyta | 2010 | 30898.16 | 6 | 10 | | 7 |
| Phytoplankton | *Stephanodiscus parvus* | Bacillariophyta | 2011 | 14080.11 | 7 | 10 | | 9 |
| Phytoplankton | *Stichococcus bacillaris* | Chlorophyta | 2011 | 105.87 | 37 | 1 | | 50 |
| Phytoplankton | *Surirella sp. 1* | Bacillariophyta | 1998 | 5.21 | 66 | 1 | | 92 |
| Phytoplankton | *Synechococcus elongatus* | Cyanophyta | 1997 | 321.84 | 19 | 9 | | 32 |
| Phytoplankton | *Synechococcus elongatus* | Cyanophyta | 2004 | 15.15 | 48 | 9 | | 76 |
| Phytoplankton | *Synechococcus elongatus* | Cyanophyta | 2005 | 2726.31 | 13 | 9 | | 20 |
| Phytoplankton | *Synechococcus elongatus* | Cyanophyta | 2006 | 4907.36 | 13 | 9 | | 20 |
| Phytoplankton | *Synechococcus elongatus* | Cyanophyta | 2007 | 408.95 | 28 | 9 | | 38 |
| Phytoplankton | *Synechococcus elongatus* | Cyanophyta | 2008 | 1363.15 | 20 | 9 | | 30 |
| Phytoplankton | *Synechococcus elongatus* | Cyanophyta | 2009 | 151.46 | 39 | 9 | | 48 |
| Phytoplankton | *Synechococcus elongatus* | Cyanophyta | 2010 | 272.63 | 45 | 9 | | 52 |
| Phytoplankton | *Synechococcus elongatus* | Cyanophyta | 2011 | 1101.00 | 18 | 9 | | 24 |
| Phytoplankton | *Synechococcus leopoliensis* | Cyanophyta | 2007 | 2053.22 | 14 | 2 | | 19 |
| Phytoplankton | *Synechococcus leopoliensis* | Cyanophyta | 2010 | 353.41 | 40 | 2 | | 47 |
| Phytoplankton | *Synechococcus sp. 1* | Cyanophyta | 1999 | 16091.87 | 7 | 13 | | 10 |
| Phytoplankton | *Synechococcus sp. 1* | Cyanophyta | 2000 | 32988.33 | 6 | 13 | | 8 |
| Phytoplankton | *Synechococcus sp. 1* | Cyanophyta | 2001 | 16091.87 | 8 | 13 | | 11 |
| Phytoplankton | *Synechococcus sp. 1* | Cyanophyta | 2002 | 148849.80 | 3 | 13 | | 6 |
| Phytoplankton | *Synechococcus sp. 1* | Cyanophyta | 2003 | 19505.55 | 5 | 13 | | 10 |
| Phytoplankton | *Synechococcus sp. 1* | Cyanophyta | 2004 | 21353.44 | 4 | 13 | | 6 |
| Phytoplankton | *Synechococcus sp. 1* | Cyanophyta | 2005 | 34494.02 | 4 | 13 | | 6 |
| Phytoplankton | *Synechococcus sp. 1* | Cyanophyta | 2006 | 26691.81 | 4 | 13 | | 6 |
| Phytoplankton | *Synechococcus sp. 1* | Cyanophyta | 2007 | 62965.28 | 4 | 13 | | 5 |
| Phytoplankton | *Synechococcus sp. 1* | Cyanophyta | 2008 | 45170.75 | 6 | 13 | | 9 |
| Phytoplankton | *Synechococcus sp. 1* | Cyanophyta | 2009 | 21353.44 | 9 | 13 | | 11 |
| Phytoplankton | *Synechococcus sp. 1* | Cyanophyta | 2010 | 24638.59 | 7 | 13 | | 8 |
| Phytoplankton | *Synechococcus sp. 1* | Cyanophyta | 2011 | 75901.59 | 4 | 13 | | 5 |
| Phytoplankton | *Synechocystis sp. 1* | Cyanophyta | 2011 | 6453.56 | 9 | 1 | | 12 |
| Phytoplankton | *Synedra arcus* | Bacillariophyta | 2010 | 13.17 | 81 | 1 | | 94 |
| Phytoplankton | *Synedra cyclopum* | Bacillariophyta | 1995 | 87.68 | 38 | 6 | | 54 |
| Phytoplankton | *Synedra cyclopum* | Bacillariophyta | 2003 | 45.44 | 28 | 6 | | 55 |
| Phytoplankton | *Synedra cyclopum* | Bacillariophyta | 2004 | 58.15 | 32 | 6 | | 51 |
| Phytoplankton | *Synedra cyclopum* | Bacillariophyta | 2005 | 235.91 | 29 | 6 | | 44 |
| Phytoplankton | *Synedra cyclopum* | Bacillariophyta | 2006 | 17.56 | 51 | 6 | | 78 |
| Phytoplankton | *Synedra cyclopum* | Bacillariophyta | 2007 | 7.57 | 66 | 6 | | 90 |
| Phytoplankton | *Synedra delicatissima* | Bacillariophyta | 2007 | 4.39 | 70 | 1 | | 96 |
| Phytoplankton | *Synedra filiformis* | Bacillariophyta | 1996 | 5.85 | 57 | 15 | | 86 |
| Phytoplankton | *Synedra filiformis* | Bacillariophyta | 1997 | 11.58 | 50 | 15 | | 83 |
| Phytoplankton | *Synedra filiformis* | Bacillariophyta | 1998 | 13.02 | 58 | 15 | | 81 |
| Phytoplankton | *Synedra filiformis* | Bacillariophyta | 1999 | 86.81 | 38 | 15 | | 53 |
| Phytoplankton | *Synedra filiformis* | Bacillariophyta | 2000 | 17.36 | 67 | 15 | | 84 |
| Phytoplankton | *Synedra filiformis* | Bacillariophyta | 2001 | 2.60 | 69 | 15 | | 93 |
| Phytoplankton | *Synedra filiformis* | Bacillariophyta | 2002 | 43.41 | 37 | 15 | | 76 |
| Phytoplankton | *Synedra filiformis* | Bacillariophyta | 2004 | 3.14 | 61 | 15 | | 97 |
| Phytoplankton | *Synedra filiformis* | Bacillariophyta | 2005 | 0.29 | 65 | 15 | | 98 |
| Phytoplankton | *Synedra filiformis* | Bacillariophyta | 2006 | 145.40 | 36 | 15 | | 55 |
| Phytoplankton | *Synedra filiformis* | Bacillariophyta | 2007 | 60.58 | 48 | 15 | | 66 |
| Phytoplankton | *Synedra filiformis* | Bacillariophyta | 2008 | 37.87 | 43 | 15 | | 64 |
| Phytoplankton | *Synedra filiformis* | Bacillariophyta | 2009 | 36.35 | 61 | 15 | | 75 |
| Phytoplankton | *Synedra filiformis* | Bacillariophyta | 2010 | 68.16 | 63 | 15 | | 73 |
| Phytoplankton | *Synedra filiformis* | Bacillariophyta | 2011 | 25.41 | 57 | 15 | | 77 |
| Phytoplankton | *Synedra nana* | Bacillariophyta | 1999 | 5.79 | 66 | 1 | | 92 |
| Phytoplankton | *Synedra ulna* | Bacillariophyta | 2011 | 3.61 | 73 | 1 | | 99 |
| Phytoplankton | *Synura uvella/sphagnicola* | Chrysophyta | 1996 | 0.66 | 66 | 7 | | 100 |
| Phytoplankton | *Synura uvella/sphagnicola* | Chrysophyta | 1998 | 4.34 | 70 | 7 | | 97 |
| Phytoplankton | *Synura uvella/sphagnicola* | Chrysophyta | 2000 | 173.62 | 30 | 7 | | 38 |
| Phytoplankton | *Synura uvella/sphagnicola* | Chrysophyta | 2003 | 18.18 | 33 | 7 | | 65 |
| Phytoplankton | *Synura uvella/sphagnicola* | Chrysophyta | 2005 | 151.46 | 32 | 7 | | 48 |
| Phytoplankton | *Synura uvella/sphagnicola* | Chrysophyta | 2008 | 15.15 | 50 | 7 | | 75 |
| Phytoplankton | *Synura uvella/sphagnicola* | Chrysophyta | 2009 | 45.44 | 58 | 7 | | 72 |
| Phytoplankton | *Tetracystis pulchra* | Chlorophyta | 1996 | 169.51 | 23 | 2 | | 35 |
| Phytoplankton | *Tetracystis pulchra* | Chlorophyta | 2002 | 198.43 | 20 | 2 | | 41 |
| Phytoplankton | *Tetraedron caudatum* | Chlorophyta | 2000 | 8.68 | 74 | 2 | | 93 |
| Phytoplankton | *Tetraedron caudatum* | Chlorophyta | 2011 | 7.22 | 68 | 2 | | 92 |
| Phytoplankton | *Tetraedron minimum* | Chlorophyta | 1998 | 20.84 | 50 | 2 | | 69 |
| Phytoplankton | *Tetraedron minimum* | Chlorophyta | 2002 | 108.51 | 22 | 2 | | 45 |
| Phytoplankton | *Tetraedron muticum* | Chlorophyta | 1999 | 43.41 | 45 | 2 | | 63 |
| Phytoplankton | *Tetraedron muticum* | Chlorophyta | 2006 | 7.57 | 56 | 2 | | 86 |
| Phytoplankton | *Tetrastrum staurogeniaeforme* | Chlorophyta | 2004 | 8.78 | 54 | 6 | | 86 |
| Phytoplankton | *Tetrastrum staurogeniaeforme* | Chlorophyta | 2007 | 113.60 | 40 | 6 | | 55 |
| Phytoplankton | *Tetrastrum staurogeniaeforme* | Chlorophyta | 2008 | 36.35 | 44 | 6 | | 66 |
| Phytoplankton | *Tetrastrum staurogeniaeforme* | Chlorophyta | 2009 | 201.95 | 35 | 6 | | 43 |
| Phytoplankton | *Tetrastrum staurogeniaeforme* | Chlorophyta | 2010 | 12.12 | 82 | 6 | | 95 |
| Phytoplankton | *Tetrastrum staurogeniaeforme* | Chlorophyta | 2011 | 11.29 | 63 | 6 | | 85 |
| Phytoplankton | *Trachelomonas volvocina* | Euglenophyta | 2004 | 4.39 | 56 | 1 | | 89 |
| Phytoplankton | *Ulothrix sp. 1* | Chlorophyta | 1996 | 8.77 | 51 | 2 | | 77 |
| Phytoplankton | *Ulothrix sp. 1* | Chlorophyta | 2000 | 17.36 | 65 | 2 | | 81 |
| Phytoplankton | *Uroglena sp. 1* | Chrysophyta | 1995 | 52.09 | 44 | 10 | | 62 |
| Phytoplankton | *Uroglena sp. 1* | Chrysophyta | 1998 | 32.55 | 44 | 10 | | 61 |
| Phytoplankton | *Uroglena sp. 1* | Chrysophyta | 1999 | 223.23 | 26 | 10 | | 36 |
| Phytoplankton | *Uroglena sp. 1* | Chrysophyta | 2000 | 972.29 | 17 | 10 | | 21 |
| Phytoplankton | *Uroglena sp. 1* | Chrysophyta | 2001 | 1244.30 | 23 | 10 | | 31 |
| Phytoplankton | *Uroglena sp. 1* | Chrysophyta | 2003 | 9.09 | 45 | 10 | | 88 |
| Phytoplankton | *Uroglena sp. 1* | Chrysophyta | 2005 | 60.58 | 40 | 10 | | 61 |
| Phytoplankton | *Uroglena sp. 1* | Chrysophyta | 2006 | 15.15 | 52 | 10 | | 80 |
| Phytoplankton | *Uroglena sp. 1* | Chrysophyta | 2007 | 245.37 | 34 | 10 | | 47 |
| Phytoplankton | *Uroglena sp. 1* | Chrysophyta | 2009 | 25.96 | 63 | 10 | | 78 |
| Phytoplankton | *Urosolenia longiseta* | Bacillariophyta | 2009 | 18.18 | 68 | 1 | | 84 |
| Phytoplankton | *Woronichinia naegeliana* | Cyanophyta | 1995 | 11127.30 | 10 | 17 | | 14 |
| Phytoplankton | *Woronichinia naegeliana* | Cyanophyta | 1996 | 12055.45 | 7 | 17 | | 11 |
| Phytoplankton | *Woronichinia naegeliana* | Cyanophyta | 1997 | 5208.80 | 8 | 17 | | 13 |
| Phytoplankton | *Woronichinia naegeliana* | Cyanophyta | 1998 | 10292.37 | 7 | 17 | | 10 |
| Phytoplankton | *Woronichinia naegeliana* | Cyanophyta | 1999 | 9983.35 | 9 | 17 | | 13 |
| Phytoplankton | *Woronichinia naegeliana* | Cyanophyta | 2000 | 1736.20 | 16 | 17 | | 20 |
| Phytoplankton | *Woronichinia naegeliana* | Cyanophyta | 2001 | 2922.55 | 13 | 17 | | 18 |
| Phytoplankton | *Woronichinia naegeliana* | Cyanophyta | 2002 | 325.54 | 19 | 17 | | 39 |
| Phytoplankton | *Woronichinia naegeliana* | Cyanophyta | 2003 | 3635.08 | 9 | 17 | | 18 |
| Phytoplankton | *Woronichinia naegeliana* | Cyanophyta | 2004 | 6144.51 | 7 | 17 | | 11 |
| Phytoplankton | *Woronichinia naegeliana* | Cyanophyta | 2005 | 2743.09 | 12 | 17 | | 18 |
| Phytoplankton | *Woronichinia naegeliana* | Cyanophyta | 2006 | 18675.21 | 5 | 17 | | 8 |
| Phytoplankton | *Woronichinia naegeliana* | Cyanophyta | 2007 | 1999.29 | 15 | 17 | | 21 |
| Phytoplankton | *Woronichinia naegeliana* | Cyanophyta | 2008 | 7383.75 | 11 | 17 | | 16 |
| Phytoplankton | *Woronichinia naegeliana* | Cyanophyta | 2009 | 1097.23 | 23 | 17 | | 28 |
| Phytoplankton | *Woronichinia naegeliana* | Cyanophyta | 2010 | 665.66 | 34 | 17 | | 40 |
| Phytoplankton | *Woronichinia naegeliana* | Cyanophyta | 2011 | 6351.93 | 10 | 17 | | 14 |
